# c-kit expression profile and regulatory factors during spermatogonial stem cell differentiation

**DOI:** 10.1186/1471-213X-13-38

**Published:** 2013-10-27

**Authors:** Lei Zhang, Jiangjing Tang, Christopher J Haines, Huai Feng, Liangxue Lai, Xiaoming Teng, Yibing Han

**Affiliations:** 1 Shanghai first maternity and infant health hospital, Tongji University, Shanghai, China; 2 Department of Obstetrics and Gynaecology, Prince of Wales hospital, The Chinese University of Hong Kong, Hong Kong, China; 3 Diagnostics and Reproductive Health Center BGI, Beishan Industrial Zone, Yantian District, Shenzhen, 518083, China; 4 Guangzhou Institute of Biomedicine and Health Chinese Academy of Sciences, Guangzhou, China; 5 Department of Obstetrics and Gynecology, New York University Medical College, Manhasset, NY, USA

**Keywords:** *c-kit*, Expression profile, Spermatogonial stem cells, Differentiation, Regulatory factor

## Abstract

**Background:**

It has been proven that *c-kit* is crucial for proliferation, migration, survival and maturation of spermatogenic cells. A periodic expression of *c-kit* is observed from primordial germ cells (PGCs) to spermatogenetic stem cells (SSCs), However, the expression profile of *c-kit* during the entire spermatogenesis process is still unclear. This study aims to reveal and compare *c-kit* expression profiles in the SSCs before and after the anticipated differentiation, as well as to examine its relationship with retinoic acid (RA) stimulation.

**Results:**

We have found that there are more than 4 transcripts of *c-kit* expressed in the cell lines and in the testes. The transcripts can be divided into short and long categories. The long transcripts include the full-length canonical *c-kit* transcript and the 3′ end short transcript. Short transcripts include the 3.4 kb short transcript and several truncated transcripts (1.9-3.2 kb). In addition, the 3.4 kb transcript (starting from intron 9 and covering exons 10 ~ 21) is discovered to be specifically expressed in the spermatogonia. The extracellular domain of Kit is obtained in the spermatogonia stage, but the intracellular domain (50 kDa) is constantly expressed in both SSCs and spermatogonia. The *c-kit* expression profiles in the testis and the spermatogonial stem cell lines vary after RA stimulation. The wave-like changes of the quantitative expression pattern of *c-kit* (increase initially and decrease afterwards) during the induction process are similar to that of the *in vivo* male germ cell development process.

**Conclusions:**

There are dynamic transcription and translation changes of *c-kit* before and after SSCs’ anticipated differentiation and most importantly, RA is a significant upstream regulatory factor for *c-kit* expression.

## Background

Spermatogenesis starts from diploid spermatogonial stem cells (SSCs). The SSCs, also known as type A single (A_s_) Spg, are located on the basement membrane of the seminiferous tubules. The A_s_ Spg can self-renew or produce the type A paired (A_p_) Spg. After successive divisions, A_p_ Spg differentiates, forms chains of 4, 8 or 16 aligned Spg (A_al_) and migrates along the basement membrane. A_al_ Spg differentiates into A1 Spg that further divides and differentiates into A2, A3, A4intermediates and B Spg, which undergoes meiosis after a final mitosis stage [1]. The “undifferentiated” (A_s_, A_p_ and A_al_) and the “differentiating” (A1, A2, A3, A4, intermediate and B) Spg differ in the expression profiles of *c-k t*[2]. *c-kit* is allelic to the W locus on mouse chromosome 5 [3]. The 21-exon gene encodes for a 5150 bp transcript, which is translated into a product of 145 kDa protein with 979 amino acid residues. This product is known as Kit [4]. Kit transduces growth regulatory signals across the plasma membrane and has three main functional regions, the extracellular, the transmembrane and the intracellular domains [5,6]. Its transcription process is only activated after binding with Kitl expressed by the Sertoli cells. The Kit/Kitl pathway is considered to be crucial for the proliferation, migration, survival and maturation of the germ cells [7-18]. In spite of the 5.1 Kb full-length canonical transcript, two alternative mRNAs of *c-kit*, 3.2 and 2.3 kb in length, exist in the haploid cells of the mouse testis [19]. With an Open Reading Frame (ORF) that starts in the intron 16 of the mouse *c-kit*, an alternative spermatid-specific *c-kit* transcript contains all of the downstream exons (including 12 hydrophobic amino acids followed by the last 190 carboxyl terminal residues), encodes for Tr-Kit (~30 kDa) [7,20,21]. The 30 kDa Tr-Kit is found in the residual sperm cytoplasm and it has evident functions in the activation of oocyte during fertilization in mice [21,22].

*c-kit* has been a marker for SSCs pluripotency lost and its expression continues until meiosis is initiated [2,18]. The expression of protein Kit in the male germ cells is contradictory to those of gene *c-kit*. In early studies, Kit expression is detected in type A (A1–A4), intermediate, type B spermatogonia, as well as preleptotene spermatocytes, but not in the undifferentiated spermatogonia [2,18]. More recent studies demonstrate that Kit is also expressed A_s_, A_pr_ and A_al_. Therefore, whether Kit is expressed in spermatogonia and whether Kit/Kitl activation is a prerequisite for differentiation or not remain to be a question [23-28]. Even though the inactivation of *c-kit* by its specific inhibitor Imatinib results in Spg self-renewal impairment [29], both Kit^-^ and Kit^+^ spermatogonia have exhibited stem cell activities as evaluated by intra-seminiferous transplantation [1,24,30]. The POU5F1^+^/Kit^+^ subset of mouse SSCs can differentiate into several lines of somatic cells except for sperm cells [31].

We hypothesize that the expression profiles of *c-kit* in the male germ cells during spermatogenesis are dynamically changed before and after the expected differentiation, and these changes are important for their functional responses to the spermatogenesis-related genes. In this study, we have investigated the expression of *c-kit* in the immortal cell lines representing the SSCs, the differentiating spermatogonia and spermatocytes in hopes of understanding its natural expression patterns. We have also compared the *c-kit* expression patterns in those cell lines with their corresponding stage testes. The cell line c18-4 and 5 dpp mouse testes (before the initiation of spermatogonia differentiation) represent the undifferentiated spermatogonia. CRL-2053 and 10 dpp mouse testes (after the initiation of spermatogonia differentiation) represent the differentiating spermatogonia. CRL-2196 cells represent primary spermatocytes. The 60 dpp testes represent a mixture of the undifferentiated, the differentiating, the maturing and the matured germ cells.

RA, an active metabolite of vitamin A, is a vital signaling molecule for normal fetal development, pattern formation, cell proliferation, differentiation and apoptosis [32,33]. RA is considered to be crucial for germ cells to undergo meiosis in both male and female [34,35]. Testes of adult vitamin A-deficient mice/rat have seminiferous tubules that only contain Sertoli cells, type A spermatogonia and few preleptotene spermatocytes. With a reduced *c-kit* expression or without *Stra8* expression, the type A spermatogonia will arrest before differentiation (before A1 stage spermatogonia) [36]. Administration of vitamin A to these animals results in a synchronized spermatogenesis emerging from type A spermatogonia and an enhanced expression of *c-kit*[37]. Therefore, RA is a key regulatory factor for *c-kit* expression.

## Methods

### Cell lines and animals

The c18-4 cell line represents the mouse SSCs [38]. CRL-2053 (ATCC) is a type B spermatogonia cell line [39].CRL-2196 (GC-2spd(ts), ATCC) is a spermatocyte cell line [40,41]. C57/BL6 mice at different ages were purchased from laboratory animal service center (LASEC), The Chinese University of Hong Kong. All procedures were approved by the Animal Research Ethics Committee of the University.

### Cell culture

All cells were cultured in the Dubecco modified eagle medium/F12 (DMEM/F12, Invitrogen, Carlsbad, CA, USA) supplemented with 10% fetal bovine serum (FBS, Invitrogen, Carlsbad, CA, USA). A subcultivation ratio of 1:6 to 1:10 was applied. Media were renewed 1 to 2 times per week. The cells were frozen in complete growth medium supplemented with 5% (v/v) DMSO and stored in liquid nitrogen.

### Mouse testes collection

Mice at 5 days post partum (dpp), 10 dpp and 60 dpp were sacrificed by cervical dislocation. For RNA extraction, testes were washed twice with phosphate buffered saline (PBS) and then immersed in “RNA-later” stabilization reagent (Qiagen, Valencia, CA, USA). Before protein extraction, testes were washed twice with PBS, transported in iceboxes and stored in −80°C. Three batches of animals were used for each experiment.

### *In vitro* tissue culture and RA induction

*In vitro* tissue culture was carried out according to the methods described by previous study [42]. Testes from 5 dpp, 10 dpp and 60 dpp mice were detunicated, cut into small pieces per testis, placed on Millicell CM filters (Millipore, Bedford, MA, USA) floating on the surface of medium and covered with drops of medium (DMEM/F12 + 10% FBS). RA (Sigma-Aldrich Co., Saint Louis, MO, USA) diluted in ethanol was added to the culture medium to make a final a concentration of 0.7 μM or 2 μM. Tissues were harvested after 24 hours of RA treatment. Total RNA was isolated using the RNeasy mini kit (Qiagen, Valencia, CA, USA).

### *In vitro* cultured germ cells and RA induction

For germ cell exclusive induction assay, 2 × 10^6^ c18-4 or CRL-2063 cells were pre-seeded into T25 cell culture flasks separately (2 flasks each group) overnight before the treatment in full medium (DMEM/F12 + 10% FBS). Induction media (DMEM/F12 + 10% FBS) with a final concentration of 2 μM RA dissolved in ethanol were used in the treatment (induction) group. The same amount of ethanol without RA medium was set up as the control group. After 24 hours of induction, the induction media was removed, cells were washed with PBS twice, and cells were collected and stored at −80°C until analysis. Three independent replications were carried out for each experiment.

### Methods for RNA preparation, electrophoresis and Northern blot

Total RNA from cells and testes was isolated using the RNeasy mini kit (Qiagen, Valencia, CA, USA) following the manufacturer’s instructions. Sizes of RNA were estimated by comparing with 2 μg RNA Millennium size markers (Ambion, Austin, TX, USA) by measuring the distance from each band to the loading well.

DNA fragments corresponding to exons 10–12 and exons 18–20 of the full-length *c-kit* transcript were obtained by PCR with *c-kit* specific primers using the 60 dpp mouse testis cDNA as the template. Primers sequences are shown in Table 1. Amplified DNA fragments were inserted into the Topo-TA vector (Invitrogen, Carlsbad, CA, USA). The plasmids were then extracted by QIAprep spin miniprep kit (Qiagen, Valencia, CA, USA) and were sent to commercial company for sequencing.

**Table 1:** **PCR primers, real-time PCR primers and RNA probe sequence of mouse *c-kit *gene**

**Probe name**	**Probe sequence (5′ → 3′)**
PCR primers	
Exons 10-12	Sense 5′ -TGGGGATCATTGTGATGGT-3′
	Anti-sense 5′-ATGGCAGCATCCGACTTAAT-3′
Exons 18-20	Sense 5′ - CCTCTGGGAGCTCTTCTCCT-3′
	Anti-sense 5′- GCTGTCCGAGATCTGCTTCT-3′
Real-time PCR primers	
Exon 7-8	AACGTTTACGT GAACACAAAACCAG
Exon 20-21	GCACCAAGCACATTTACTCCAACTT
Exon 21+	CTGATATGTTGTCCAACTGTTGACA
Exons 10-12 probe (extracellular domain)	ATGGCAGCATCCGACTTAATCAAGCCATATGCAGTGGCCTCAACGACC
	TTCCCGAAGGCACCAGCTCCCAATGTCTTTCCAAAACTCAGCCTGTTTC
	TGGGAAACTCCCATTTGTGATCATAAGGAAGTTGCGTCGGGTCTATGT
	AAACATAATTGTTTCCATTTATCTCCTCGACAACCTTCCATTGTACTTC
	ATACATGGGTTTCTGCAAATATTTGTAGGTGAGCAC CATCACAATG
	ATCCCCAT
Exons 18-20 probe (intracellular domain)	GCTGTCCGAGATCTGCTTCTCAATAAGTTGGACAACCTGCTTGAATGT
	TGGCCTTTTCAAGGGGTCAGCGTCCCAGCAAGTCTTCATGAC
	GTCATACATTTCGGCAGGCG CGTGCTCCGG GCTGACCATC
	CGGAAGCCTTCCTTGATCATCTTGTAGAACTTGGAGTCGACCGGCATC
	CCTGGGTAGGGGCTGCTTCCTAAGGAGAAGAGCTCCCAGAGG

Exons 10-12 probe hybridizes to *c-kit* extracellular domain coding area. Exons 18-20 probe hybridizes to *c-kit* intracellular domain coding area.

RNA probes were prepared by MAXIscript kit (Ambion, Austin, TX, USA) following the manufacturer’s instructions. mRNA-complementary (antisense) transcripts were synthesized in a 20 μl *in vitro* transcription system containing 1 μg DNA template, 2 μl 10 × transcription buffer, 1 μl 10 mM ATP, 1 μl 10 mM CTP, 1 μl 10 mM GTP, 5 μl 800 Ci/mmol [α-32P] UTP at a concentration of 10 mCi/mL (Perkinelmer, San Jose, CA, USA) and 2 μl T3 enzyme mix. After purification with NucAway Spin columns (Ambion, Austin, TX, USA), the RNA probes were hybridized with the blots with RNA samples in the ULTRAhyb ultrasensitive hybridization buffer (Ambion, Austin, TX, USA) at 68°C overnight. The same blot was stripped and re-probed with α^32^P-labeled beta-actin RNA probe as internal control. Northern hybridization was performed twice with probes and membranes that were made independently. The sequences of PCR primers and RNA probes are shown in Table 1.

### Rapid amplification of cDNA ends (RACE), cloning and sequencing

The number and size of *c-kit* mRNA expressed in mice cell lines and testis were determined by the Northern blot, the existence of these transcripts were further confirmed by RACE and sequencing. We used the BD-Smarter RACE protocol from BD Biosciences Clontech (Paloalto, CA, USA) in RACE analysis. The full-length cDNAs was made by joint action of the SMARTer II A Oligonucleotide and SMARTScribe Reverse Transcriptase (a variant of MMLV RT) in reverse transcription reactions. The first strand of cDNA synthesis was obtained from 1 μg total RNA. PCR amplification was done with specific primers hit exons 10–12 and exons 18–21 on the full-length *c-kit* transcript (Table 2) in conjunction with universal primers that were provided in the kit. Advantage 2 PCR kit (Clontech, Paloalto, CA, USA) was used for the 5′ and 3′ PCR amplification. Nested PCR and touchdown PCR were used to safeguard the specificity of the amplification. Electrophoresis of the PCR products, bands cutting and gel extraction (QIAquick gel extraction kit; QIAGEN, Valencia, CA, USA) were performed. All of the clear RACE PCR product gel extractions were cloned to TA vector (TOPO TA cloning kit for sequencing, Invitrogen, Carlsbad, CA, USA) and sent to commercial company for sequencing. 5′ and 3′ RACE results were combined to obtain the full-length *c-kit* transcripts sequence information.

**Table 2:** RACE primers

**Name**	**5′ or 3′**	**Sequence (5′ → 3′)**	**No. of bases**	**Exons hitting**	**Position on NM_021099**
e11 5′	5′	CAGCCTGTTTCTGGG AAACTCCCATTTG	27	Exon 11	1825-1798
e12 5′	5′	GCAACTGTCATGGC AGCATCCGACTT	26	Exon 12	1920-1895
e18 5′	5′	TGCTCTCTGGTGCCA TCCACTTCAC	25	Exon 18	2552-2756
e20 5′A	5′	GGTCAGCGTCCCAG CAAGTCTTCAT	25	Exon 20	2786-2762
e20 5′B	5′	AAGGGGTCAGCGTC CCAGCAAGTCT	25	Exon 20	2790-2766
e20 5′C	5′	TGCTTGGTGCTGTCC GAGATCTGCT	25	Exon 20	2856-2832
e21 5′	5′	GGGGTTGCAGTTTG CCAAGTTGGAG	25	Exon 21	2887-2863
e10 3′	3′	AAATCCAGGCCCAC ACTCTGTTCACG	26	Exon 10	1602-1627
e11 3′	3′	TGGGAGTTTCCCAG AAACAGGCTGAG	26	Exon 11	1802-1827
e18 3′	3′	CCGTGAAGTGGATG GCACCAGAGAG	25	Exon 18	2550-2574
e19 3′A	3′	AGGAAGCAGCCCCT ACCCAGGGATG	25	Exon 19	2650-2674
e19 3′B	3′	GGGATGCCGGTCGA CTCCAAGTTCT	25	Exon 19	2669-2693
e20 3′A	3′	TGACCCCTTGAAAA GGCCAACATTCA	26	Exon 20	2782-2807
e20 3′B	3′	GCAGATCTCGGACA GCACCAAGCAC	25	Exon 20	2833-2857

Requirement of a good gene specific primer for RACE: It should be 23-28 nt, has 50-70% GC and Tm > 70°C, and does not complement to the 3′ of the Universal Primer Mix.

### Quantitative real-time RT-PCR

Total RNA (2 μg) was treated with DNase I (Sigma, Saint Louis, USA) for 15 minutes at room temperature and then reversely transcribed by High Capacity cDNA Reverse Transcription Kit (Applied Biosystems, Foster City, CA, USA).

Real-time RT-PCR analysis of *c-kit* was performed with Taqman universal PCR master mix and Taqman gene expression assays on the ABI Prism 7900HT Real Time PCR System, according to the manufacturer’s instructions (Applied Biosystems, Foster City, CA, USA). The relative expression level of each target gene was calculated by the comparative CT method and was normalized to glyceraldehyde 3-phosphate dehydrogenase (GAPDH) expression. Three *c-kit* gene-specific probes that hit different parts of the full-length transcript (exon 7–8, exon 20–21 and exon 21^+^) were used.

Real-time RT-PCR analysis for other genes were performed with Power SYBR PCR master mix and gene specific primers on the ABI Prism 7900HT Real Time PCR System, according to the manufacturer’s instructions (Applied Biosystems, Foster City, CA, USA). The relative expression level of each target gene was calculated by the comparative CT method and was normalized to GAPDH expression. The primers of the candidate genes are list in Table 3.

**Table 3:** Gene specific primers of the candidate genes

**Name**	**Forward**	**Reverse**
BMP4	TTCCTGGTAACCGAATGCTGA	CCTGAATCTCGGCGACTTTTT
Cyp26b1	GCAAGATCCTACTGGGCGAAC	TTGGGCAGGTAGCTCTCAAGT
DAZL	GTCCTTACATGTACCATTCTGTGAC	GACTCCAACAAAACAGCAGACAA
EGR 3	AGCTGAACTGGGCTGTGTCT	AATGGGGAGTGGGTATGTGA
Kitl	TCTGCGGGAATCCTGTGACT	TGGAAGATTTGCCACCAGTTT
PLZF	GCAAGAACAGCGTCAAGACA	TGGGATCACGTGAAGCTATG
RARα	TCCGAAGAGATAGTACCCAGC	AAAGCAAGGCTTGTAGATGCG
Stra8	GTTTCCTGCGTGTTCCACAAG	CACCCGAGGCTCAAGCTTC

Each RT-PCR analysis was repeated 3 times after GAPDH normalization.

### Western blot

Cells and testis tissues were lysed on ice in RIPA buffer containing 1% freshly added protease inhibitors. Protein electrophoresis and gel bolting were performed with NuPAGE electrophoresis system (Invitrogen, Carlsbad, CA, USA) following the manufacturer’s instructions. The blotted PCDF membranes were blocked with 5% (wt/vol) non-fat dry milk (RT, 60 minutes) and probed for Kit at 4°C overnight, using either 1 μg/ml of a monoclonal antibody (rat anti-mouse; NOVUS, Littleton, CO, USA) directed against the extracellular domain of the Kit or a polyclonal antibody (rabbit anti-human, mouse, rat; NOVUS, Littleton, CO, USA) directed against the amino acid near S715 of the human Kit (1 μg/ml) followed by the HRP-conjugated secondary antibodies (Santa Cruz, Santa Cruz, CA, USA) staining.

Protein lysate from Kit expressed in human megakaryoblast cell lines (ATCC no. CRL-2021) was set up as positive control and protein lysate from Kit negative mouse myoblast cell line (ATCC no. CRL-1772) was set up as negative control. The same blot was stripped and re-probed with mouse beta-actin primary antibody (Santa Cruz, Santa Cruz, CA, USA) as internal control.

### Immunofluorescence staining of cells

The cover slips with the cells were washed 3 times with PBS again and were incubated with 5% normal goat serum (Santa Cruz, Santa Cruz, CA, USA) in PBS for 30 minutes before being incubated with the primary antibody overnight at 4°C. The cells were then incubated with the secondary antibody and mounted with UltraCruz™ Mounting Medium with DAPI (SantaCruz, Santa Cruz, CA, USA). The following antibodies were used in this study: the FITC monoclonal rat-anti-mouse Kit extracellular domain (1:200, 105805, BioLegend, San Diego, CA, USA), the monoclonal rat-anti-mouse Kit extracellular domain (1:200, NBP1-43359, NOVUS, Littleton, CO, USA), the monoclonal rat-anti-mouse Kit extracellular domain (1:100, KJ-14, Santa Cruz, Santa Cruz, CA, USA); the polyclonal rabbit-anti-human/mouse/rat Kit intracellular domain (1:200, NBP1-19865, NOVUS, Littleton, CO, USA), the polyclonal goat-anti-mouse Kit C-terminus (1:100, M14, Santa Cruz, USA) and the polyclonal rabbit-anti-human/mouse Kit C-terminus (1:100, C19, Santa Cruz, Santa Cruz, CA, USA). The secondary antibodies used in this study included: the Alexa 488-conjugated goat-anti-rat IgG (1:500; Invitrogen, Carlsbad, CA, USA); the Alexa 594-conjugated goat-anti-rabbit IgG (1:500; Invitrogen, Carlsbad, CA, USA) and the Texas red-conjugated donkey-anti-goat IgG (1:100; Santa Cruz, Santa Cruz, CA, USA).

### Statistical analysis

Statistical analysis was performed by unpaired two-tail student t test using SPSS software (Version 17.0). All experiments were performed for at least three independent times and a P value of less than 0.05 was considered statistically significant.

## Results

### Transcription of *c-kit* in cell lines and testes

**Figure 1:**
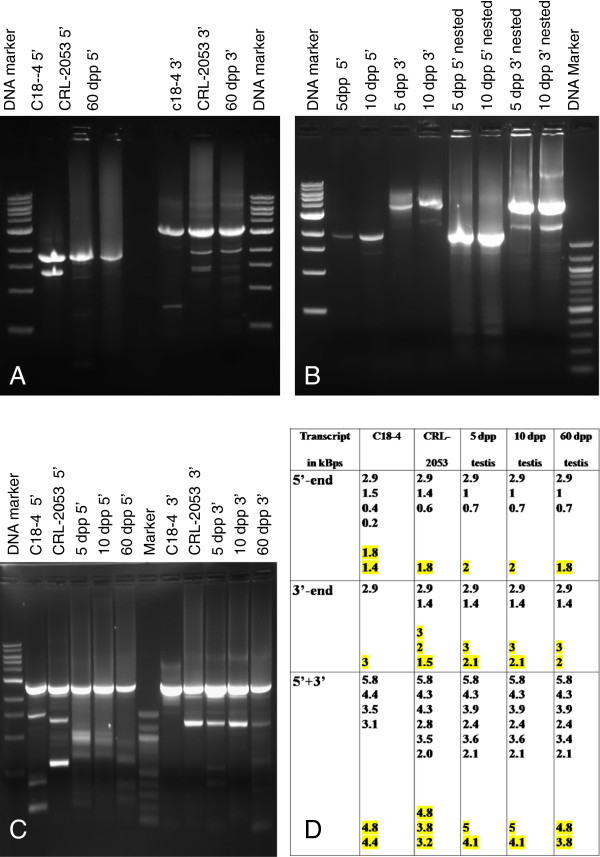
***c-kit *northern hybridization. (A)** RNA electrophoresis. 10 μg total RNA from different samples were loaded. RNA sizes were marked with 2 μg (2 μl) RNA Millennium size markers (Ambion). All RNA samples were in good qualities. **(B)** Northern hybridization with *c-kit* probe hit exons 10-12. Short transcripts (purple arrowhead) with a size between 4 and 5 kb could be observed in c18-4, CRL-2053 and different aged testes. Long transcripts (blue arrowhead) with a size between 5 and 6 kb were seen in 10 dpp and 60 dpp testes. **(C)** Northern hybridization with *c-kit* probe hit exons 18-20. A 1.5 kb short transcript (red arrowhead) was observed in c18-4 and a 2.7 kb short transcript (green arrowhead) was observed in CRL-2053. Short transcripts (purple arrowhead) with a size between 4 and 5 kb could be observed in 5 dpp, 10 dpp and 60 dpp testes. Long transcripts (blue arrowhead) with a size between 5 and 6 kb were seen in 10 dpp and 60 dpp testes. **(D)** Northern hybridization of beta-actin (internal control).

**Figure 2:**
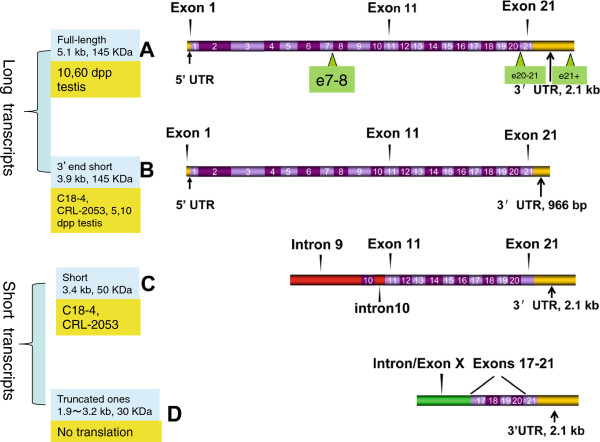
**RACE PCR products gel electrophoresis. (A)** 5′ and 3′ RACE with primer sets hit exons 10-12. c18-4 had two 5’ ends (1.8 kb and 1.4 kb). CRL-2053 and 60 dpp testes had one 5’ end (1.8 kb). c18-4 had one 3 kb 3’ end (3 kb). CRL-2053 and 60 dpp testes had two 3’ end (3 kb and 1.8 kb). **(B)** 5′ and 3′ RACE with primer sets hit exons 10-12. 5 dpp and 10 dpp testis had one 5’ ends (2 kb). 5 dpp and 10 dpp testes had two 3’ end (3 kb and 2.1 kb). CRL-2053 and 60 dpp testes had two 3’ end (3 kb and 1.8 kb). **(C)** 5′ and 3′ RACE with primer sets hit exons 18-21. c18-4 had two 5’ ends (2.9 kb and 1.5 kb). CRL-2053 had three 5’ ends (2.9 kb, 1.4 kb and 1.6 kb). 5 dpp, 10 dpp and 60 dpp testes had the same one 5’ end (2.9 kb); c18-4 had one 3’ end (2.9 kb). CRL-2053 5 dpp and 10 dpp and 60 dpp testes had two 3’ end (3 kb and 1.8 kb). **(D)** Summary of c-kit RACE PCR fragments from cell lines and testis.

**Figure 3:**
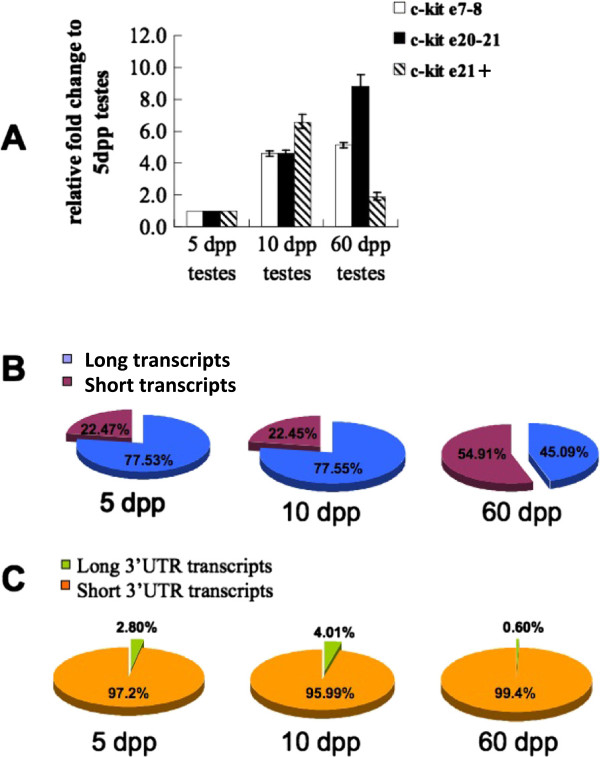
***c-kit *transcripts before and after differentiation of SSCs. (A)** Full-length transcript. This transcript was composed of 21 exons and with a size about 5.2 kb. A 2.1 kb 3′ UTR region located at exon 21 of this transcript and it was expressed by SSCs before and after differentiation. Green triangles on this figure represented 3 groups of *c-kit* primers that hit exons 7-8, exons 20-21 and exon 21 respectively. **(B)** Short 3′ UTR transcript (4 kb). This transcript was composed of 21 exons with 966 bp long 3’ UTR. **(C)** Short transcript (2.7 kb) started from intron 9 of the full-length transcript. **(D)** The truncated transcript (1.5 kb). It represented a group of transcripts that started from different intron or exon of *c-kit* gene, the Intron/Exon X means the beginning of these transcripts. We found some of these transcripts started from exon 13, exon 15 and exon 17 of the *c-kit* gene respectively. All these truncated transcripts we found containing a conserved domain from exon 17 to exon 21.

Northern blots revealed at least 4 transcripts in the cells and testes (Figure 1B and C). Even more *c-kit* transcripts expressed in c18-4, CRL-2053 and 5 dpp, 10 dpp and 60 dpp testes were assayed by RACE (Figure 2A, B, C, D). The 1.5 kb transcript expressed in the c18-4 cells was not shown by RACE (Figure 1C). Four representative transcripts (Type A, B, C and D) were illustrated in Figure 3 and a multiple blast of their sequences was shown in Additional file 1. Quantitative expression discrepancies (either in the 5′ end or in the 3′ end) of *c-kit* among the cell lines and testes of different stages existed. Multiple blast assays demonstrated that exons 17–21 region was highly conserved in the *c-kit* transcripts. The four representative transcripts included the full-length canonical transcript (transcript A, 5.1 kb, expressed in 10 and 60 dpp testes), the 3′ end short transcript (transcript B, 3.9 kb, expressed in c18-4, CRL-2053, 5 and 10 dpp testes), the short transcript (transcript C, 3.4 kb, expressed in CRL-2053) and the truncated transcripts (transcripts D, 1.9-3.2 kb, no expression detected by Northern blot analysis) respectively (Figure 3).

### Quantitative analysis of different transcript expressions in testes and germ cell lines

Three sets of specific primers for RT-PCR were designed to detect *c-kit* transcripts (e7-8, e20-21 and e21^+^ as indicated by triangles in Figure 3). Products from the e7-8 primers represented the long transcripts (transcripts A + B). Products from the e20-21primers represented the total transcripts (transcripts A + B + C + D). The ratio of the long transcripts could be calculated as (e7-8/e20-21) × 100%. The ratio of the short transcripts (Transcript C + D) was consequently (1-ratio_long transcripts_) × 100%. Those produced by e21^+^ primers represented all transcripts with a long 3′ end except for transcript B (Transcripts A + C + D). The ratio of the long 3′ UTR transcripts was (e21^+^/e20-21) × 100%. The ratio of the short 3′ UTR transcripts was (1-ratio_long 3′ UTR transcripts_) × 100%.

**Figure 4:**
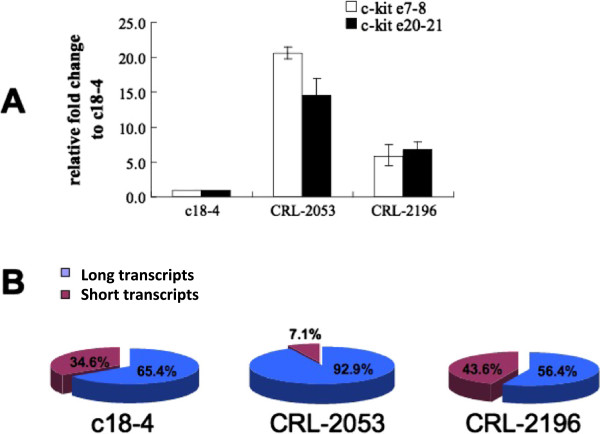
**Profiles of *c-kit *transcripts in 5 dpp, 10 dpp and 60 dpp testes. (A)** Relative *c-kit* mRNA expression level in the 5 dpp, 10 dpp and 60 dpp mouse testis. Error bars represent S.E.M. *c-kit* e7-8, real-time PCR using probe hit full-length *c-kit* transcripts on exons 7-8. *c-kit* e20-21, real-time PCR using probe hit full-length *c-kit* transcripts on exons 20-21. *c-kit* e21, real-time PCR using probe hit full-length *c-kit* transcripts on the end of exons 21. Differences between each two groups were significant (P<0.05). **(B)** The ratio of Full-length and truncated *c-kit* transcripts in 5 dpp, 10 dpp and 60 dpp mouse testes. The ratio of truncated *c-kit* transcripts were calculated by the formula (1-ratio_full-length transcripts_) × 100%. **(C)** The ratio of long 3′ UTR and short 3′ UTR *c-kit* transcripts in 5 dpp, 10 dpp and 60 dpp mouse testes. The ratio of short 3′ UTR *c-kit* transcripts were calculated by the formula (1-ratio_long 3′UTR transcripts_) × 100%.

From 5 dpp to 10 dpp, expressions of both transcripts A + B and transcripts C + D increased significantly (Figure 4A, B). The ratios of long and short transcripts remained constant (22.47% in 5 dpp and 22.45% in 10 dpp, Figure 4B). The ratio of long 3′ transcripts jumped from 2.8% to 4.01% (Figure 4C). From 10 dpp to 60 dpp, transcripts A + B + C + D increased moderately but transcripts A + C + D decreased. Hence, it can be deduced that transcript B must have been enhanced significantly (Figure 4A). Consequently, since the relative amount of transcripts A + B did not change, the quantity of transcript A must have declined dramatically. The ratio of short transcripts progressed from 22.45% in 10 dpp to 54.91% in 60 dpp (Figure 4B). Long 3′ transcripts dropped from 4.01% (10 dpp) to 0.60% (60 dpp) (Figure 4C).

**Figure 5:**
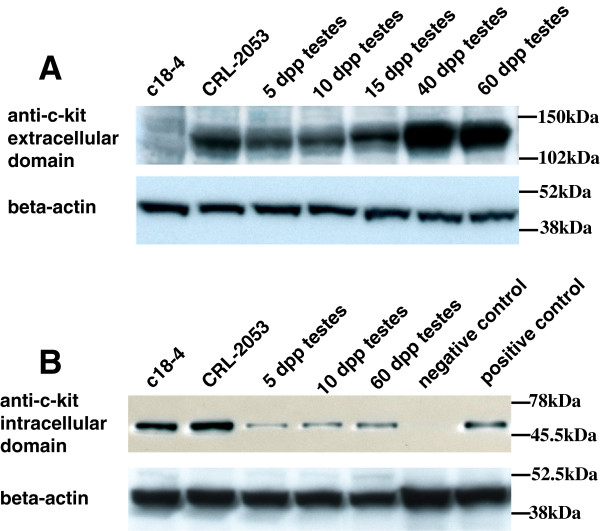
***c-kit *transcripts profile in germ cell lines. (A)** Relative *c-kit* mRNA expression level in the c18-4, CRL-2053 and CRL-2196 germ cell lines. Error bars represent S.E.M. *c-kit* e7-8 represent full-length *c-kit* transcripts. *c-kit* e20-21 represent total *c-kit* mRNA. Differences between each two groups were significant (P<0.05). **(B)** The ratios of Full-length and truncated *c-kit* transcripts in c18-4, CRL-2053 and CRL-2196 germ cell lines. The ratio of truncated *c-kit* transcripts were calculated by the formula (1-ratio_full-length transcripts_) × 100%.

Primers e21^+^ did not detect any bands in all three types of cell lines. As a result, all transcripts in these cell lines lacked of the 3′ UTR ends. The relative quantity of transcript A was equivalent with transcript B in the cell lines. The expression of type A + B multiplied significantly in CRL-2053 (about 20 folds of that in c18-4) and then diminished (about 5 folds of that in c18-4) in CRL-2196 (Figure 5A). The ratio of type A + B transcripts was 65%, 92.9% and 56.4% in c-18-4, CRL-2053 and CRL-2196 respectively (Figure 5B).

### Translation of *c-kit* in the testes and male germ cell lines

**Figure 6:**
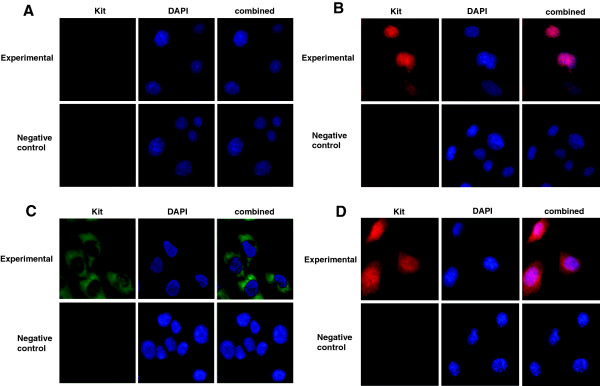
**Western blot analysis of *c-kit *protein expression in germ cell lines and testes. (A) ***c-kit* protein expression assayed by an anti-mouse Kit extracellular domain monoclonal antibody in germ cell lines and testes. **(B)***c-kit* protein expression assayed by an anti-mouse Kit intracellular domain monoclonal antibody in germ cell lines and testes.

**Figure 7:**
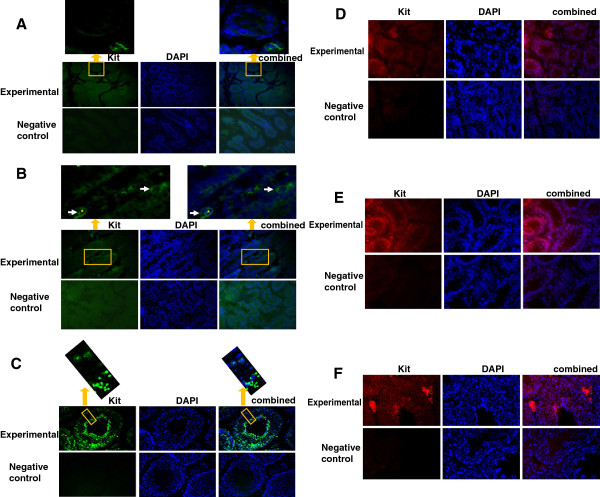
**Immunofluorescence staining of Kit expressed in c18-4 and CRL-2053.** Images were obtained with ZEISS Axioplan 2 imaging system and Spot 4.7/SpotAdvanced software (Magnification × 400). Kit positive cells were stained with green or red fluorescein (arrow). The cell nuclei was counterstained with 4′,6-diamidino-2-phenylindole. Images were merged with Photoshop CS4. **(A)** FITC labeled anti-Kit extracellular domain in c18-4. **(B)** Alexa Fluo 594 labeled anti-Kit intracellular domain in c18-4. **(C)** FITC labeled anti-Kit extracellular domain in CRL-2053. **(D)** Alexa Fluo 594 labeled anti-Kit intracellular domain in CRL-2053.

**Figure 8:**
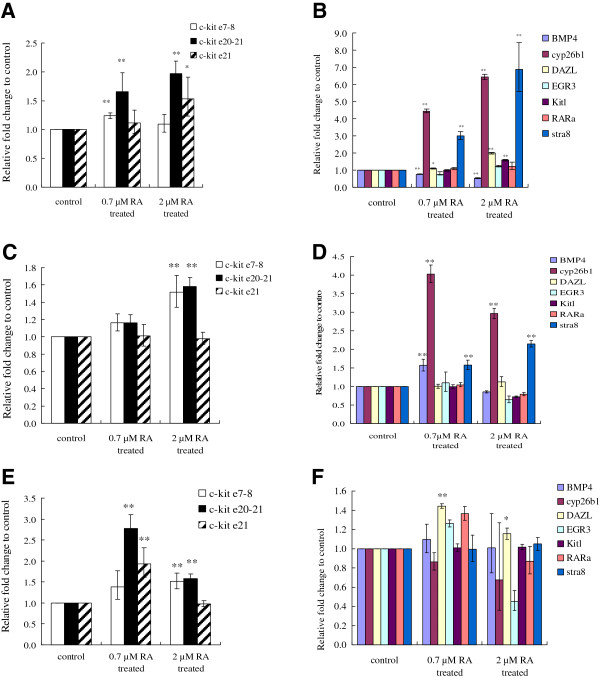
**Immunofluorescence study of Kit protein expression in 5 dpp, 10 dpp and 60 dpp mouse testes.** Images were obtained with ZEISS Axioplan 2 imaging system and Spot 4.7/SpotAdvanced software (Magnification × 400). Kit positive cells were stained with green or red fluorescein (Arrow). Leydig cells (Asterisk) displayed a large prominent nucleus. The cell nuclei was counterstained with 4′,6-diamidino-2-phenylindole. Images were merged with Photoshop CS4. **(A)** FITC labeled anti-Kit extracellular domain in 5 dpp mouse testes. White arrow: Kit positive cells **(B)** FITC labeled anti-Kit extracellular domain in 10 dpp mouse testes. **(C)** FITC labeled anti-Kit extracellular domain in 60 dpp mouse testes. **(D)** Alexa Fluo 594 labeled anti-Kit intracellular domain in 5 dpp mouse testes. **(E)** Alexa Fluo 594 labeled anti-Kit intracellular domain in 10 dpp mouse testes. **(F)** Alexa Fluo 594 labeled anti-Kit intracellular domain in 60 dpp mouse testes.

It was shown that full-length Kit (145 KDa) was expressed in CRL-2053 cells and testes except in c18-4 cells (Figure 6A). With an anti-intracellular antibody, a 50 KDa Kit was expressed in the c18-4, CRL-2053 and all testes (Figure 6B). From 5 dpp to 60 dpp, the 145 KDa Kit increased, but the 50 KDa Kit remained homogenous in the testes (Figure 6). Both the c18-4 and CRL-2053 cells expressed the intracellular domain of Kit (nuclear region) (Figure 7B and D). Unlike the CRL-2053 cells, the c18-4 cells did not express the extracelluar domain (membrane region) (Figure 7A). In the 5 ddp testes, expression of the extracellular domain was very minimal in the germ cells (Figure 8A). In the 10 dpp testes, a portion of the germ cells adjacent to the basement membrane of the seminiferous tubules began to express the membrane domain (Figure 8B). In the 60 dpp testes, some spermatogonia and spermatocytes were tested positive for membrane domain (Figure 8C). In contrast, the nuclear domain was expressed in all stages (5 dpp, 10 dpp and 60 dpp) in the spermatogonia, spermatocyte and spermatids, but not in the mature spermatozoon (Figure 8D, E and F). Leydig cells highly expressed the nuclear domain of Kit in the 60 dpp testes (Figure 8F).

### Expression changes of *c-kit* and other differentiation-related genes in the testes after RA stimulation

**Figure 9:**
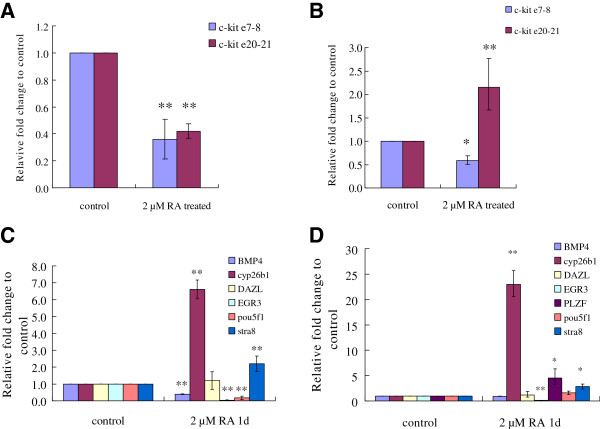
**Expression of *c-kit *and other germ cell differentiation-related genes in the 5 dpp, 10 dpp and 60 dpp mouse testes stimulated by RA.** 5 dpp, 10 dpp and 60 dpp testes were treated with 2 μM RA diluted in ethanol for 24 h *in vitro*. Testes treated with the same concentration of ethanol without RA were the control group. Realtime PCR was carried out for quantitative determination of the expression of *c-kit* and its potential regulatory genes (BMP4, Cyp26b1, DAZL, EGR3, Kitl, RARα and Stra8). Values of the vertical axis represented the expression fold change comparing with the control group. The results were normalized to GAPDH values. Error bars represent the S.E.M. Values with ** represented a significance with a P<0.01 whereas values with * represented a significance with a P<0.05 comparing with the control group. **(A)***c-kit* expression in the 5 dpp testes. **(B)** Expression of germ cell differentiation-related genes in the 5 dpp testes. **(C)***c-kit* expression in the 10 dpp testes. **(D)** Expression of germ cell differentiation-related genes in the 10 dpp testes. **(E)***c-kit* expression in the 60 dpp testes. **(F)** Expression of germ cell differentiation-related genes in the 60 dpp testes.

5 dpp, 10 dpp and 60 dpp mouse testes were treated with either 0.7 μM or 2 μM RA *in vitro* for 24 h. Quantitative expression of *c-kit* and SSCs differentiation related genes were determined by Real-time PCR using three pairs of primers (e7-8, e20-21 and e21^+^ as indicated by arrow heads in Figure 3). The total *c-kit* mRNA level increased following the RA treatment and exhibited a concentration-dependent pattern in 5 dpp and 10 dpp testes (Figure 9A, C). After RA stimulation, the 60 dpp testes did not display concentration-dependent increases any more (Figure 9E). In 5 dpp and 10 dpp testes, expressions of *Cyp26b1* and *Stra8* were significantly up-regulated (Figure 9B, D). Expressions of *Dazl* and *Kitl* were enhanced moderately in 5 dpp and 60 dpp testes (Figure 9B, F). *Bmp4* had a different response to RA treatment in testes of different ages (Figure 9B, D, F).The effects of RA on the expressions of *Bmp4, Cyp26b1* and *Stra8* were more significant in the 5 dpp testes than that in the 10 dpp testes. Expressions of RARα and *Egr3* were not altered in either stage (Figure 9B, D, F).

### Expression dynamics of *c-kit* and differentiation-related genes in germ cell lines after RA stimulation

**Figure 10:**
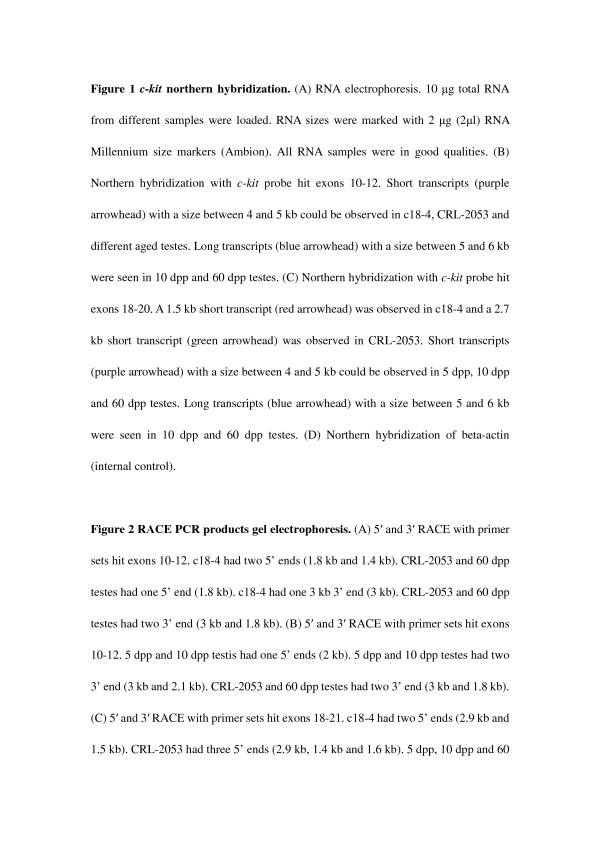
**Expression of *c-kit *and other germ cell differentiation-related genes in the c18-4 and CRL-2053 cells stimulated by RA.** The c18-4 and CRL-2053 cells were treated with 2 μM RA diluted in ethanol for 24 h *in vitro*. Cells treated with the same concentration of ethanol without RA were the control group. Realtime PCR was carried out for quantitative determination of the expression of *c-kit* and its potential regulatory genes *(Bmp4*, *Cyp26b1*, *Dazl*, *Egr3*, *Pou5f1* and *Stra8*). Values of the vertical axis represented the expression fold change comparing with the control group. The results were normalized to GAPDH values. Error bars represent the S.E.M. Values with ** represented a significance with a P<0.01 whereas values with * represented a significance with a P<0.05 comparing with the control group. **(A)***c-kit* expression in the c-18-4 cells. **(B)***c-kit* expression in the CRL-2053 cells. **(C)** Expression of germ cell differentiation-related genes in the c18-4 cells. **(D)** Expression of germ cell differentiation-related genes in the CRL-2053 cells.

*c-kit* and other differentiation-related genes changes were analyzed in C18-4 and CRL-2053 cells after RA treatment (2 μM RA for 24 hours). Unlike in the testes, the expressions of all transcripts declined in the c18-4 cells after RA stimulation (Figure 10A). In CRL-2053 cells, the amount of the long transcripts (e7-8 in Figure 10B) declined to about 50% but the quantity of total transcripts (e20-21 in Figure 10B) escalated to approximately 2 folds, indicating that the short transcripts must have increased even more significantly (Figure 10B). Expressions of *Cyp26b1* and *Stra8* increased significantly in both c18-4 and CRL-2053 cells (P < 0.01) (Figure 10C, 10D). The two germ cell marker genes, *Dazl* and *Pou5f1* had distinctive responses to RA. *Dazl* was boosted in 5 dpp and 60 dpp testis (Figure 9B, F), but not in either cell lines (Figure 10C, D). *Pou5f1* diminished in c18-4 cells but did not change in CRL-2053 cells (Figure 10C and D). *Egr3* (an early growth response gene) was suppressed in c18-4 and CRL-2053 cells (Figure 10C, 10D), but it was not altered in the testes (Figure 9B, D, F). *Rarα* (an RA receptor gene) did not respond to RA stimulation.

## Discussion

### A*. c-kit* transcripts during spermatogenesis

In addition of the conventional full-length *c-kit* and Tr-kit discovered, we also found more than 3*c-kit* mRNA transcripts in the SSCs and spermatogonia. CRL-2053 had the highest amount of transcript A (Figure 5A). Though its quantity increased, the percentages of Transcript A declined in CRL-2196 and 60 dpp testes when compared with that in c18-4 cells and testes at 5 dpp (Figure 4A, B, Figure 5A, B). The 145 KDa Kit was also absent in c18-4 cells (Figure 6A). On this basis, acquisition of this transcript marked the start of the transition from SSCs to spermatogonia. The percentage of this transcript decreased in CRL-2196 cells and 60 dpp testes. This was caused by the emergence of new short transcripts, which were important for later stage spermatogenesis.

Expression of transcript B was the highest in the 10 dpp testis (Figure 4A). The 3.9 kb short 3′ UTR transcript was composed of 21 exons, identical to the full-length transcript. The only difference was that the 3.9 kb short 3′ UTR transcript had a 1.2 kb shorter 3′ UTR than the full-length transcript. Combining its abundance in the testes (95.9% ~ 99.4%, Figure 4C) with the strong positive staining of Leydig cells in the 60 dpp testes (Figure 8F), it could be inferred that this transcript might be a somatic form. Functions of 3′ UTR included supplying binding sites for microRNAs and post-transcriptional regulation. Absence of the 3′ end UTR in all transcripts in the immortal germ cells (Figure 5A) indicated that the 3′ UTR modification was lost during immortalization and it might be controlled by testicular somatic factors.

Transcript C, encoding the 50 KDa Kit, was stably expressed in the testes (Figure 6B). The percentage of the short transcripts (Transcript C + D) was the highest in the 60 dpp testes (54.91%) (Figure 4B) and CRL-2196 (43.6%) (Figure 5B). Consequently, these transcripts might have significant roles in later stage spermatogenesis beginning from spermatocytes. Multiple sequence alignment of *c-kit* tranascripts was shown in Additional file 1.

### B. Kit profile during spermatogenesis

Two forms of Kit were discovered in this study: the 145 kDa and 50 kDa Kit. The 145 kDa Kit was located in the cytoplasm/membrane domain in CRL-2053 cells (Figure 7C) but not in c18-4 cells. Its expression escalated accordingly in the 5 dpp, 10 dpp, 15 dpp, 40 dpp and 60 dpp testes (Figure 6A). Unlike the 145 kDa full-length Kit, the 50 kDa Kit, possibly the product of transcript C, was expressed in both nuclear and cytoplasm/membrane domains in CRL-2053 cells (Figure 6). The 50 kDa Kit was stably expressed in the testes (Figure 6B). Therefore, the 145 kDa was indeed the marker for spermatogonia [43]. We highlighted here that its location shifted from the nucleus to the cytoplasm and then to the membrane domain. This might be vital for the initiation of spermatogenesis in SSCs. Expression of the extracellular full-length Kit on membranes, not in the nucleus or in the cytoplasm, endorsed the cells the ability to correspond with Kitl signals. Hence, this expression played important roles in differentiation initiation. We also demonstrated here that SSCs did not have full-length transcript (transcript A), nor the full-length Kit (145 kDa), which agreed with other studies indicating that the activation of the Kit/Kitl signaling pathway was not required for SSCs′ self-renewal [23,24]. Kit was initially expressed in the nucleus, and then ventured out to the cytoplasm and then to the membrane domain when SSCs became spermatogonia. ORF finder comparison of the c-kit putative proteins sequences were shown in Additional file 2.

### C. RA responses in germ cells and testes

Some studies showed that RA directly acts on spermatogenic cells by stimulating *Stra8* and *c-kit* gene expression, whereas some studies testified that exogenous RA could not stimulate *c-kit* expression [16,42,44]. We demonstrated that RA enhanced *c-kit* expression (Figure 9A, C and E) in testes. *Stra8* (gene stimulated by RA) was also significantly amplified in both cell lines and 5 dpp/10 dpp testes (Figure 9B, D). However, the expression pattern of *c-kit* in the cell lines was different from that in the testes (Figure 10*vs* Figure 9). Both long and short transcripts were reduced in c18-4 cells after RA stimulation (Figure 10A). On the other hand, the long transcripts were reduced whereas the short transcripts were promoted in CRL-2053 cells (Figure 10B). We concluded that RA indirectly impacted upon *c-kit* expression in male germ cells, while some unknown factors from the testes somatic cells might be involved. It agreed with previous works that RA indirectly controlled the timing of meiosis by juxtacrine of Sertoli cells [45].

Suppression of *Bmp4* by RA was obvious. RA reduced *Bmp*4 (a SSCs pluripotential maintenance gene) expression in 5 dpp testes (Figure 9B) and did not alter *Bmp4* expression in 10 dpp and 60 dpp testes (Figure 9D, F). *BMP4* was also reduced in c18-4 cells after RA stimulation (Figure 10C). Excessive exogenous RA would push SSCs into abnormal differentiation and finally apoptosis [16]. Our results validified that after 24 hours of 2 μM RA treatment, expression of *Cyp26b1* (a RA degradation gene) was stimulated. This would degrade excessive RA into the inactive form in both cell lines and testes (Figure 9B, C, Figure 10C, D). *Cyp26b1* was not altered in 60 dpp testes (Figure 9F). The increase of the protective gene also varied in CRL-2053 cells (~20 folds increase of *Cyp26b1*) and in c18-4 cells (~6 folds increase of *Cyp26b1*). We confirmed here, again, that *Stra8* was the most immediate responsive gene after RA stimulation. The germ cell marker genes (*Dazl* and *Pou5f1*), early growth response gene (*Egr3*), and the RA receptor gene (*Rarα*) did not respond to RA, especially not when RA was added to testes tissue culture.

## Conclusions

There are dynamic transcription and translation changes of *c-kit* before and after SSCs’ anticipated differentiation. These changes differ between in the cell lines and in the testis. The responses to RA stimulation are different between the cell lines and testis too. As a significant upstream regulatory factor for *c-kit* expression, RA might play with other unknown factors to precisely regulate the expression profiles of *c-kit* in order to regulate normal spermatogenesis.

## Competing interests

The authors declare that they have no competing interests.

## Authors’ contributions

LZ and YH carried out the molecular genetic studies, participated in the RACE, Northern blot, sequence alignment and drafted the manuscript. JT carried out the immunoassays. CH and HF participated in the design of the study. LL participated online mRNA and protein prediction analysis. XT performed the statistical analysis. All authors read and approved the final manuscript.

## Supplementary Material

Additional file 1 **Multiple sequence alignment of *c-kit* transcripts.** Click here for file

Additional file 2 **Multiple sequence alignment of ORF finder predicted *c-kit *** **proteins.** Click here for file
