# Supplementary material for: c-kit expression profile and regulatory factors during spermatogonial stem cell differentiation
Source: BMC Dev Biol. 2013 Oct 27;13:38. doi: 10.1186/1471-213X-13-38 (PMC3871025; doi:10.1186/1471-213X-13-38)
Supplement: Additional file 1 — Multiple sequence alignment of c-kit transcripts. [file 1471-213X-13-38-S1.doc]

**Additioanl file 1 Multiple sequence alignment of *c-kit* transcripts**

NM_001122733.1 ----------------------------GCTCGGTGCACTTGGGCGAGAGCTGTAGCAGA 32

NM_021099.3 --------------------------------GGTGCACTTGGGCGAGAGCTGTAGCAGA 28

X65997.1 ------------------------------------------------------------

Full_length ----------------------------------GGCACTTGGGCGAGAGCTGTAGCAGA 26

Short_3_end_UTR ----------------------------------GGCACTTGGGCGAGAGCTGTAGCAGA 26

SSCs_specific ------------------------------------------------------------

Tr-kit_c18-4_2.7kb ------------------------------------------------------------

Tr-kit_c18-4_2.9kb ------------------------------------------------------------

Tr-kit_c18-4_4.0kb ------------------------------------------------------------

Tr-kit_CRL2053_1.9kb ------------------------------------------------------------

Tr-kit_CRL2053_2.7kb GAGTGGCTCTGGGGCTCGGCTTTGCCGCGCTCGGTGCACTTGGGCGAGAGCTGTAGCAGA 60

Tr-kit_CRL2053_3.1kb ------------------------------------------------------------

Tr-kit_CRL2053_3.9kb ------------------------------------------------------------

NM_001122733.1 GAGAGGAGCTCAGAGTCTAGCGCAGCCACCGCGATGAGAGGCGCTCGCGGCGCCTGGGAT 92

NM_021099.3 GAGAGGAGCTCAGAGTCTAGCGCAGCCACCGCGATGAGAGGCGCTCGCGGCGCCTGGGAT 88

X65997.1 ------------------------------------------------------------

Full_length GAGAGGAGCTCAGAGTCTAGCGCAGCCACCGCGATGAGAGGCGCTCGCGGCGCCTGGGAT 86

Short_3_end_UTR GAGAGGAGCTCAGAGTCTAGCGCAGCCACCGCGATGAGAGGCGCTCGCGGCGCCTGGGAT 86

SSCs_specific ------------------------------------------------------------

Tr-kit_c18-4_2.7kb ------------------------------------------------------------

Tr-kit_c18-4_2.9kb ------------------------------------------------------------

Tr-kit_c18-4_4.0kb ------------------------------------------------------------

Tr-kit_CRL2053_1.9kb ------------------------------------------------------------

Tr-kit_CRL2053_2.7kb GAGAGGAGCTCAGAGTCTAGCGCAGCCACCGCGATGAGAGGCGCTCGCGGCGCCTGGGAT 120

Tr-kit_CRL2053_3.1kb ------------------------------------------------------------

Tr-kit_CRL2053_3.9kb ------------------------------------------------------------

NM_001122733.1 CTGCTCTGCGTCCTGTTGGTCCTGCTCCGTGGCCAGACAGCCACGTCTCAGCCATCTGCA 152

NM_021099.3 CTGCTCTGCGTCCTGTTGGTCCTGCTCCGTGGCCAGACAGCCACGTCTCAGCCATCTGCA 148

X65997.1 ------------------------------------------------------------

Full_length CTGCTCTGCGTCCTGTTGGTCCTGCTCCGTGGCCAGACAGCCACGTCTCAGCCATCTGCA 146

Short_3_end_UTR CTGCTCTGCGTCCTGTTGGTCCTGCTCCGTGGCCAGACAGCCACGTCTCAGCCATCTGCA 146

SSCs_specific -----------------------------------------TCTAATACGACTCACTATA 19

Tr-kit_c18-4_2.7kb ------------------------------------------------------------

Tr-kit_c18-4_2.9kb ------------------------------------------------------------

Tr-kit_c18-4_4.0kb ------------------------------------------------------------

Tr-kit_CRL2053_1.9kb ------------------------------------------------------------

Tr-kit_CRL2053_2.7kb CTGCTCTGCGTCCTGTTGGTCCTGCTCCGTGGCCAGACAGCCACGTCTCAGCCATCTGCA 180

Tr-kit_CRL2053_3.1kb ------------------------------------------------------------

Tr-kit_CRL2053_3.9kb ------------------------------------------------------------

NM_001122733.1 AGTCCAGGGGAGCCGTCTCCGCCATCCATCCATCCAGCACAATCAGAGTTAATAGTTGAA 212

NM_021099.3 AGTCCAGGGGAGCCGTCTCCGCCATCCATCCATCCAGCACAATCAGAGTTAATAGTTGAA 208

X65997.1 ------------------------------------------------------------

Full_length AGTCCAGGGGAGCCGTCTCCGCCATCCATCCATCCAGCACAATCAGAGTTAATAGTTGAA 206

Short_3_end_UTR AGTCCAGGGGAGCCGTCTCCGCCATCCATCCATCCAGCACAATCAGAGTTAATAGTTGAA 206

SSCs_specific GGGCAAGCAGTGGTATCAACGCAGAGTACATGGGCAGTATAATTAGGACACGTTGGAAAT 79

Tr-kit_c18-4_2.7kb ------------------------------------------------------------

Tr-kit_c18-4_2.9kb ------------------------------------------------------------

Tr-kit_c18-4_4.0kb ------------------------------------------------------------

Tr-kit_CRL2053_1.9kb -----GGGAGGGCC---------------------------------------------- 9

Tr-kit_CRL2053_2.7kb AGTCCAGGGGAGCCGTCTCCGCCATCCATCCATCCAGCACAATCAGAGTTAATAGTTGAA 240

Tr-kit_CRL2053_3.1kb ------------------------------------------------------------

Tr-kit_CRL2053_3.9kb ------------------------------------------------------------

NM_001122733.1 GCTGGCGACACCCTCAGCCTGACGTGCATTGATCCCGACTTTGTCAGATGGACTTTCAAG 272

NM_021099.3 GCTGGCGACACCCTCAGCCTGACGTGCATTGATCCCGACTTTGTCAGATGGACTTTCAAG 268

X65997.1 ------------------------------------------------------------

Full_length GCTGGCGACACCCTCAGCCTGACGTGCATTGATCCCGACTTTGTCAGATGGACTTTCAAG 266

Short_3_end_UTR GCTGGCGACACCCTCAGCCTGACGTGCATTGATCCCGACTTTGTCAGATGGACTTTCAAG 266

SSCs_specific GTTA-------------------------------------------------------- 83

Tr-kit_c18-4_2.7kb ------------------------------------------------------------

Tr-kit_c18-4_2.9kb ------------------------------------------------------------

Tr-kit_c18-4_4.0kb ------------------------------------------------------------

Tr-kit_CRL2053_1.9kb --------CACCCT----------------GGTC-------------------------- 19

Tr-kit_CRL2053_2.7kb GCTGGCGACACCCTCAGCCTGACGTGCATTGATCCCGACTTTGTCAGATGGACTTTCAAG 300

Tr-kit_CRL2053_3.1kb ------------------------------------------------------------

Tr-kit_CRL2053_3.9kb ------------------------------------------------------------

NM_001122733.1 ACCTATTTCAATGAAATGGTTGAGAATAAAAAAAATGAATGGATCCAGGAAAAAGCCGAG 332

NM_021099.3 ACCTATTTCAATGAAATGGTTGAGAATAAAAAAAATGAATGGATCCAGGAAAAAGCCGAG 328

X65997.1 ------------------------------------------------------------

Full_length ACCTATTTCAATGAAATGGTTGAGAATAAAAAAAATGAATGGATCCAGGAAAAAGCCGAG 326

Short_3_end_UTR ACCTATTTCAATGAAATGGTTGAGAATAAAAAAAATGAATGGATCCAGGAAAAAGCCGAG 326

SSCs_specific ----AATTCAATTTAAAATTTCAGTTAAAATGAGTTACAT---TTTGCTAAACATCTGTA 136

Tr-kit_c18-4_2.7kb ------------------------------------------------------------

Tr-kit_c18-4_2.9kb ------------------------------------------------------------

Tr-kit_c18-4_4.0kb ---------------------------AAAGGGACTGCG--------------------- 12

Tr-kit_CRL2053_1.9kb ----ATTACA-------------GAATA-------------------------------- 30

Tr-kit_CRL2053_2.7kb ACCTATTTCAATGAAATGGTTGAGAATAAAAAAAATGAATGGATCCAGGAAAAAGCCGAG 360

Tr-kit_CRL2053_3.1kb ------------------------------------------------------------

Tr-kit_CRL2053_3.9kb ----------------------------GAGTGGCT------------------------ 8

NM_001122733.1 GCCACTCGCACGGGCACATACACGTGCAGCAACAGCAATGGCCTCACGAGTTCTATTTAC 392

NM_021099.3 GCCACTCGCACGGGCACATACACGTGCAGCAACAGCAATGGCCTCACGAGTTCTATTTAC 388

X65997.1 ------------------------------------------------------------

Full_length GCCACTCGCACGGGCACATACACGTGCAGCAACAGCAATGGCCTCACGAGTTCTATTTAC 386

Short_3_end_UTR GCCACTCGCACGGGCACATACACGTGCAGCAACAGCAATGGCCTCACGAGTTCTATTTAC 386

SSCs_specific TCGAAATGTTAACAGTATCTTTTATGTGATCTTGTCAGCAA------ATGTTTTCTTTTC 190

Tr-kit_c18-4_2.7kb ------------------------------------------------------------

Tr-kit_c18-4_2.9kb ------------------------------------------------------------

Tr-kit_c18-4_4.0kb ------------------------------------AATGG------AAGTTTTCTTTTG 30

Tr-kit_CRL2053_1.9kb ------------------------------------------------------------

Tr-kit_CRL2053_2.7kb GCCACTCGCACGGGCACATACACGTGCAGCAACAGCAATGGCCTCACGAGTTCTATTTAC 420

Tr-kit_CRL2053_3.1kb ------------------------------------------------------------

Tr-kit_CRL2053_3.9kb --------------------------------------CTG------GGGCTCGGCTTTG 24

NM_001122733.1 GTGTTTGTTAGAGATCCTGCCAAACTTTTCCTGGTTGGCCTTCCCTTGTTTGGCAAAGAA 452

NM_021099.3 GTGTTTGTTAGAGATCCTGCCAAACTTTTCCTGGTTGGCCTTCCCTTGTTTGGCAAAGAA 448

X65997.1 --ATTC------------------------------------------------------ 4

Full_length GTGTTTGTTAGAGATCCTGCCAAACTTTTCCTGGTTGGCCTTCCCTTGTTTGGCAAAGAA 446

Short_3_end_UTR GTGTTTGTTAGAGATCCTGCCAAACTTTTCCTGGTTGGCCTTCCCTTGTTTGGCAAAGAA 446

SSCs_specific CTGTTCTATATGTATGTGTATGTATGTATGTATGTATGTAAGTGCTTACCCATCTGCACT 250

Tr-kit_c18-4_2.7kb ------------------------------------------------------------

Tr-kit_c18-4_2.9kb ------------------------------------------------------------

Tr-kit_c18-4_4.0kb TAATTC------------------------------------------------------ 36

Tr-kit_CRL2053_1.9kb ----TTGTT---------GCTA---------TGGT-GATCTT---TTG------------ 52

Tr-kit_CRL2053_2.7kb GTGTTTGTTAGAGATCCTGCCAAACTTTTCCTGGTTGGCCTTCCCTTGTTTGGCAAAGAA 480

Tr-kit_CRL2053_3.1kb ------------------------------------------------------------

Tr-kit_CRL2053_3.9kb CCGCGCT----------------------------------------------------- 31

NM_001122733.1 GACAGCGACGCGCTG-GTCCGCTGCCCTCTGACAGACCCACAGGTGTCCAATTATTCCCT 511

NM_021099.3 GACAGCGACGCGCTG-GTCCGCTGCCCTCTGACAGACCCACAGGTGTCCAATTATTCCCT 507

X65997.1 ----------------------------CCA-------GGTAACTA-------------- 15

Full_length GACAGCGACGCGCTG-GTCCGCTGCCCTCTGACAGACCCACAGGTGTCCAATTATTCCCT 505

Short_3_end_UTR GACAGCGACGCGCTG-GTCCGCTGCCCTCTGACAGACCCACAGGTGTCCAATTATTCCCT 505

SSCs_specific GTCAGAATGGTACAGAGCCCACTGCAGGCTG-------TGTAGATAGCCTCTATGTCTAG 303

Tr-kit_c18-4_2.7kb ------------------------------------------------------------

Tr-kit_c18-4_2.9kb ------------------------------------------------------------

Tr-kit_c18-4_4.0kb ----------------------------CT---------GTAAATA-------------- 45

Tr-kit_CRL2053_1.9kb -------------------------------------------------AATTTTT---- 59

Tr-kit_CRL2053_2.7kb GACAGCGACGCGCTG-GTCCGCTGCCCTCTGACAGACCCACAGGTGTCCAATTATTCCCT 539

Tr-kit_CRL2053_3.1kb ------------------------------------------------------------

Tr-kit_CRL2053_3.9kb ----------------------------CGG-------TGCACTTGGGCGAGAGCTGTAG 56

NM_001122733.1 CATCGAGTGTGATGGGAAATCTCTCCCCACGGACCTGACGTTTGTCCCAAACCCCAAGGC 571

NM_021099.3 CATCGAGTGTGATGGGAAATCTCTCCCCACGGACCTGACGTTTGTCCCAAACCCCAAGGC 567

X65997.1 C------CAAGCTAAAA-------------------------------GACA-----GGC 33

Full_length CATCGAGTGTGATGGGAAATCTCTCCCCACGGACCTGACGTTTGTCCCAAACCCCAAGGC 565

Short_3_end_UTR CATCGAGTGTGATGGGAAATCTCTCCCCACGGACCTGACGTTTGTCCCAAACCCCAAGGC 565

SSCs_specific C------TGTGACTAGGCTTCTTGTCTAGCGA--------------CTGAGT-----GAC 338

Tr-kit_c18-4_2.7kb ------------------------------------------------------------

Tr-kit_c18-4_2.9kb ---------------------------------------------------------GCC 3

Tr-kit_c18-4_4.0kb C------TGCGATGGGACGTG---------------------------AAGT-----GTC 67

Tr-kit_CRL2053_1.9kb ------------TGAGAAG----------------------------------------- 66

Tr-kit_CRL2053_2.7kb CATCGAGTGTGATGGGAAATCTCTCCCCACGGACCTGACGTTTGTCCCAAACCCCAAGGC 599

Tr-kit_CRL2053_3.1kb ------------------------------------------------------------

Tr-kit_CRL2053_3.9kb C------AGAGAGAGGAGCTC----------------------------AGA-----GTC 77

NM_001122733.1 TGGCATCACCATCAAAAACGTGAAGCGCGCCTACCACCGGCTCTGTGTCCGCTGTGCTGC 631

NM_021099.3 TGGCATCACCATCAAAAACGTGAAGCGCGCCTACCACCGGCTCTGTGTCCGCTGTGCTGC 627

X65997.1 TG------AAA---------TGAAA---------------------G------------- 44

Full_length TGGCATCACCATCAAAAACGTGAAGCGCGCCTACCACCCGCTCTGTGTCCGCTGTGCTGC 625

Short_3_end_UTR TGGCATCACCATCAAAAACGTGAAGCGCGCCTACCACCGGCTCTGTGTCCGCTGTGCTGC 625

SSCs_specific TAGCAGACCTT---------TGAAG---------------------GCATTGTGGTACAC 368

Tr-kit_c18-4_2.7kb ------------------------------------------------------------

Tr-kit_c18-4_2.9kb TG---------------------------------------------------------- 5

Tr-kit_c18-4_4.0kb TG------CTG---------TGAAG---------------------G------------- 78

Tr-kit_CRL2053_1.9kb ---------------------GAAGCGTGACT-----------------CGTT------- 81

Tr-kit_CRL2053_2.7kb TGGCATCACCATCAAAAACGTGAAGCGCGCCTACCACCGGCTCTGTGTCCGCTGTGCTGC 659

Tr-kit_CRL2053_3.1kb ------------------------------------------------------------

Tr-kit_CRL2053_3.9kb TAGCGCAGCCACCGCGA---TGAGA---------------------G------------- 100

NM_001122733.1 TCAGCGTGACGGTACATGGCTGCATTCTGACAAATTCACCCTCAAAGTGCGGGCAGCCAT 691

NM_021099.3 TCAGCGTGACGGTACATGGCTGCATTCTGACAAATTCACCCTCAAAGTGCGGGCAGCCAT 687

X65997.1 -------------AC--------------------CC-TCTT-------C---------- 53

Full_length TCAGCGTGACGGTACATGGCTGCATTCTGACAAATTCACCCTCAAAGTGCGGGCAGCCAT 685

Short_3_end_UTR TCAGCGTGACGGTACATGGCTGCATTCTGACAAATTCACCCTCAAAGTGCGGGCAGCCAT 685

SSCs_specific CTGAGATAAGGAAACAGGGC---------ACTTGAGG-TTTTTTA-GTGCC--------- 408

Tr-kit_c18-4_2.7kb ------------------------------------------------------------

Tr-kit_c18-4_2.9kb ----------------------------------GCG-TTTCCTA--------------- 15

Tr-kit_c18-4_4.0kb -------------ACAAGCC---------ACAGAGCC-TCTTTTA-CTGC---------- 104

Tr-kit_CRL2053_1.9kb ----------------------TATTTT------------CTCAAAG------------- 94

Tr-kit_CRL2053_2.7kb TCAGCGTGACGGTACATGGCTGCATTCTGACAAATTCACCCTCAAAGTGCGGGCAGCCAT 719

Tr-kit_CRL2053_3.1kb ------------------------------------------------------------

Tr-kit_CRL2053_3.9kb -------------GCG---C---------TCGCGGCG-CCTGGGATCTGC---------- 124

NM_001122733.1 CAAGGCTATCCCTGTTGTGTCTGTGCCTGAAACAAGTCACCTCCTTAAGAAAGGGGACAC 751

NM_021099.3 CAAGGCTATCCCTGTTGTGTCTGTGCCTGAAACAAGTCACCTCCTTAAGAAAGGGGACAC 747

X65997.1 --------CTTGTGT--------------------------------------------- 60

Full_length CAAGGCTATCCCTGTTGTGTCTGTGCCTGAAACAAGTCACCTCCTTAAGAAAGGGGACAC 745

Short_3_end_UTR CAAGGCTATCCCTGTTGTGTCTGTGCCTGAAACAAGTCACCTCCTTAAGAAAGGGGACAC 745

SSCs_specific -----CAGTTTCTGTGGTGTTTA-----------------------------------GC 428

Tr-kit_c18-4_2.7kb ------------------------------------------------------------

Tr-kit_c18-4_2.9kb ------------------------------------------------------------

Tr-kit_c18-4_4.0kb --------TTTGTCTAGTTTTAG-----------------------------------AC 121

Tr-kit_CRL2053_1.9kb CAAG-------------------------------------------------------- 98

Tr-kit_CRL2053_2.7kb CAAGGCTATCCCTGTTGTGTCTGTGCCTGAAACAAGTCACCTCCTTAAGAAAGGGGACAC 779

Tr-kit_CRL2053_3.1kb ------------------------------------------------------------

Tr-kit_CRL2053_3.9kb --------TCTGCGTCCTGTTGG-----------------------------------TC 141

NM_001122733.1 ATTTACGGTGGTGTGCACCATAAAAGATGTGTCTACATCCGTGAACTCCATGTGGCTAAA 811

NM_021099.3 ATTTACGGTGGTGTGCACCATAAAAGATGTGTCTACATCCGTGAACTCCATGTGGCTAAA 807

X65997.1 -----CCTTGGG----------AGA-A--------------------------------- 71

Full_length ATTTACGGTGGTGTGCACCATAAAAGATGTGTCTACATCCGTGAACTCCATGTGGCTAAA 805

Short_3_end_UTR ATTTACGGTGGTGTGCACCATAAAAGATGTGTCTACATCCGTGAACTCCATGTGGCTAAA 805

SSCs_specific ATTGGCCATAGGACTCATGCCAAAA-A---GCTGTATATCCTCAGTTTCT---------G 475

Tr-kit_c18-4_2.7kb ------------------------------------------------------------

Tr-kit_c18-4_2.9kb ------CGTGG------------------------------------------------- 20

Tr-kit_c18-4_4.0kb A---ACCCTAGATTTCA--CTTAGA-A---GTT----------AATTTTT---------A 153

Tr-kit_CRL2053_1.9kb ------------------------------------------------------------

Tr-kit_CRL2053_2.7kb ATTTACGGTGGTGTGCACCATAAAAGATGTGTCTACATCCGTGAACTCCATGTGGCTAAA 839

Tr-kit_CRL2053_3.1kb ------------------------------------------------------------

Tr-kit_CRL2053_3.9kb CTGCTCCGTGG---------CCAGACA---GCC--------------------------A 163

NM_001122733.1 GATGAACCCTCAGCCTCAGCACATAGCCCAGGTAAAGCACAATAGCTGGCACCGGGGTGA 871

NM_021099.3 GATGAACCCTCAGCCTCAGCACATAGCCCAGGTAAAGCACAATAGCTGGCACCGGGGTGA 867

X65997.1 GAC----------------------GTCAAG----------------------------- 80

Full_length GATGAACCCTCAGCCTCAGCACATAGCCCAGGTAAAGCACAATAGCTGGCACCGGGGTGA 865

Short_3_end_UTR GATGAACCCTCAGCCTCAGCACATAGCCCAGGTAAAGCACAATAGCTGGCACCGGGGTGA 865

SSCs_specific GATGAGGAAGGGGACGCATCA--TAACTCTGC---------------------------- 505

Tr-kit_c18-4_2.7kb ------------------------------------------------------------

Tr-kit_c18-4_2.9kb ------------------------------------------------------------

Tr-kit_c18-4_4.0kb GAT----------------------ACCCAGG---------------------------- 163

Tr-kit_CRL2053_1.9kb ------------------------------------------------------------

Tr-kit_CRL2053_2.7kb GATGAACCCTCAGCCTCAGCACATAGCCCAG----------------------------- 870

Tr-kit_CRL2053_3.1kb ------------------------------------------------------------

Tr-kit_CRL2053_3.9kb CGT-----------------------CTCAGC---------------------------- 172

NM_001122733.1 CTTCAATTATGAACGCC-----------------AGGAGACGCTGACTATCAGCTCGGCA 914

NM_021099.3 CTTCAATTATGAACGCC-----------------AGGAGACGCTGACTATCAGCTCGGCA 910

X65997.1 ------TTG--AAGCT--------------------GTGAAACTTTT------------- 99

Full_length CTTCAATTATGAACGCC-----------------AGGAGACGCTGACTATCAGCTCGGCA 908

Short_3_end_UTR CTTCAATTATGAACGCC-----------------AGGAGACGCTGACTATCAGCTCGGCA 908

SSCs_specific TCTCTGTTGTTAATTTCCCCAGCCAGAGCAATGAAAAGGATGCTTCC------------- 552

Tr-kit_c18-4_2.7kb ------------------------------------------------------------

Tr-kit_c18-4_2.9kb -------TGCCAACC---------------------AAGACA------------------ 34

Tr-kit_c18-4_4.0kb CCCC--TTG--AATTC--------------------AAGACACTAAT------------- 186

Tr-kit_CRL2053_1.9kb ------------------------------------------------------------

Tr-kit_CRL2053_2.7kb ------------------------------------------------------------

Tr-kit_CRL2053_3.1kb ------------------------------------------------------------

Tr-kit_CRL2053_3.9kb CATC---TGCAAGTCC-------------------AGGGGAGCCG--------------- 195

NM_001122733.1 AGAGTTGACGATTCTGGAGTGTTCATGTGTTATGCCAATAATACTTTT----GGATCAGC 970

NM_021099.3 AGAGTTGACGATTCTGGAGTGTTCATGTGTTATGCCAATAATACTTTT----GGATCAGC 966

X65997.1 ------------TTT-------TTTTT-----------T-----TT----------TTTT 114

Full_length AGAGTTGACGATTCTGGAGTGTTCATGTGTTATGCCAATAATACTTTT----GGATCAGC 964

Short_3_end_UTR AGAGTTGACGATTCTGGAGTGTTCATGTGTTATGCCAATAATACTTTT----GGATCAGC 964

SSCs_specific --AAAATGTCTTTCT-------TCTCTGTGGAAGACACTTACGTTCATTGTAGAAACTTT 603

Tr-kit_c18-4_2.7kb ------------------------------------------------------------

Tr-kit_c18-4_2.9kb ------------------------------------------------------------

Tr-kit_c18-4_4.0kb --AGA-------TCC-------TCACT-----------TAACACTC----------CTGT 209

Tr-kit_CRL2053_1.9kb ------------------------------------------------------------

Tr-kit_CRL2053_2.7kb ------------------------------------------------------------

Tr-kit_CRL2053_3.1kb ------------------------------------------------------------

Tr-kit_CRL2053_3.9kb ------------TCT-------CCGCC---------------ATCCAT--------CCAT 213

NM_001122733.1 AAATGTCACAACAACCTTGAAAGTAGTAGAAAAAGGATTCATCAACATCTCCCCTGTGAA 1030

NM_021099.3 AAATGTCACAACAACCTTGAAAGTAGTAGAAAAAGGATTCATCAACATCTCCCCTGTGAA 1026

X65997.1 GGAGAAAAC------GTTCAAAG-------------------AGATGCATACA------A 143

Full_length AAATGTCACAACAACCTTGAAAGTAGTAGAAAAAGGATTCATCAACATCTCCCCTGTGAA 1024

Short_3_end_UTR AAATGTCACAACAACCTTGAAAGTAGTAGAAAAAGGATTCATCAACATCTCCCCTGTGAA 1024

SSCs_specific AGAAAACGTAAGAGAGCAGAAAGAAGCAAATGTAAATCACCCAGAGAGGCAGTCCATTCA 663

Tr-kit_c18-4_2.7kb ------------------------------------------------------------

Tr-kit_c18-4_2.9kb -GACAA-------------GAGG------------------------------------A 44

Tr-kit_c18-4_4.0kb GGAGAACGCA-----GCATAAAG--------------TCTGGGGATAGATAGT------A 244

Tr-kit_CRL2053_1.9kb ------------------------------------------------------------

Tr-kit_CRL2053_2.7kb ------------------------------------------------------------

Tr-kit_CRL2053_3.1kb ------------------------------------------------------------

Tr-kit_CRL2053_3.9kb CCAGCACAA--------TCAGAG----------------------TTAATAGTTGAAGCT 243

NM_001122733.1 GAACACTACAGTATTTGTAACCGATGGAGAAAACG----------TAGATTTGGTTGTTG 1080

NM_021099.3 GAACACTACAGTATTTGTAACCGATGGAGAAAACG----------TAGATTTGGTTGTTG 1076

X65997.1 AA----TG--AACTTT-----------------------------CAT-TTTAG-----A 162

Full_length GAACACTACAGTATTTGTAACCGATGGAGAAAACG----------TAGATTTGGTTGTTG 1074

Short_3_end_UTR GAACACTACAGTATTTGTAACCGATGGAGAAAACG----------TAGATTTGGTTGTTG 1074

SSCs_specific GA----TGTAGCTTTTCATTAAAAGCAGCATAACGTTAAGTGTGTTAC-TTCAG-----G 713

Tr-kit_c18-4_2.7kb ------------------------------------------------------------

Tr-kit_c18-4_2.9kb GA----T----------------------------------------C-CGCAA-----G 54

Tr-kit_c18-4_4.0kb GA----TG--GTTTTTA----------------------------TAG-TTTGA-----G 264

Tr-kit_CRL2053_1.9kb ------------------------------------------------------------

Tr-kit_CRL2053_2.7kb ------------------------------------------------------------

Tr-kit_CRL2053_3.1kb ------------------------------------------------------------

Tr-kit_CRL2053_3.9kb GG----CGACACCCTCAGCCTGACG--------------------TGC-ATTGA-----T 273

NM_001122733.1 AATACGAGGCCTACCCCAAACCCGAGCACCAGCAGTGGATATATATGAACAGGACCTCGG 1140

NM_021099.3 AATACGAGGCCTACCCCAAACCCGAGCACCAGCAGTGGATATATATGAACAGGACCTCGG 1136

X65997.1 AATG-------------------------------------------------------- 166

Full_length AATACGAGGCCTACCCCAAACCCGAGCACCAGCAGTGGATATATATGAACAGGACCTCGG 1134

Short_3_end_UTR AATACGAGGCCTACCCCAAACCCGAGCACCAGCAGTGGATATATATGAACAGGACCTCGG 1134

SSCs_specific AATGTCAGTTTCTC-TGTACTCTG-GTTTCA----------------------ACTTACA 749

Tr-kit_c18-4_2.7kb ------------------------------------------------------------

Tr-kit_c18-4_2.9kb AAT--------------------------------------------------------- 57

Tr-kit_c18-4_4.0kb AATCATCTCCTCCC-TCAACCCTCCACCCCC----------------------ACCCCCA 301

Tr-kit_CRL2053_1.9kb ------------------------------------------------------------

Tr-kit_CRL2053_2.7kb ------------------------------------------------------------

Tr-kit_CRL2053_3.1kb ------------------------------------------------------------

Tr-kit_CRL2053_3.9kb CCCGACTTTGTCAGATGGACTTTCAAGACCT----------------------ATTTCAA 311

NM_001122733.1 CTAACAAAGGGAAGGATTATGTCAAATCTGATAACAAAAGCAACATCAGATATGTGAACC 1200

NM_021099.3 CTAACAAAGGGAAGGATTATGTCAAATCTGATAACAAAAGCAACATCAGATATGTGAACC 1196

X65997.1 --------GGATTTGACTAT---------------------------------------- 178

Full_length CTAACAAAGGGAAGGATTATGTCAAATCTGATAACAAAAGCAACATCAGATATGTGAACC 1194

Short_3_end_UTR CTAACAAAGGGAAGGATTATGTCAAATCTGATAACAAAAGCAACATCAGATATGTGAACC 1194

SSCs_specific TCTTT---AAAGATGCCTGTGCAGCCAGTAAG--------------GATGTGTTTGTGGG 792

Tr-kit_c18-4_2.7kb ------------------------------------------------------------

Tr-kit_c18-4_2.9kb --------AGACTCG--TACA--------------------------------------- 68

Tr-kit_c18-4_4.0kb CCCCC---GGACACGACTGCAC------TAAA--------------CA------------ 326

Tr-kit_CRL2053_1.9kb ------------------------------------------------------------

Tr-kit_CRL2053_2.7kb ------------------------------------------------------------

Tr-kit_CRL2053_3.1kb ------------------------------------------------------------

Tr-kit_CRL2053_3.9kb T--------GAAATGGTTGAGAA-----TAAA--------------AAA----------- 333

NM_001122733.1 AACTTCGCCTGACCAGATTAAAAGGCACAGAAGGAGGCACTTATACCTTTCTGGTGTCCA 1260

NM_021099.3 AACTTCGCCTGACCAGATTAAAAGGCACAGAAGGAGGCACTTATACCTTTCTGGTGTCCA 1256

X65997.1 ------------TTATA-------A---------TG--CAT-----TTTCCT-------- 195

Full_length AACTTCGCCTGACCAGATTAAAAGGCACAGAAGGAGGCACTTATACCTTTCTGGTGTCCA 1254

Short_3_end_UTR AACTTCGCCTGACCAGATTAAAAGGCACAGAAGGAGGCACTTATACCTTTCTGGTGTCCA 1254

SSCs_specific TATTGTGCTCTGCCAGA-------ACACATGACCAGGTTCT-----AGTCCTAGCAC-CA 839

Tr-kit_c18-4_2.7kb ------------------------------------------------------------

Tr-kit_c18-4_2.9kb -------------TAGA-------A----------------------------------- 73

Tr-kit_c18-4_4.0kb -ACTTCAACC--CTAGA-------A---------GGGCTCT-----CTTCCTACTGTTCA 362

Tr-kit_CRL2053_1.9kb ------------------------------------------------------------

Tr-kit_CRL2053_2.7kb ------------------------------------------------------------

Tr-kit_CRL2053_3.1kb ------------------------------------------------------------

Tr-kit_CRL2053_3.9kb -AATGAATGGATCCAGG-------AAAAAGCCGAGGCCACT-----CGCACGGGCACATA 380

NM_001122733.1 ACTCTG-----ATGCCAGTGCTTCCGTGACATTCAACGTTTACGTGAACAC--------- 1306

NM_021099.3 ACTCTG-----ATGCCAGTGCTTCCGTGACATTCAACGTTTACGTGAACAC--------- 1302

X65997.1 ---GTG----------AATGGAAGGA---------------------------------- 208

Full_length ACTCTG-----ATGCCAGTGCTTCCGTGACATTCAACGTTTACGTGAACAC--------- 1300

Short_3_end_UTR ACTCTG-----ATGCCAGTGCTTCCGTGACATTCAACGTTTACGTGAACAC--------- 1300

SSCs_specific AGAATA----------AATGTAGGTAGGTAGGTAGGTAGGTAGGTAGGTAGGCAGGTAGC 889

Tr-kit_c18-4_2.7kb ------------------------------------------------------------

Tr-kit_c18-4_2.9kb ---AGA----------GACGT--------------------------------------- 81

Tr-kit_c18-4_4.0kb GTCGTA----------AACGGATTTA---------------------------------- 378

Tr-kit_CRL2053_1.9kb ------------------------------------------------------------

Tr-kit_CRL2053_2.7kb ------------------------------------------------------------

Tr-kit_CRL2053_3.1kb ------------------------------------------------------------

Tr-kit_CRL2053_3.9kb CACGTGCAGCAACAGCAATGGCCTCAC--------------------------------- 407

NM_001122733.1 ---------AAAACCAGAAATCCTGACG-----------TACGACAGGCTCATAAATGGC 1346

NM_021099.3 ---------AAAACCAGAAATCCTGACG-----------TACGACAGGCTCATAAATGGC 1342

X65997.1 -----------AGGGA--------------------------GAAAGACGTTTA------ 225

Full_length ---------AAAACCAGAAATCCTGACG-----------TACGACAGGCTCATAAATGGC 1340

Short_3_end_UTR ---------AAAACCAGAAATCCTGACG-----------TACGACAGGCTCATAAATGGC 1340

SSCs_specific TAGGGAGGGAGAGGGAGAGATGATGTATTTG---AATACCAAGAGAAAGCTTTG------ 940

Tr-kit_c18-4_2.7kb ------------------------------------------------------------

Tr-kit_c18-4_2.9kb ----------------------------------------------GACTCCTG------ 89

Tr-kit_c18-4_4.0kb -----------AAGGATTGATTCTGCCTC--------ATTGTGACAGATTAATA------ 413

Tr-kit_CRL2053_1.9kb ------------------------------------------------------------

Tr-kit_CRL2053_2.7kb ------------------------------------------------------------

Tr-kit_CRL2053_3.1kb ------------------------------------------------------------

Tr-kit_CRL2053_3.9kb -----------GAGTTCTATTTACGTGTTTGTTAGAGATCCTGCCAAACTTTTC------ 450

NM_001122733.1 ATGCTCCAGTG----TGTGGCAGAGGGATTCCCGGAGCCCACAATAGATTGGTATTTTTG 1402

NM_021099.3 ATGCTCCAGTG----TGTGGCAGAGGGATTCCCGGAGCCCACAATAGATTGGTATTTTTG 1398

X65997.1 ----TT-------------------AAAAT--------------TGGGT---------TG 239

Full_length ATGCTCCAGTG----TGTGGCAGAGGGATTCCCGGAGCCCACAATAGATTGGTATTTTTG 1396

Short_3_end_UTR ATGCTCCAGTG----TGTGGCAGAGGGATTCCCGGAGCCCACAATAGATTGGTATTTTTG 1396

SSCs_specific ----TTCCCTGAATGTGCCATGAGGGAAATGGTTTAGTTTGGGATAGGTGG-------TG 989

Tr-kit_c18-4_2.7kb ------------------------------------------------------------

Tr-kit_c18-4_2.9kb ----CC-----------------------------------------ATCA-------TG 97

Tr-kit_c18-4_4.0kb ----TCC-----------------AGAAATCCT---------GATAGAC---------TG 434

Tr-kit_CRL2053_1.9kb ------------------------------------------------------------

Tr-kit_CRL2053_2.7kb ------------------------------------------------------------

Tr-kit_CRL2053_3.1kb ------------------------------------------------------------

Tr-kit_CRL2053_3.9kb ----CTGGTT--------------GGCCTTCCCTT-------GTTTGGCAA-------AG 478

NM_001122733.1 TACAGGAGCAGAGCAAAGGTGT----ACCACTCCTGTCTCACCAGTGGACGTACAGGTCC 1458

NM_021099.3 TACAGGAGCAGAGCAAAGGTGT----ACCACTCCTGTCTCACCAGTGGACGTACAGGTCC 1454

X65997.1 GA-AA--GCAA--T---------------------------------------------T 249

Full_length TACAGGAGCAGAGCAAAGGTGT----ACCACTCCTGTCTCACCAGTGGACGTACAGGTCC 1452

Short_3_end_UTR TACAGGAGCAGAGCAAAGGTGT----ACCACTCCTGTCTCACCAGTGGACGTACAGGTCC 1452

SSCs_specific GT-GGCGGCGAGGCTGTTGTCCTGCGGCTGCTGGCTCACAATCATGGTTCCCTTCCTTGC 1048

Tr-kit_c18-4_2.7kb ------------------------------------------------------------

Tr-kit_c18-4_2.9kb GA-AG------------------------------------------------------- 101

Tr-kit_c18-4_4.0kb TA-AGAGGCAAGGT---------------------------------------------C 448

Tr-kit_CRL2053_1.9kb ------------------------------------------------------------

Tr-kit_CRL2053_2.7kb ------------------------------------------------------------

Tr-kit_CRL2053_3.1kb ------------------------------------------------------------

Tr-kit_CRL2053_3.9kb AA-GACAGCGACGCG--------------------------------------------- 492

NM_001122733.1 AGAATGTATCTGTGTCACCATTTGGAAAACTGGTGGTTCAGAGTTCCATAGACTCCAGCG 1518

NM_021099.3 AGAATGTATCTGTGTCACCATTTGGAAAACTGGTGGTTCAGAGTTCCATAGACTCCAGCG 1514

X65997.1 ATAGTCATT---------------------------------------AGAGC------- 263

Full_length AGAATGTATCTGTGTCACCATTTGGAAAACTGGTGGTTCAGAGTTCCATAGACTCCAGCG 1512

Short_3_end_UTR AGAATGTATCTGTGTCACCATTTGGAAAACTGGTGGTTCAGAGTTCCATAGACTCCAGCG 1512

SSCs_specific AGAGCAAATCCAGGCCCACACT-------CTGTTCACGCCGCTGCTCATTGGCTTTGTGG 1101

Tr-kit_c18-4_2.7kb ------------------------------------------------------------

Tr-kit_c18-4_2.9kb ATGACGAGC----------------------------------------TGGCT------ 115

Tr-kit_c18-4_4.0kb AGAGTAAGC---------------------------------------TTGGC------- 462

Tr-kit_CRL2053_1.9kb ------------------------------------------------------------

Tr-kit_CRL2053_2.7kb ------------------------------------------------------------

Tr-kit_CRL2053_3.1kb ------------------------------------------------------------

Tr-kit_CRL2053_3.9kb CTGGTCCGC----------------------------------------TGCCCT----- 507

NM_001122733.1 TCTT--CCGGCACAACGGCACGGTGGAGTGTAAGGCCTCCAACGATGTGGGCAAGAGTTC 1576

NM_021099.3 TCTT--CCGGCACAACGGCACGGTGGAGTGTAAGGCCTCCAACGATGTGGGCAAGAGTTC 1572

X65997.1 ------C---C-C------------GA--------------------------------- 268

Full_length TCTT--CCGGCACAACGGCACGGTGGAGTGTAAGGCCTCCAACGATGTGGGCAAGAGTTC 1570

Short_3_end_UTR TCTT--CCGGCACAACGGCACGGTGGAGTGTAAGGCCTCCAACGATGTGGGCAAGAGTTC 1570

SSCs_specific TCGCAGCTGGCGC------------GA-TGGGGATC--------------ATTGTGATGG 1134

Tr-kit_c18-4_2.7kb ------------------------------------------------------------

Tr-kit_c18-4_2.9kb ------CTG----------------GA--------------------------------- 120

Tr-kit_c18-4_4.0kb ------CTGAC-C------------GAGTGGAAGGA--------------ACTGGAATG- 488

Tr-kit_CRL2053_1.9kb ------------------------------------------------------------

Tr-kit_CRL2053_2.7kb ------------------------------------------------------------

Tr-kit_CRL2053_3.1kb ------------------------------------------------------------

Tr-kit_CRL2053_3.9kb ------CTGAC-A------------GACCCACAGGT--------------GTCCAATTA- 533

NM_001122733.1 CGCCTTCTTTAACTTTGCATTTAAAGGTAACAACAAAGAGCAAATCCAGGCCCACACTCT 1636

NM_021099.3 CGCCTTCTTTAACTT---TGCATTTAAAG---------AGCAAATCCAGGCCCACACTCT 1620

X65997.1 -----TCCTG--------------TGA-----------AACA---CAAA----------- 284

Full_length CGCCTTCTTTAACTT---TGCATTTAAAG---------AGCAAATCCAGGCCCACACTCT 1618

Short_3_end_UTR CGCCTTCTTTAACTT------TGCATTTA------AAGAGCAAATCCAGGCCCACACTCT 1618

SSCs_specific TGCTCACCTACAAAT---ATTTGCAGGTG---------AGCA---TTGAATTGTTCT--- 1176

Tr-kit_c18-4_2.7kb ------------------------------------------------------------

Tr-kit_c18-4_2.9kb ------CCTG-------------------------------------------------- 124

Tr-kit_c18-4_4.0kb ---TCTCCTG--------------ACATA---------AGCA---CTAACTGTGCGC--- 516

Tr-kit_CRL2053_1.9kb ------------------------------------------------------------

Tr-kit_CRL2053_2.7kb ------------------------------------------------------------

Tr-kit_CRL2053_3.1kb ------------------------------------------------------------

Tr-kit_CRL2053_3.9kb ---TTCCCT-------------------------------CA---TCGAGTGTG------ 550

NM_001122733.1 GTTCACGCCGCTGCTCATTGGCTTTGTGGTTGCAGCTGGCGCGATGGGGATCATTGTGAT 1696

NM_021099.3 GTTCACGCCGCTGCTCATTGGCTTTGTGGTTGCAGCTGGCGCGATGGGGATCATTGTGAT 1680

X65997.1 ----------------AC-------------GGG--------AATA-------------- 293

Full_length GTTCACGCCGCTGCTCATTGGCTTTGTGGTCGCAGCTGGCGCGATGGGGATCATTGTGAT 1678

Short_3_end_UTR GTTCACGCCGCTGCTCATTGGCTTTGTGGTTGCAGCTGGCGCGATGGGGATCATTGTGAT 1678

SSCs_specific CTTCCTGGGGACGCCAAG-------------GCGGCAGGGCAGGCACTGATTGTTCAGCG 1223

Tr-kit_c18-4_2.7kb ------------------------------------------------------------

Tr-kit_c18-4_2.9kb ------------------------------------------------------------

Tr-kit_c18-4_4.0kb GCTCGCGCGCGTGCACAC-------------GCGGCGCGCACAACA-------------- 549

Tr-kit_CRL2053_1.9kb ------------------------------------------------------------

Tr-kit_CRL2053_2.7kb ------------------------------------------------------------

Tr-kit_CRL2053_3.1kb ------------------------------------------------------------

Tr-kit_CRL2053_3.9kb ----ATGGGAAATCTCTC-------------CCCACGGACCTGACG-------------- 579

NM_001122733.1 GGTGCTCACCTACAAATATT---------TGCAGAAACCCATGTATGAAGTACAATGGAA 1747

NM_021099.3 GGTGCTCACCTACAAATATT---------TGCAGAAACCCATGTATGAAGTACAATGGAA 1731

X65997.1 -----TCACTT-------------------------GCAC---CATAATTTTTA------ 314

Full_length GGTGCTCACCTACAAATATT---------TGCAGAAACCCATGTATGAAGTACAATGGAA 1729

Short_3_end_UTR GGTGCTCACCTACAAATATT---------TGCAGAAACCCATGTATGAAGTACAATGGAA 1729

SSCs_specific GGTG-ACACATCTTTCTTTTCCTTTCTCCTCCAGAAACCCATGTATGAAGTACAATGGAA 1282

Tr-kit_c18-4_2.7kb ------------------------------------------------------------

Tr-kit_c18-4_2.9kb ----------------------------------------------GATGATT------- 131

Tr-kit_c18-4_4.0kb -----ACACAC-------------------------ACACACACACAGAGATCAGACACA 579

Tr-kit_CRL2053_1.9kb ------------------------------------------------------------

Tr-kit_CRL2053_2.7kb ------------------------------------------------------------

Tr-kit_CRL2053_3.1kb ------------------------------------------------------------

Tr-kit_CRL2053_3.9kb ------TTTGT--------------------------CCCAAACCCCAAGGCTGGCATCA 607

NM_001122733.1 GGTTGTCGAGGAGATAAATGGAAACAATTATGTTTACATAGA-CCCGACGCAACTTCCTT 1806

NM_021099.3 GGTTGTCGAGGAGATAAATGGAAACAATTATGTTTACATAGA-CCCGACGCAACTTCCTT 1790

X65997.1 --TTTTCGG---------------------TG--TGC-TAA--------ATACTTT---- 336

Full_length GGTTGTCGAGGAGATAAATGGAAACAATTATGTTTACATAGA-CCCGACGCAACTCCCTT 1788

Short_3_end_UTR GGTTGTCGAGGAGATAAATGGAAACAATTATGTTTACATAGA-CCCGACGCAACTTCCTT 1788

SSCs_specific GGTTGTCGAGGAGATAAATGGAAACAATTATGTTTACATAGA-CCCGACGCAACTTCCTT 1341

Tr-kit_c18-4_2.7kb ------------------------------------------------------------

Tr-kit_c18-4_2.9kb ----------------------------------TGC----------------------- 134

Tr-kit_c18-4_4.0kb GCTCATCAG---------------------TGCCTGC-TAGT-CTGGGTAAATCTT---- 612

Tr-kit_CRL2053_1.9kb ------------------------------------------------------------

Tr-kit_CRL2053_2.7kb ------------------------------------------------------------

Tr-kit_CRL2053_3.1kb ------------------------------------------------------------

Tr-kit_CRL2053_3.9kb --CCATCAAAAACGTGAA-------------GCGCGCCTACCACCGGCTCTGTGTCC--- 649

NM_001122733.1 ATGATCACAAATGGGAGTTTCCCAGAAACAGGCTGAGTTTTGGAAAGACATTGGGAGCTG 1866

NM_021099.3 ATGATCACAAATGGGAGTTTCCCAGAAACAGGCTGAGTTTTGGAAAGACATTGGGAGCTG 1850

X65997.1 --------------------------AA--AAC-G-----------AAAGTT-------- 348

Full_length ATGATCACAAATGGGAGTTTCCCAGAAACAGGCTGAGTTTTGGAAAGACATTGGGAGCTG 1848

Short_3_end_UTR ATGATCACAAATGGGAGTTTCCCAGAAACAGGCTGAGTTTTGGAAAGACATTGGGAGCTG 1848

SSCs_specific ATGATCACAAATGGGAGTTTCCCAGAAACAGGCTGAGTTTTGGAAAGACATTGGGAGCTG 1401

Tr-kit_c18-4_2.7kb ------------------------------------------------------------

Tr-kit_c18-4_2.9kb ----------------------------TGAGCT-------------------------- 140

Tr-kit_c18-4_4.0kb --------------------------AATGAGCTG-----------GACGTTGGTAG--- 632

Tr-kit_CRL2053_1.9kb ------------------------------------------------------------

Tr-kit_CRL2053_2.7kb ------------------------------------------------------------

Tr-kit_CRL2053_3.1kb ------------------------------------------------------------

Tr-kit_CRL2053_3.9kb --------------------------GCTGTGCTG---CTCAGCGTGACGGTACATGGCT 680

NM_001122733.1 GTGCCTTCGGGAAGGTCGTTGAGGCCACTGCATATGGCTTGATTAAGTCGGATGCTGCCA 1926

NM_021099.3 GTGCCTTCGGGAAGGTCGTTGAGGCCACTGCATATGGCTTGATTAAGTCGGATGCTGCCA 1910

X65997.1 ---TCTTT-------TTTTT-------TTTCAT----------------GT------AAA 369

Full_length GTGCCTTCGGGAAGGTCGTTGAGGCCACTGCATATGGCTTGATTAAGTCGGATGCTGCCA 1908

Short_3_end_UTR GTGCCTTCGGGAAGGTCGTTGAGGCCACTGCATATGGCTTGATTAAGTCGGATGCTGCCA 1908

SSCs_specific GTGCCTTCGGGAAGGTCGTTGAGGCCACTGCATATGGCTTGATTAAGTCGGATGCTGCCA 1461

Tr-kit_c18-4_2.7kb ------------------------------------------------------------

Tr-kit_c18-4_2.9kb ---TCTCC---------------------------------------------------- 145

Tr-kit_c18-4_4.0kb GCATTTTT-------TTATC-------CTGCAT------TGCCTCAGTTGTCCCATGAAA 672

Tr-kit_CRL2053_1.9kb ------------------------------------------------------------

Tr-kit_CRL2053_2.7kb ------------------------------------------------------------

Tr-kit_CRL2053_3.1kb ------------------------------------------------------------

Tr-kit_CRL2053_3.9kb GCATTCTGACAAA--TTCAC-------CCTCAA-------AGTGCGGGCAGCCATCAAGG 724

NM_001122733.1 TGACAGTTGCCGTGAAGATGCTCAAACCAAGTGCCCATTTAACAGAAAGAGAGGCCCTAA 1986

NM_021099.3 TGACAGTTGCCGTGAAGATGCTCAAACCAAGTGCCCATTTAACAGAAAGAGAGGCCCTAA 1970

X65997.1 CACCA--TTGTA-----GTATT-AAA----AT---CATCT-----------------TC- 396

Full_length TGACAGTTGCCGTGAAGATGCTCAAACCAAGTGCCCATTTAACAGAAAGAGAGGCCCTAA 1968

Short_3_end_UTR TGACAGTTGCCGTGAAGATGCTCAAACCAAGTGCCCATTTAACAGAAAGAGAGGCCCTAA 1968

SSCs_specific TGACAGTTGCCGTGAAGATGCTCAAACCAAGTGCCCATTTAACAGAAAGAGAGGCCCTAA 1521

Tr-kit_c18-4_2.7kb ------------------------------------------------------------

Tr-kit_c18-4_2.9kb TACCA------------------------------------------------------- 150

Tr-kit_c18-4_4.0kb TAACAACTCCTG-----GTATTTGAA----GT---TATTT-----------------TTG 703

Tr-kit_CRL2053_1.9kb ------------------------------------------------------------

Tr-kit_CRL2053_2.7kb ------------------------------------------------------------

Tr-kit_CRL2053_3.1kb ------------------------------------------------------------

Tr-kit_CRL2053_3.9kb CTATCCCTGTTGTGTCTGTGCCTGAAACAAGT---CACCT-------------------- 761

NM_001122733.1 TGTCGGAACTGAAGGT---CCTGAGCTACCTGGGCAATCACATGAATATTGTGAACCTGC 2043

NM_021099.3 TGTCGGAACTGAAGGT---CCTGAGCTACCTGGGCAATCACATGAATATTGTGAACCTGC 2027

X65997.1 TCTCG-GAGAGCTGAA---A-TGAA-----TGGCT----------GTT--GCTGTCTTTC 434

Full_length TGTCGGAACTGAAGGT---CCTGAGCTACCTGGGCAATCACATGAATATTGTGAACCTGC 2025

Short_3_end_UTR TGTCGGAACTGAAGGT---CCTGAGCTACCTGGGCAATCACATGAATATTGTGAACCTGC 2025

SSCs_specific TGTCGGAACTGAAGGT---CCTGAGCTACCTGGGCAATCACATGAATATTGTGAACCTGC 1578

Tr-kit_c18-4_2.7kb ------------------------------------------------------------

Tr-kit_c18-4_2.9kb ----------GGTGGC---CAAGGGC---ATGGC-----------GTTC------CTCGC 177

Tr-kit_c18-4_4.0kb TTTTGCAAAAGATGAT---TCTGGGC---CTGGCTTATC---CGTGTTTAGGTAACTTCT 754

Tr-kit_CRL2053_1.9kb ------------------------------------------------------------

Tr-kit_CRL2053_2.7kb ------------------------------------------------------------

Tr-kit_CRL2053_3.1kb ------------------------------------------------------------

Tr-kit_CRL2053_3.9kb CCTTAAGAAAGGGGACACATTTACGG----TGGTGTGC------ACCATAAAAGATGTGT 811

NM_001122733.1 TTGGCGCATGCACGGTGGGAGGGCCCACCCTGGTCATTACAGAATATTGTTGCTATGGTG 2103

NM_021099.3 TTGGCGCATGCACGGTGGGAGGGCCCACCCTGGTCATTACAGAATATTGTTGCTATGGTG 2087

X65997.1 CT---------------------TTTCTCCC------CCAACAGT--------------- 452

Full_length TTGGCGCATGCACGGTGGGAGGGCCCACCCTGGTCATTACAGAATATTGTTGCTATGGTG 2085

Short_3_end_UTR TTGGCGCATGCACGGTGGGAGGGCCCACCCTGGTCATTACAGAATATTGTTGCTATGGTG 2085

SSCs_specific TTGGCGCATGCACGGTGGGAGGGCCCACCCTGGTCATTACAGAATATTGTTGCTATGGTG 1638

Tr-kit_c18-4_2.7kb ------------------------------------------------------------

Tr-kit_c18-4_2.9kb CT-----------------------------------CCAAGAAT--------------- 187

Tr-kit_c18-4_4.0kb CTAGTAGGTGCGTGAGGGGTGCACTTGTGTCAGAGGTCCAAGAGT--------------- 799

Tr-kit_CRL2053_1.9kb ------------------------------------------------------------

Tr-kit_CRL2053_2.7kb ------------------------------------------------------------

Tr-kit_CRL2053_3.1kb ----------------GGGAGGGCCCACCCTGGTCATTACAGAATATTGTTGCTATGGTG 44

Tr-kit_CRL2053_3.9kb CTA---CATCCGTGA-----------ACTCCATGTGGCTAAAGAT--------------- 842

NM_001122733.1 ATCTTTTGAATTTTTTG----AGAAGGAAGCGTGACTCGTTTATTTTCTCAAAGCAAGAA 2159

NM_021099.3 ATCTTTTGAATTTTTTG----AGAAGGAAGCGTGACTCGTTTATTTTCTCAAAGCAAGAA 2143

X65997.1 -------GTAT-----T----CACAGAGA----------TTTG----------------G 470

Full_length ATCTTTTGAATTTTTTG----AGAAGGAAGCGTGACTCGTTTATTTTCTCAAAGCAAGAA 2141

Short_3_end_UTR ATCTTTTGAATTTTTTG----AGAAGGAAGCGTGACTCGTTTATTTTCTCAAAGCAAGAA 2141

SSCs_specific ATCTTTTGAATTTTTTG----AGAAGGAAGCGTGACTCGTTTATTTTCTCAAAGCAAGAA 1694

Tr-kit_c18-4_2.7kb ------------------------------------------------------------

Tr-kit_c18-4_2.9kb ------TGTAT-----T----CACAGAGA----------TTTG----------------- 205

Tr-kit_c18-4_4.0kb -------GAAT-----G----GGAAGGGA----------TTTG----------------G 817

Tr-kit_CRL2053_1.9kb ----------------------------------------------------------AA 100

Tr-kit_CRL2053_2.7kb ----------------------------------------------------------AA 872

Tr-kit_CRL2053_3.1kb ATCTTTTGAATTTTTTG----AGAAGGAAGCGTGACTCGTTTATTTTCTCAAAGCAAGAA 100

Tr-kit_CRL2053_3.9kb -------GAACCCTCAGCCTCAGCACATAG---------CCCA--------------GAA 872

NM_001122733.1 GAGCAGGCAGAAGCGGCACTTTATAAGAACCTTCTGCACTCAACGGAGCCTTCCTGTGAC 2219

NM_021099.3 GAGCAGGCAGAAGCGGCACTTTATAAGAACCTTCTGCACTCAACGGAGCCTTCCTGTGAC 2203

X65997.1 ---CAGCCAG-----GAAT----------------------------------------- 481

Full_length GAGCAGGCAGAAGCGGCACTTTATAAGAACCTTCTGCACTCAACGGAGCCTTCCTGTGAC 2201

Short_3_end_UTR GAGCAGGCAGAAGCGGCACTTTATAAGAACCTTCTGCACTCAACGGAGCCTTCCTGTGAC 2201

SSCs_specific GAGCAGGCAGAAGCGGCACTTTATAAGAACCTTCTGCACTCAACGGAGCCTTCCTGTGAC 1754

Tr-kit_c18-4_2.7kb ------------------------------------------------------------

Tr-kit_c18-4_2.9kb --GCAGCCAG-----GAAT----------------------------------------- 217

Tr-kit_c18-4_4.0kb GGCCAGGCAGA----GAATTCTGAAACAACACTTAGCACC-----GAGCCCTTCTGCCTC 868

Tr-kit_CRL2053_1.9kb GAGCAGGCAGAAGCGGCACTTTATAAGAACCTTCTGCACTCAACGGAGCCTTCCTGTGAC 160

Tr-kit_CRL2053_2.7kb GAGCAGGCAGAAGCGGCACTTTATAAGAACCTTCTGCACTCAACGGAGCCTTCCTGTGAC 932

Tr-kit_CRL2053_3.1kb GAGCAGGCAGAAGCGGCACTTTATAAGAACCTTCTGCACTCAACGGAGCCTTCCTGTGAC 160

Tr-kit_CRL2053_3.9kb GAGCAGGCAGAAGCGGCACTTTATAAGAACCTTCTGCACTCAACGGAGCCTTCCTGTGAC 932

NM_001122733.1 AGTTCAAATGAATATATGGACATGAAGCCTGGCGTTTCCT--------ACG-TGGTGCCA 2270

NM_021099.3 AGTTCAAATGAATATATGGACATGAAGCCTGGCGTTTCCT--------ACG-TGGTGCCA 2254

X65997.1 -------------AT-------------CCTCCTCACTC---------ACG--------- 497

Full_length AGTTCAAATGAATATATGGACATGAAGCCTGGCGTTTCCT--------ACG-TGGTGCCA 2252

Short_3_end_UTR AGTTCAAATGAATATATGGACATGAAGCCTGGCGTTTCCT--------ACG-TGGTGCCA 2252

SSCs_specific AGTTCAAATGAATATATGGACATGAAGCCTGGCGTTTCCT--------ACG-TGGTGCCA 1805

Tr-kit_c18-4_2.7kb ------------------------TA-TCCTCCTCACTC---------ACG--------- 17

Tr-kit_c18-4_2.9kb -------------AT-------------CCTCCTCACTC---------ACG--------- 233

Tr-kit_c18-4_4.0kb --TCTGTTTTGTCATTCCTACATGTAGCCCTCCTCATTCTGGTCAAAGATGACCGGACCA 926

Tr-kit_CRL2053_1.9kb AGTTCAAATGAATATATGGACATGAAGCCTGGCGTTTCCT--------ACG-TGGTGCCA 211

Tr-kit_CRL2053_2.7kb AGTTCAAATGAATATATGGACATGAAGCCTGGCGTTTCCT--------ACG-TGGTGCCA 983

Tr-kit_CRL2053_3.1kb AGTTCAAATGAATATATGGACATGAAGCCTGGCGTTTCCT--------ACG-TGGTGCCA 211

Tr-kit_CRL2053_3.9kb AGTTCAAATGAATATATGGACATGAAGCCTGGCGTTTCCT--------ACG-TGGTGCCA 983

* * * * *

NM_001122733.1 AC-----------------CAAGACAGACAA---GAGGAGATCCGCAAGAATAGACTCGT 2310

NM_021099.3 AC-----------------CAAGACAGACAA---GAGGAGATCCGCAAGAATAGACTCGT 2294

X65997.1 ----------------------GGCGGAT------------------------------- 504

Full_length AC-----------------CAAGACAGACAA---GAGGAGATCCGCAAGAATAGACTCGT 2292

Short_3_end_UTR AC-----------------CAAGACAGACAA---GAGGAGATCCGCAAGAATAGACTCGT 2292

SSCs_specific AC-----------------CAAGACAGACAA---GAGGAGATCCGCAAGAATAGACTCGT 1845

Tr-kit_c18-4_2.7kb ----------------------GGCGGAT------------------------------- 24

Tr-kit_c18-4_2.9kb ----------------------GGCGGAT------------------------------- 240

Tr-kit_c18-4_4.0kb ACAGCCAGCCGTCATGGTATAAGGCAGATGGTGTGAAGGGATGCCACTAGAAAGACTAAT 986

Tr-kit_CRL2053_1.9kb AC-----------------CAAGACAGACAA---GAGGAGATCCGCAAGAATAGACTCGT 251

Tr-kit_CRL2053_2.7kb AC-----------------CAAGACAGACAA---GAGGAGATCCGCAAGAATAGACTCGT 1023

Tr-kit_CRL2053_3.1kb AC-----------------CAAGACAGACAA---GAGGAGATCCGCAAGAATAGACTCGT 251

Tr-kit_CRL2053_3.9kb AC-----------------CAAGACAGACAA---GAGGAGATCCGCAAGAATAGACTCGT 1023

* * **

NM_001122733.1 ACATAGAAAGAGACGTGACTCCTGCCATCATGGAAGATGACGAGCTGGCTCTGGACC--- 2367

NM_021099.3 ACATAGAAAGAGACGTGACTCCTGCCATCATGGAAGATGACGAGCTGGCTCTGGACC--- 2351

X65997.1 ---------------------CA--CA--------------------------------- 508

Full_length ACATAGAAAGAGACGTGACTCCTGCCATCATGGAAGATGACGAGCTGGCTCTGGACC--- 2349

Short_3_end_UTR ACATAGAAAGAGACGTGACTCCTGCCATCATGGAAGATGACGAGCTGGCTCTGGACC--- 2349

SSCs_specific ACATAGAAAGAGACGTGACTCCTGCCATCATGGAAGATGACGAGCTGGCTCTGGACC--- 1902

Tr-kit_c18-4_2.7kb ---------------------CA--CA--------------------------------- 28

Tr-kit_c18-4_2.9kb ---------------------CA--CA--------------------------------- 244

Tr-kit_c18-4_4.0kb ATATAATATGGTGTATAAAACCA--CAGTATTTTAAGTGTTTTTTTTTCCTCATGCCACT 1044

Tr-kit_CRL2053_1.9kb ACATAGAAAGAGACGTGACTCCTGCCATCATGGAAGATGACGAGCTGGCTCTGGACC--- 308

Tr-kit_CRL2053_2.7kb ACATAGAAAGAGACGTGACTCCTGCCATCATGGAAGATGACGAGCTGGCTCTGGACC--- 1080

Tr-kit_CRL2053_3.1kb ACATAGAAAGAGACGTGACTCCTGCCATCATGGAAGATGACGAGCTGGCTCTGGACC--- 308

Tr-kit_CRL2053_3.9kb ACATAGAAAGAGACGTGACTCCTGCCATCATGGAAGATGACGAGCTGGCTCTGGACC--- 1080

* **

NM_001122733.1 -TGGATGATTTG-----CTGAGCTTCTCCTACCAGGTGGCCAAGGGCATGGCGTTCCTC- 2420

NM_021099.3 -TGGATGATTTG-----CTGAGCTTCTCCTACCAGGTGGCCAAGGGCATGGCGTTCCTC- 2404

X65997.1 ----AAGATTT------------------------GCGATTTCGG--------------- 525

Full_length -TGGATGATTTG-----CTGAGCTTCTCCTACCAGGTGGCCAAGGGCATGGCGTTCCTC- 2402

Short_3_end_UTR -TGGATGATTTG-----CTGAGCTTCTCCTACCAGGTGGCCAAGGGCATGGCGTTCCTC- 2402

SSCs_specific -TGGATGATTTG-----CTGAGCTTCTCCTACCAGGTGGCCAAGGGCATGGCGTTCCTC- 1955

Tr-kit_c18-4_2.7kb ----AAGATTT------------------------GCGATTTCGG--------------- 45

Tr-kit_c18-4_2.9kb ----AAGATTT------------------------GCGATTTCGG--------------- 261

Tr-kit_c18-4_4.0kb GCATAAAATTTAGAATCTTTAGCAGCTAAAACTAAGTGGTCTTGGCTATAGCCTTGCTGC 1104

Tr-kit_CRL2053_1.9kb -TGGATGATTTG-----CTGAGCTTCTCCTACCAGGTGGCCAAGGGCATGGCGTTCCTC- 361

Tr-kit_CRL2053_2.7kb -TGGATGATTTG-----CTGAGCTTCTCCTACCAGGTGGCCAAGGGCATGGCGTTCCTC- 1133

Tr-kit_CRL2053_3.1kb -TGGATGATTTG-----CTGAGCTTCTCCTACCAGGTGGCCAAGGGCATGGCGTTCCTC- 361

Tr-kit_CRL2053_3.9kb -TGGATGATTTG-----CTGAGCTTCTCCTACCAGGTGGCCAAGGGCATGGCGTTCCTC- 1133

* **** * * **

NM_001122733.1 ----GCCTCCAAGAATTGTATTCACAGAGATTTGGCAGCCAGGAATATCCTCCTCACTCA 2476

NM_021099.3 ----GCCTCCAAGAATTGTATTCACAGAGATTTGGCAGCCAGGAATATCCTCCTCACTCA 2460

X65997.1 ------------------------------------------------------------

Full_length ----GCCTCCAAGAATTGTATTCACAGAGATTTGGCAGCCAGGAATATCCTCCTCACTCA 2458

Short_3_end_UTR ----GCCTCCAAGAATTGTATTCACAGAGATTTGGCAGCCAGGAATATCCTCCTCACTCA 2458

SSCs_specific ----GCCTCCAAGAATTGTATTCACAGAGATTTGGCAGCCAGGAATATCCTCCTCACTCA 2011

Tr-kit_c18-4_2.7kb ------------------------------------------------------------

Tr-kit_c18-4_2.9kb ------------------------------------------------------------

Tr-kit_c18-4_4.0kb AAAGCAGTCTGGGAAATGTAGTTATAGTTGGGAGAGGCCATATATGTTGCTTGACAAAAA 1164

Tr-kit_CRL2053_1.9kb ----GCCTCCAAGAATTGTATTCACAGAGATTTGGCAGCCAGGAATATCCTCCTCACTCA 417

Tr-kit_CRL2053_2.7kb ----GCCTCCAAGAATTGTATTCACAGAGATTTGGCAGCCAGGAATATCCTCCTCACTCA 1189

Tr-kit_CRL2053_3.1kb ----GCCTCCAAGAATTGTATTCACAGAGATTTGGCAGCCAGGAATATCCTCCTCACTCA 417

Tr-kit_CRL2053_3.9kb ----GCCTCCAAGAATTGTATTCACAGAGATTTGGCAGCCAGGAATATCCTCCTCACTCA 1189

NM_001122733.1 CGGGCGGATCACAAAGATTTGCGATTTCGGGCTAGCCAGAGACATCA------GGAA--- 2527

NM_021099.3 CGGGCGGATCACAAAGATTTGCGATTTCGGGCTAGCCAGAGACATCA------GGAA--- 2511

X65997.1 ------------------------------GCTAGCCAGAGACATCA------GGAA--- 546

Full_length CGGGCGGATCACAAAGATTTGCGATTTCGGGCTAGCCAGAGACATCA------GGAA--- 2509

Short_3_end_UTR CGGGCGGATCACAAAGATTTGCGATTTCGGGCTAGCCAGAGACATCA------GGAA--- 2509

SSCs_specific CGGGCGGATCACAAAGATTTGCGATTTCGGGCTAGCCAGAGACATCA------GGAA--- 2062

Tr-kit_c18-4_2.7kb ------------------------------GCTAGCCAGAGACATCA------GGAA--- 66

Tr-kit_c18-4_2.9kb ------------------------------GCTAGCCAGAGACATCA------GGAA--- 282

Tr-kit_c18-4_4.0kb AAAAAAAAAAAGAAGAAGAAGAAGAAAGTTGCTAGTTGAGGCCATTACTAGAAGGAAGGT 1224

Tr-kit_CRL2053_1.9kb CGGGCGGATCACAAAGATTTGCGATTTCGGGCTAGCCAGAGACATCA------GGAA--- 468

Tr-kit_CRL2053_2.7kb CGGGCGGATCACAAAGATTTGCGATTTCGGGCTAGCCAGAGACATCA------GGAA--- 1240

Tr-kit_CRL2053_3.1kb CGGGCGGATCACAAAGATTTGCGATTTCGGGCTAGCCAGAGACATCA------GGAA--- 468

Tr-kit_CRL2053_3.9kb CGGGCGGATCACAAAGATTTGCGATTTCGGGCTAGCCAGAGACATCA------GGAA--- 1240

***** * *** * ****

NM_001122733.1 -------------------------------------------TGATTC----------- 2533

NM_021099.3 -------------------------------------------TGATTC----------- 2517

X65997.1 -------------------------------------------TGATTC----------- 552

Full_length -------------------------------------------TGATTC----------- 2515

Short_3_end_UTR -------------------------------------------TGATTC----------- 2515

SSCs_specific -------------------------------------------TGATTC----------- 2068

Tr-kit_c18-4_2.7kb -------------------------------------------TGATTC----------- 72

Tr-kit_c18-4_2.9kb -------------------------------------------TGATTC----------- 288

Tr-kit_c18-4_4.0kb TAGAACCCCTGGACTTCTCTGCTCTTAGTTTACTGTCCTATACTGACTCAACACCCCTAT 1284

Tr-kit_CRL2053_1.9kb -------------------------------------------TGATTC----------- 474

Tr-kit_CRL2053_2.7kb -------------------------------------------TGATTC----------- 1246

Tr-kit_CRL2053_3.1kb -------------------------------------------TGATTC----------- 474

Tr-kit_CRL2053_3.9kb -------------------------------------------TGATTC----------- 1246

*** **

NM_001122733.1 ------------------------GAATTAC-----------GTGGTCAAAGG------- 2551

NM_021099.3 ------------------------GAATTAC-----------GTGGTCAAAGG------- 2535

X65997.1 ------------------------GAATTAC-----------GTGGTCAAAGG------- 570

Full_length ------------------------GAATTAC-----------GTGGTCAAAGG------- 2533

Short_3_end_UTR ------------------------GAATTAC-----------GTGGTCAAAGG------- 2533

SSCs_specific ------------------------GAATTAC-----------GTGGTCAAAGG------- 2086

Tr-kit_c18-4_2.7kb ------------------------GAATTAC-----------GTGGTCAAAGG------- 90

Tr-kit_c18-4_2.9kb ------------------------GAATTAC-----------GTGGTCAAAGG------- 306

Tr-kit_c18-4_4.0kb TTTAAAGGGAGATATTAGAATTTTGAATTATAAGTAGGGGAGGTGGCTGGAGGTCACAAG 1344

Tr-kit_CRL2053_1.9kb ------------------------GAATTAC-----------GTGGTCAAAGG------- 492

Tr-kit_CRL2053_2.7kb ------------------------GAATTAC-----------GTGGTCAAAGG------- 1264

Tr-kit_CRL2053_3.1kb ------------------------GAATTAC-----------GTGGTCAAAGG------- 492

Tr-kit_CRL2053_3.9kb ------------------------GAATTAC-----------GTGGTCAAAGG------- 1264

****** **** ***

NM_001122733.1 ----------------------------------------AAAT---------------- 2555

NM_021099.3 ----------------------------------------AAAT---------------- 2539

X65997.1 ----------------------------------------AAAT---------------- 574

Full_length ----------------------------------------AAAT---------------- 2537

Short_3_end_UTR ----------------------------------------AAAT---------------- 2537

SSCs_specific ----------------------------------------AAAT---------------- 2090

Tr-kit_c18-4_2.7kb ----------------------------------------AAAT---------------- 94

Tr-kit_c18-4_2.9kb ----------------------------------------AAAT---------------- 310

Tr-kit_c18-4_4.0kb GTTTAAGGTCCTCGTCTATCGCTGTCTTCATTAGCTGCTTGAATTTGCTGTGTTCCGTTC 1404

Tr-kit_CRL2053_1.9kb ----------------------------------------AAAT---------------- 496

Tr-kit_CRL2053_2.7kb ----------------------------------------AAAT---------------- 1268

Tr-kit_CRL2053_3.1kb ----------------------------------------AAAT---------------- 496

Tr-kit_CRL2053_3.9kb ----------------------------------------AAAT---------------- 1268

***

NM_001122733.1 ---GCACGACTGCCCGTGAAGTGGATGGCACCAGAGAGCATTTTCAGCTGCGTGTACACA 2612

NM_021099.3 ---GCACGACTGCCCGTGAAGTGGATGGCACCAGAGAGCATTTTCAGCTGCGTGTACACA 2596

X65997.1 ---GCACGACTGCCCGTGAAGTGGATGGCACCAGAGAGCATTTTCAGCTGCGTGTACACA 631

Full_length ---GCACGACTGCCCGTGAAGTGGATGGCACCAGAGAGCATTTTCAGCTGCGTGTACACA 2594

Short_3_end_UTR ---GCACGACTGCCCGTGAAGTGGATGGCACCAGAGAGCATTTTCAGCTGCGTGTACACA 2594

SSCs_specific ---GCACGACTGCCCGTGAAGTGGATGGCACCAGAGAGCATTTTCAGCTGCGTGTACACA 2147

Tr-kit_c18-4_2.7kb ---GCACGACTGCCCGTGAAGTGGATGGCACCAGAGAGCATTTTCAGCTGCGTGTACACA 151

Tr-kit_c18-4_2.9kb ---GCACGACTGCCCGTGAAGTGGATGGCACCAGAGAGCATTTTCAGCTGCGTGTACACA 367

Tr-kit_c18-4_4.0kb TAGGCACGACTGCCCGTGAAGTGGATGGCACCAGAGAGCATTTTCAGCTGCGTGTACACA 1464

Tr-kit_CRL2053_1.9kb ---GCACGACTGCCCGTGAAGTGGATGGCACCAGAGAGCATTTTCAGCTGCGTGTACACA 553

Tr-kit_CRL2053_2.7kb ---GCACGACTGCCCGTGAAGTGGATGGCACCAGAGAGCATTTTCAGCTGCGTGTACACA 1325

Tr-kit_CRL2053_3.1kb ---GCACGACTGCCCGTGAAGTGGATGGCACCAGAGAGCATTTTCAGCTGCGTGTACACA 553

Tr-kit_CRL2053_3.9kb ---GCACGACTGCCCGTGAAGTGGATGGCACCAGAGAGCATTTTCAGCTGCGTGTACACA 1325

*********************************************************

NM_001122733.1 TTTGAAAGTGATGTCTGGTCCTATGGGATTTTCCTCTGGGAGCTCTTCTCCTTAGGAAGC 2672

NM_021099.3 TTTGAAAGTGATGTCTGGTCCTATGGGATTTTCCTCTGGGAGCTCTTCTCCTTAGGAAGC 2656

X65997.1 TTTGAAAGTGATGTCTGGTCCTATGGGATTTTCCTCTGGGAGCTCTTCTCCTTAGGAAGC 691

Full_length TTTGAAAGTGATGTCTGGTCCTATGGGATTTTCCTCTGGGAGCTCTTCTCCTTAGGAAGC 2654

Short_3_end_UTR TTTGAAAGTGATGTCTGGTCCTATGGGATTTTCCTCTGGGAGCTCTTCTCCTTAGGAAGC 2654

SSCs_specific TTTGAAAGTGATGTCTGGTCCTATGGGATTTTCCTCTGGGAGCTCTTCTCCTTAGGAAGC 2207

Tr-kit_c18-4_2.7kb TTTGAAAGTGATGTCTGGTCCTATGGGATTTTCCTCTGGGAGCTCTTCTCCTTAGGAAGC 211

Tr-kit_c18-4_2.9kb TTTGAAAGTGATGTCTGGTCCTATGGGATTTTCCTCTGGGAGCTCTTCTCCTTAGGAAGC 427

Tr-kit_c18-4_4.0kb TTTGAAAGTGATGTCTGGTCCTATGGGATTTTCCTCTGGGAGCTCTTCTCCTTAGGAAGC 1524

Tr-kit_CRL2053_1.9kb TTTGAAAGTGATGTCTGGTCCTATGGGATTTTCCTCTGGGAGCTCTTCTCCTTAGGAAGC 613

Tr-kit_CRL2053_2.7kb TTTGAAAGTGATGTCTGGTCCTATGGGATTTTCCTCTGGGAGCTCTTCTCCTTAGGAAGC 1385

Tr-kit_CRL2053_3.1kb TTTGAAAGTGATGTCTGGTCCTATGGGATTTTCCTCTGGGAGCTCTTCTCCTTAGGAAGC 613

Tr-kit_CRL2053_3.9kb TTTGAAAGTGATGTCTGGTCCTATGGGATTTTCCTCTGGGAGCTCTTCTCCTTAGGAAGC 1385

************************************************************

NM_001122733.1 AGCCCCTACCCAGGGATGCCGGTCGACTCCAAGTTCTACAAGATGATCAAGGAAGGCTTC 2732

NM_021099.3 AGCCCCTACCCAGGGATGCCGGTCGACTCCAAGTTCTACAAGATGATCAAGGAAGGCTTC 2716

X65997.1 AGCCCCTACCCAGGGATGCCGGTCGACTCCAAGTTCTACAAGATGATCAAGGAAGGCTTC 751

Full_length AGCCCCTACCCAGGGATGCCGGTCGACTCCAAGTTCTACAAGATGATCAAGGAAGGCTTC 2714

Short_3_end_UTR AGCCCCTACCCAGGGATGCCGGTCGACTCCAAGTTCTACAAGATGATCAAGGAAGGCTTC 2714

SSCs_specific AGCCCCTACCCAGGGATGCCGGTCGACTCCAAGTTCTACAAGATGATCAAGGAAGGCTTC 2267

Tr-kit_c18-4_2.7kb AGCCCCTACCCAGGGATGCCGGTCGACTCCAAGTTCTACAAGATGATCAAGGAAGGCTTC 271

Tr-kit_c18-4_2.9kb AGCCCCTACCCAGGGATGCCGGTCGACTCCAAGTTCTACAAGATGATCAAGGAAGGCTTC 487

Tr-kit_c18-4_4.0kb AGCCCCTACCCAGGGATGCCGGTCGACTCCAAGTTCTACAAGATGATCAAGGAAGGCTTC 1584

Tr-kit_CRL2053_1.9kb AGCCCCTACCCAGGGATGCCGGTCGACTCCAAGTTCTACAAGATGATCAAGGAAGGCTTC 673

Tr-kit_CRL2053_2.7kb AGCCCCTACCCAGGGATGCCGGTCGACTCCAAGTTCTACAAGATGATCAAGGAAGGCTTC 1445

Tr-kit_CRL2053_3.1kb AGCCCCTACCCAGGGATGCCGGTCGACTCCAAGTTCTACAAGATGATCAAGGAAGGCTTC 673

Tr-kit_CRL2053_3.9kb AGCCCCTACCCAGGGATGCCGGTCGACTCCAAGTTCTACAAGATGATCAAGGAAGGCTTC 1445

************************************************************

NM_001122733.1 CGGATGGTCAGCCCGGAGCACGCGCCTGCCGAAATGTATGACGTCATGAAGACTTGCTGG 2792

NM_021099.3 CGGATGGTCAGCCCGGAGCACGCGCCTGCCGAAATGTATGACGTCATGAAGACTTGCTGG 2776

X65997.1 CGGATGGTCAGCCCGGAGCACGCGCCTGCCGAAATGTATGACGTCATGAAGACTTGCTGG 811

Full_length CGGATGGTCAGCCCGGAGCACGCGCCTGCCGAAATGTATGACGTCATGAAGACTTGCTGG 2774

Short_3_end_UTR CGGATGGTCAGCCCGGAGCACGCGCCTGCCGAAATGTATGACGTCATGAAGACTTGCTGG 2774

SSCs_specific CGGATGGTCAGCCCGGAGCACGCGCCTGCCGAAATGTATGACGTCATGAAGACTTGCTGG 2327

Tr-kit_c18-4_2.7kb CGGATGGTCAGCCCGGAGCACGCGCCTGCCGAAATGTATGACGTCATGAAGACTTGCTGG 331

Tr-kit_c18-4_2.9kb CGGATGGTCAGCCCGGAGCACGCGCCTGCCGAAATGTATGACGTCATGAAGACTTGCTGG 547

Tr-kit_c18-4_4.0kb CGGATGGTCAGCCCGGAGCACGCGCCTGCCGAAATGTATGACGTCATGAAGACTTGCTGG 1644

Tr-kit_CRL2053_1.9kb CGGATGGTCAGCCCGGAGCACGCGCCTGCCGAAATGTATGACGTCATGAAGACTTGCTGG 733

Tr-kit_CRL2053_2.7kb CGGATGGTCAGCCCGGAGCACGCGCCTGCCGAAATGTATGACGTCATGAAGACTTGCTGG 1505

Tr-kit_CRL2053_3.1kb CGGATGGTCAGCCCGGAGCACGCGCCTGCCGAAATGTATGACGTCATGAAGACTTGCTGG 733

Tr-kit_CRL2053_3.9kb CGGATGGTCAGCCCGGAGCACGCGCCTGCCGAAATGTATGACGTCATGAAGACTTGCTGG 1505

************************************************************

NM_001122733.1 GACGCTGACCCCTTGAAAAGGCCAACATTCAAGCAGGTTGTCCAACTTATTGAGAAGCAG 2852

NM_021099.3 GACGCTGACCCCTTGAAAAGGCCAACATTCAAGCAGGTTGTCCAACTTATTGAGAAGCAG 2836

X65997.1 GACGCTGACCCCTTGAAAAGGCCAACATTCAAGCAGGTTGTCCAACTTATTGAGAAGCAG 871

Full_length GACGCTGACCCCTTGAAAAGGCCAACATTCAAGCAGGTTGTCCAACTTATTGAGAAGCAG 2834

Short_3_end_UTR GACGCTGACCCCTTGAAAAGGCCAACATTCAAGCAGGTTGTCCAACTTATTGAGAAGCAG 2834

SSCs_specific GACGCTGACCCCTTGAAAAGGCCAACATTCAAGCAGGTTGTCCAACTTATTGAGAAGCAG 2387

Tr-kit_c18-4_2.7kb GACGCTGACCCCTTGAAAAGGCCAACATTCAAGCAGGTTGTCCAACTTATTGAGAAGCAG 391

Tr-kit_c18-4_2.9kb GACGCTGACCCCTTGAAAAGGCCAACATTCAAGCAGGTTGTCCAACTTATTGAGAAGCAG 607

Tr-kit_c18-4_4.0kb GACGCTGACCCCTTGAAAAGGCCAACATTCAAGCAGGTTGTCCAACTTATTGAGAAGCAG 1704

Tr-kit_CRL2053_1.9kb GACGCTGACCCCTTGAAAAGGCCAACATTCAAGCAGGTTGTCCAACTTATTGAGAAGCAG 793

Tr-kit_CRL2053_2.7kb GACGCTGACCCCTTGAAAAGGCCAACATTCAAGCAGGTTGTCCAACTTATTGAGAAGCAG 1565

Tr-kit_CRL2053_3.1kb GACGCTGACCCCTTGAAAAGGCCAACATTCAAGCAGGTTGTCCAACTTATTGAGAAGCAG 793

Tr-kit_CRL2053_3.9kb GACGCTGACCCCTTGAAAAGGCCAACATTCAAGCAGGTTGTCCAACTTATTGAGAAGCAG 1565

************************************************************

NM_001122733.1 ATCTCGGACAGCACCAAGCACATTTACTCCAACTTGGCAAACTGCAACCCCAACCCAGAG 2912

NM_021099.3 ATCTCGGACAGCACCAAGCACATTTACTCCAACTTGGCAAACTGCAACCCCAACCCAGAG 2896

X65997.1 ATCTCGGACAGCACCAAGCACATTTACTCCAACTTGGCAAACTGCAACCCCAACCCAGAG 931

Full_length ATCTCGGACAGCACCAAGCACATTTACTCCAACTTGGCAAACTGCAACCCCAACCCAGAG 2894

Short_3_end_UTR ATCTCGGACAGCACCAAGCACATTTACTCCAACTTGGCAAACTGCAACCCCAACCCAGAG 2894

SSCs_specific ATCTCGGACAGCACCAAGCACATTTACTCCAACTTGGCAAACTGCAACCCCAACCCAGAG 2447

Tr-kit_c18-4_2.7kb ATCTCGGACAGCACCAAGCACATTTACTCCAACTTGGCAAACTGCAACCCCAACCCAGAG 451

Tr-kit_c18-4_2.9kb ATCTCGGACAGCACCAAGCACATTTACTCCAACTTGGCAAACTGCAACCCCAACCCAGAG 667

Tr-kit_c18-4_4.0kb ATCTCGGACAGCACCAAGCACATTTACTCCAACTTGGCAAACTGCAACCCCAACCCAGAG 1764

Tr-kit_CRL2053_1.9kb ATCTCGGACAGCACCAAGCACATTTACTCCAACTTGGCAAACTGCAACCCCAACCCAGAG 853

Tr-kit_CRL2053_2.7kb ATCTCGGACAGCACCAAGCACATTTACTCCAACTTGGCAAACTGCAACCCCAACCCAGAG 1625

Tr-kit_CRL2053_3.1kb ATCTCGGACAGCACCAAGCACATTTACTCCAACTTGGCAAACTGCAACCCCAACCCAGAG 853

Tr-kit_CRL2053_3.9kb ATCTCGGACAGCACCAAGCACATTTACTCCAACTTGGCAAACTGCAACCCCAACCCAGAG 1625

************************************************************

NM_001122733.1 AACCCCGTGGTGGTGGACCATTCCGTGAGGGTCAACTCGGTGGGCAGCAGCGCCTCTTCT 2972

NM_021099.3 AACCCCGTGGTGGTGGACCATTCCGTGAGGGTCAACTCGGTGGGCAGCAGCGCCTCTTCT 2956

X65997.1 AACCCCGTGGTGGTGGACCATTCCGTGAGGGTCAACTCGGTGGGCAGCAGCGCCTCTTCT 991

Full_length AACCCCGTGGTGGTGGACCATTCCGTGAGGGTCAACTCGGTGGGCAGCAGCGCCTCTTCT 2954

Short_3_end_UTR AACCCCGTGGTGGTGGACCATTCCGTGAGGGTCAACTCGGTGGGCAGCAGCGCCTCTTCT 2954

SSCs_specific AACCCCGTGGTGGTGGACCATTCCGTGAGGGTCAACTCGGTGGGCAGCAGCGCCTCTTCT 2507

Tr-kit_c18-4_2.7kb AACCCCGTGGTGGTGGACCATTCCGTGAGGGTCAACTCGGTGGGCAGCAGCGCCTCTTCT 511

Tr-kit_c18-4_2.9kb AACCCCGTGGTGGTGGACCATTCCGTGAGGGTCAACTCGGTGGGCAGCAGCGCCTCTTCT 727

Tr-kit_c18-4_4.0kb AACCCCGTGGTGGTGGACCATTCCGTGAGGGTCAACTCGGTGGGCAGCAGCGCCTCTTCT 1824

Tr-kit_CRL2053_1.9kb AACCCCGTGGTGGTGGACCATTCCGTGAGGGTCAACTCGGTGGGCAGCAGCGCCTCTTCT 913

Tr-kit_CRL2053_2.7kb AACCCCGTGGTGGTGGACCATTCCGTGAGGGTCAACTCGGTGGGCAGCAGCGCCTCTTCT 1685

Tr-kit_CRL2053_3.1kb AACCCCGTGGTGGTGGACCATTCCGTGAGGGTCAACTCGGTGGGCAGCAGCGCCTCTTCT 913

Tr-kit_CRL2053_3.9kb AACCCCGTGGTGGTGGACCATTCCGTGAGGGTCAACTCGGTGGGCAGCAGCGCCTCTTCT 1685

************************************************************

NM_001122733.1 ACGCAGCCCCTGCTCGTGCACGAAGATGCCTGAGCAGAAACCCAAGTCCAACAGGCTTTG 3032

NM_021099.3 ACGCAGCCCCTGCTCGTGCACGAAGATGCCTGAGCAGAAACCCAAGTCCAACAGGCTTTG 3016

X65997.1 ACGCAGCCCCTGCTCGTGCACGAAGATGCCTGAGCAGAAACCCAAGTCCAACAGGCTTTG 1051

Full_length ACGCAGCCCCTGCTCGTGCACGAAGATGCCTGAGCAGAAACCCAAGTCCAACAGGCTTTG 3014

Short_3_end_UTR ACGCAGCCCCTGCTCGTGCACGAAGATGCCTGAGCAGAAACCCAAGTCCAACAGGCTTTG 3014

SSCs_specific ACGCAGCCCCTGCTCGTGCACGAAGATGCCTGAGCAGAAACCCAAGTCCAACAGGCTTTG 2567

Tr-kit_c18-4_2.7kb ACGCAGCCCCTGCTCGTGCACGAAGATGCCTGAGCAGAAACCCAAGTCCAACAGGCTTTG 571

Tr-kit_c18-4_2.9kb ACGCAGCCCCTGCTCGTGCACGAAGATGCCTGAGCAGAAACCCAAGTCCAACAGGCTTTG 787

Tr-kit_c18-4_4.0kb ACGCAGCCCCTGCTCGTGCACGAAGATGCCTGAGCAGAAACCCAAGTCCAACAGGCTTTG 1884

Tr-kit_CRL2053_1.9kb ACGCAGCCCCTGCTCGTGCACGAAGATGCCTGAGCAGAAACCCAAGTCCAACAGGCTTTG 973

Tr-kit_CRL2053_2.7kb ACGCAGCCCCTGCTCGTGCACGAAGATGCCTGAGCAGAAACCCAAGTCCAACAGGCTTTG 1745

Tr-kit_CRL2053_3.1kb ACGCAGCCCCTGCTCGTGCACGAAGATGCCTGAGCAGAAACCCAAGTCCAACAGGCTTTG 973

Tr-kit_CRL2053_3.9kb ACGCAGCCCCTGCTCGTGCACGAAGATGCCTGAGCAGAAACCCAAGTCCAACAGGCTTTG 1745

************************************************************

NM_001122733.1 CTGCTGTCTCCGACCCCGTCCTTCTGGCTTCTGTGATGGTTACTTGGTTTCCCTTTGACT 3092

NM_021099.3 CTGCTGTCTCCGACCCCGTCCTTCTGGCTTCTGTGATGGTTACTTGGTTTCCCTTTGACT 3076

X65997.1 CTGCTGTCTCCGACCCCGTCCTTCTGGCTTCTGTGATGGTTACTTGGTTTCCCTTTGACT 1111

Full_length CTGCTGTCTCCGACCCCGTCCTTCTGGCTTCTGTGATGGTTACTTGGTTTCCCTTTGACT 3074

Short_3_end_UTR CTGCTGTCTCCGACCCCGTCCTTCTGGCTTCTGTGATGGTTACTTGGTTTCCCTTTGACT 3074

SSCs_specific CTGCTGTCTCCGACCCCGTCCTTCTGGCTTCTGTGATGGTTACTTGGTTTCCCTTTGACT 2627

Tr-kit_c18-4_2.7kb CTGCTGTCTCCGACCCCGTCCTTCTGGCTTCTGTGATGGTTACTTGGTTTCCCTTTGACT 631

Tr-kit_c18-4_2.9kb CTGCTGTCTCCGACCCCGTCCTTCTGGCTTCTGTGATGGTTACTTGGTTTCCCTTTGACT 847

Tr-kit_c18-4_4.0kb CTGCTGTCTCCGACCCCGTCCTTCTGGCTTCTGTGATGGTTACTTGGTTTCCCTTTGACT 1944

Tr-kit_CRL2053_1.9kb CTGCTGTCTCCGACCCCGTCCTTCTGGCTTCTGTGATGGTTACTTGGTTTCCCTTTGACT 1033

Tr-kit_CRL2053_2.7kb CTGCTGTCTCCGACCCCGTCCTTCTGGCTTCTGTGATGGTTACTTGGTTTCCCTTTGACT 1805

Tr-kit_CRL2053_3.1kb CTGCTGTCTCCGACCCCGTCCTTCTGGCTTCTGTGATGGTTACTTGGTTTCCCTTTGACT 1033

Tr-kit_CRL2053_3.9kb CTGCTGTCTCCGACCCCGTCCTTCTGGCTTCTGTGATGGTTACTTGGTTTCCCTTTGACT 1805

************************************************************

NM_001122733.1 TGCATCCTATTCCAGGGTAGCGAGTTCCCCACCCCACCTCCAACCCCACTGTGATTCCGC 3152

NM_021099.3 TGCATCCTATTCCAGGGTAGCGAGTTCCCCACCCCACCTCCAACCCCACTGTGATTCCGC 3136

X65997.1 TGCATCCTATTCCAGGGTAGCGAGTTCCCCACCCCACCTCCAACCCCACTGTGATTCCGC 1171

Full_length TGCATCCTATTCCAGGGTAGCGAGTTCCCCACCCCACCTCCAACCCCACTGTGATTCCGC 3134

Short_3_end_UTR TGCATCCTATTCCAGGGTAGCGAGTTCCCCACCCCACCTCCAACCCCACTGTGATTCCGC 3134

SSCs_specific TGCATCCTATTCCAGGGTAGCGAGTTCCCCACCCCACCTCCAACCCCACTGTGATTCCGC 2687

Tr-kit_c18-4_2.7kb TGCATCCTATTCCAGGGTAGCGAGTTCCCCACCCCACCTCCAACCCCACTGTGATTCCGC 691

Tr-kit_c18-4_2.9kb TGCATCCTATTCCAGGGTAGCGAGTTCCCCACCCCACCTCCAACCCCACTGTGATTCCGC 907

Tr-kit_c18-4_4.0kb TGCATCCTATTCCAGGGTAGCGAGTTCCCCACCCCACCTCCAACCCCACTGTGATTCCGC 2004

Tr-kit_CRL2053_1.9kb TGCATCCTATTCCAGGGTAGCGAGTTCCCCACCCCACCTCCAACCCCACTGTGATTCCGC 1093

Tr-kit_CRL2053_2.7kb TGCATCCTATTCCAGGGTAGCGAGTTCCCCACCCCACCTCCAACCCCACTGTGATTCCGC 1865

Tr-kit_CRL2053_3.1kb TGCATCCTATTCCAGGGTAGCGAGTTCCCCACCCCACCTCCAACCCCACTGTGATTCCGC 1093

Tr-kit_CRL2053_3.9kb TGCATCCTATTCCAGGGTAGCGAGTTCCCCACCCCACCTCCAACCCCACTGTGATTCCGC 1865

************************************************************

NM_001122733.1 CTTTACGAGCACACACTTTAGTGGCCGATGGCCTTTTCTTTTCTGCCATCAGCCACCGTC 3212

NM_021099.3 CTTTACGAGCACACACTTTAGTGGCCGATGGCCTTTTCTTTTCTGCCATCAGCCACCGTC 3196

X65997.1 CTTTACGAGCACACACTTTAGTGGCCGATGGC-TTTTCTTTTCTGCCATCAGCCACCGTC 1230

Full_length CTTTACGAGCACACACTTTAGTGGCCGATGGCCTTTTCTTTTCTGCCATCAGCCACCGTC 3194

Short_3_end_UTR CTTTACGAGCACACACTTTAGTGGCCGATGGCCTTTTCTTTTCTGCCATCAGCCACCGTC 3194

SSCs_specific CTTTACGAGCACACACTTTAGTGGCCGATGGCCTTTTCTTTTCTGCCATCAGCCACCGTC 2747

Tr-kit_c18-4_2.7kb CTTTACGAGCACACACTTTAGTGGCCGATGGCCTTTTCTTTTCTGCCATCAGCCACCGTC 751

Tr-kit_c18-4_2.9kb CTTTACGAGCACACACTTTAGTGGCCGATGGCCTTTTCTTTTCTGCCATCAGCCACCGTC 967

Tr-kit_c18-4_4.0kb CTTTACGAGCACACACTTTAGTGGCCGATGGCCTTTTCTTTTCTGCCATCAGCCACCGTC 2064

Tr-kit_CRL2053_1.9kb CTTTACGAGCACACACTTTAGTGGCCGATGGCCTTTTCTTTTCTGCCATCAGCCACCGTC 1153

Tr-kit_CRL2053_2.7kb CTTTACGAGCACACACTTTAGTGGCCGATGGCCTTTTCTTTTCTGCCATCAGCCACCGTC 1925

Tr-kit_CRL2053_3.1kb CTTTACGAGCACACACTTTAGTGGCCGATGGCCTTTTCTTTTCTGCCATCAGCCACCGTC 1153

Tr-kit_CRL2053_3.9kb CTTTACGAGCACACACTTTAGTGGCCGATGGCCTTTTCTTTTCTGCCATCAGCCACCGTC 1925

******************************** ***************************

NM_001122733.1 CCGCTGCGAAGGTCCGAACTGTATGTATATATTTTCCCAATAGCAAAGTAGCTCCTACTG 3272

NM_021099.3 CCGCTGCGAAGGTCCGAACTGTATGTATATATTTTCCCAATAGCAAAGTAGCTCCTACTG 3256

X65997.1 CCGCTGCGAAGGTCCGAACTGTATGTATATATTTTCCCAATAGCAAAGTAGCTCCTACTG 1290

Full_length CCGCTGCGAAGGTCCGAACTGTATGTATATATTTTCCCAATAGCAAAGTAGCTCCTACTG 3254

Short_3_end_UTR CCGCTGCGAAGGTCCGAACTGTATGTATATATTTTCCCAATAGCAAAGTAGCTCCTACTG 3254

SSCs_specific CCGCTGCGAAGGTCCGAACTGTATGTATATATTTTCCCAATAGCAAAGTAGCTCCTACTG 2807

Tr-kit_c18-4_2.7kb CCGCTGCGAAGGTCCGAACTGTATGTATATATTTTCCCAATAGCAAAGTAGCTCCTACTG 811

Tr-kit_c18-4_2.9kb CCGCTGCGAAGGTCCGAACTGTATGTATATATTTTCCCAATAGCAAAGTAGCTCCTACTG 1027

Tr-kit_c18-4_4.0kb CCGCTGCGAAGGTCCGAACTGTATGTATATATTTTCCCAATAGCAAAGTAGCTCCTACTG 2124

Tr-kit_CRL2053_1.9kb CCGCTGCGAAGGTCCGAACTGTATGTATATATTTTCCCAATAGCAAAGTAGCTCCTACTG 1213

Tr-kit_CRL2053_2.7kb CCGCTGCGAAGGTCCGAACTGTATGTATATATTTTCCCAATAGCAAAGTAGCTCCTACTG 1985

Tr-kit_CRL2053_3.1kb CCGCTGCGAAGGTCCGAACTGTATGTATATATTTTCCCAATAGCAAAGTAGCTCCTACTG 1213

Tr-kit_CRL2053_3.9kb CCGCTGCGAAGGTCCGAACTGTATGTATATATTTTCCCAATAGCAAAGTAGCTCCTACTG 1985

************************************************************

NM_001122733.1 TAAACAGAAGGACTCCTCCTGCTTTAGAGGAGAAGGGAAGGGCGGGGTGAAACTGGATGC 3332

NM_021099.3 TAAACAGAAGGACTCCTCCTGCTTTAGAGGAGAAGGGAAGGGCGGGGTGAAACTGGATGC 3316

X65997.1 TAAACAGAAGGACTCCTCCTGCTTTAGAGGAGAAGGGAAGGGCGGGGTGAAACTGGATGC 1350

Full_length TAAACAGAAGGACTCCTCCTGCTTTAGAGGAGAAGGGAAGGGCGGGGTGAAACTGGATGC 3314

Short_3_end_UTR TAAACAGAAGGACTCCTCCTGCTTTAGAGGAGAAGGGAAGGGCGGGGTGAAACTGGATGC 3314

SSCs_specific TAAACAGAAGGACTCCTCCTGCTTTAGAGGAGAAGGGAAGGGCGGGGTGAAACTGGATGC 2867

Tr-kit_c18-4_2.7kb TAAACAGAAGGACTCCTCCTGCTTTAGAGGAGAAGGGAAGGGCGGGGTGAAACTGGATGC 871

Tr-kit_c18-4_2.9kb TAAACAGAAGGACTCCTCCTGCTTTAGAGGAGAAGGGAAGGGCGGGGTGAAACTGGATGC 1087

Tr-kit_c18-4_4.0kb TAAACAGAAGGACTCCTCCTGCTTTAGAGGAGAAGGGAAGGGCGGGGTGAAACTGGATGC 2184

Tr-kit_CRL2053_1.9kb TAAACAGAAGGACTCCTCCTGCTTTAGAGGAGAAGGGAAGGGCGGGGTGAAACTGGATGC 1273

Tr-kit_CRL2053_2.7kb TAAACAGAAGGACTCCTCCTGCTTTAGAGGAGAAGGGAAGGGCGGGGTGAAACTGGATGC 2045

Tr-kit_CRL2053_3.1kb TAAACAGAAGGACTCCTCCTGCTTTAGAGGAGAAGGGAAGGGCGGGGTGAAACTGGATGC 1273

Tr-kit_CRL2053_3.9kb TAAACAGAAGGACTCCTCCTGCTTTAGAGGAGAAGGGAAGGGCGGGGTGAAACTGGATGC 2045

************************************************************

NM_001122733.1 CCAGAGTTCTTCCCCCAGTGCTCCCCTGAGTGTATTTGAAAAGTATGGCCAGTAGTTCAC 3392

NM_021099.3 CCAGAGTTCTTCCCCCAGTGCTCCCCTGAGTGTATTTGAAAAGTATGGCCAGTAGTTCAC 3376

X65997.1 CCAGAGTTCTTCCCCCAGTGCTCCCCTGAGTGTATTTGAAAAGTATGGCCAGTAGTTCAC 1410

Full_length CCAGAGTTCTTCCCCCAGTGCTCCCCTGAGTGTATTTGAAAAGTATGGCCAGTAGTTCAC 3374

Short_3_end_UTR CCAGAGTTCTTCCCCCAGTGCTCCCCTGAGTGTATTTGAAAAGTATGGCCAGTAGTTCAC 3374

SSCs_specific CCAGAGTTCTTCCCCCAGTGCTCCCCTGAGTGTATTTGAAAAGTATGGCCAGTAGTTCAC 2927

Tr-kit_c18-4_2.7kb CCAGAGTTCTTCCCCCAGTGCTCCCCTGAGTGTATTTGAAAAGTATGGCCAGTAGTTCAC 931

Tr-kit_c18-4_2.9kb CCAGAGTTCTTCCCCCAGTGCTCCCCTGAGTGTATTTGAAAAGTATGGCCAGTAGTTCAC 1147

Tr-kit_c18-4_4.0kb CCAGAGTTCTTCCCCCAGTGCTCCCCTGAGTGTATTTGAAAAGTATGGCCAGTAGTTCAC 2244

Tr-kit_CRL2053_1.9kb CCAGAGTTCTTCCCCCAGTGCTCCCCTGAGTGTATTTGAAAAGTATGGCCAGTAGTTCAC 1333

Tr-kit_CRL2053_2.7kb CCAGAGTTCTTCCCCCAGTGCTCCCCTGAGTGTATTTGAAAAGTATGGCCAGTAGTTCAC 2105

Tr-kit_CRL2053_3.1kb CCAGAGTTCTTCCCCCAGTGCTCCCCTGAGTGTATTTGAAAAGTATGGCCAGTAGTTCAC 1333

Tr-kit_CRL2053_3.9kb CCAGAGTTCTTCCCCCAGTGCTCCCCTGAGTGTATTTGAAAAGTATGGCCAGTAGTTCAC 2105

************************************************************

NM_001122733.1 TTGAAGAATAGATGTAGTCCCATTTGGCCCTGAGAGCCATCCTTAATGATGGGAGATATA 3452

NM_021099.3 TTGAAGAATAGATGTAGTCCCATTTGGCCCTGAGAGCCATCCTTAATGATGGGAGATATA 3436

X65997.1 TTGAAGAATAGATGTAGTCCCATTTGGCCCTGAGAGCCATCCTTAATGATGGGAGATATA 1470

Full_length TTGAAGAATAGATGTAGTCCCATTTGGCCCTGAGAGCCATCCTTAATGATGGGAGATATA 3434

Short_3_end_UTR TTGAAGAATAGATGTAGTCCCATTTGGCCCTGAGAGCCATCCTTAATGATGGGAGATATA 3434

SSCs_specific TTGAAGAATAGATGTAGTCCCATTTGGCCCTGAGAGCCATCCTTAATGATGGGAGATATA 2987

Tr-kit_c18-4_2.7kb TTGAAGAATAGATGTAGTCCCATTTGGCCCTGAGAGCCATCCTTAATGATGGGAGATATA 991

Tr-kit_c18-4_2.9kb TTGAAGAATAGATGTAGTCCCATTTGGCCCTGAGAGCCATCCTTAATGATGGGAGATATA 1207

Tr-kit_c18-4_4.0kb TTGAAGAATAGATGTAGTCCCATTTGGCCCTGAGAGCCATCCTTAATGATGGGAGATATA 2304

Tr-kit_CRL2053_1.9kb TTGAAGAATAGATGTAGTCCCATTTGGCCCTGAGAGCCATCCTTAATGATGGGAGATATA 1393

Tr-kit_CRL2053_2.7kb TTGAAGAATAGATGTAGTCCCATTTGGCCCTGAGAGCCATCCTTAATGATGGGAGATATA 2165

Tr-kit_CRL2053_3.1kb TTGAAGAATAGATGTAGTCCCATTTGGCCCTGAGAGCCATCCTTAATGATGGGAGATATA 1393

Tr-kit_CRL2053_3.9kb TTGAAGAATAGATGTAGTCCCATTTGGCCCTGAGAGCCATCCTTAATGATGGGAGATATA 2165

************************************************************

NM_001122733.1 TGTAGCAAGACTAGAAAAGGAAAGCCAAGCCCTTTGTGTAGAAAGCAGACCATTCTTAGA 3512

NM_021099.3 TGTAGCAAGACTAGAAAAGGAAAGCCAAGCCCTTTGTGTAGAAAGCAGACCATTCTTAGA 3496

X65997.1 TGTAGCAAGACTA------GAAAGCCAAGCCCTTTGTGTAGAAAGCAGACCATTCTTAGA 1524

Full_length TGTAGCAAGACTAGAAAAGGAAAGCCAAGCCCTTTGTGTAGAAAGCAGACCATTCTTAGA 3494

Short_3_end_UTR TGTAGCAAGACTAGAAAAGGAAAGCCAAGCCCTTTGTGTAGAAAGCAGACCATTCTTAGA 3494

SSCs_specific TGTAGCAAGACTAGAAAAGGAAAGCCAAGCCCTTTGTGTAGAAAGCAGACCATTCTTAGA 3047

Tr-kit_c18-4_2.7kb TGTAGCAAGACTAGAAAAGGAAAGCCAAGCCCTTTGTGTAGAAAGCAGACCATTCTTAGA 1051

Tr-kit_c18-4_2.9kb TGTAGCAAGACTAGAAAAGGAAAGCCAAGCCCTTTGTGTAGAAAGCAGACCATTCTTAGA 1267

Tr-kit_c18-4_4.0kb TGTAGCAAGACTAGAAAAGGAAAGCCAAGCCCTTTGTGTAGAAAGCAGACCATTCTTAGA 2364

Tr-kit_CRL2053_1.9kb TGTAGCAAGACTAGAAAAGGAAAGCCAAGCCCTTTGTGTAGAAAGCAGACCATTCTTAGA 1453

Tr-kit_CRL2053_2.7kb TGTAGCAAGACTAGAAAAGGAAAGCCAAGCCCTTTGTGTAGAAAGCAGACCATTCTTAGA 2225

Tr-kit_CRL2053_3.1kb TGTAGCAAGACTAGAAAAGGAAAGCCAAGCCCTTTGTGTAGAAAGCAGACCATTCTTAGA 1453

Tr-kit_CRL2053_3.9kb TGTAGCAAGACTAGAAAAGGAAAGCCAAGCCCTTTGTGTAGAAAGCAGACCATTCTTAGA 2225

************* *****************************************

NM_001122733.1 ACAGAGGGCAACGGGGCATCGGAAGTCTGGTCACGCTAAGAAGACCGAGGCTGAGAAGGA 3572

NM_021099.3 ACAGAGGGCAACGGGGCATCGGAAGTCTGGTCACGCTAAGAAGACCGAGGCTGAGAAGGA 3556

X65997.1 ACAGAGGGCAACGGGGCATCGGAAGTCTGGTCACGCTAAGAAGACCGAGGCTGAGAAGGA 1584

Full_length ACAGAGGGCAACGGGGCATCGGAAGTCTGGTCACGCTAAGAAGACCGAGGCTGAGAAGGA 3554

Short_3_end_UTR ACAGAGGGCAACGGGGCATCGGAAGTCTGGTCACGCTAAGAAGACCGAGGCTGAGAAGGA 3554

SSCs_specific ACAGAGGGCAACGGGGCATCGGAAGTCTGGTCACGCTAAGAAGACCGAGGCTGAGAAGGA 3107

Tr-kit_c18-4_2.7kb ACAGAGGGCAACGGGGCATCGGAAGTCTGGTCACGCTAAGAAGACCGAGGCTGAGAAGGA 1111

Tr-kit_c18-4_2.9kb ACAGAGGGCAACGGGGCATCGGAAGTCTGGTCACGCTAAGAAGACCGAGGCTGAGAAGGA 1327

Tr-kit_c18-4_4.0kb ACAGAGGGCAACGGGGCATCGGAAGTCTGGTCACGCTAAGAAGACCGAGGCTGAGAAGGA 2424

Tr-kit_CRL2053_1.9kb ACAGAGGGCAACGGGGCATCGGAAGTCTGGTCACGCTAAGAAGACCGAGGCTGAGAAGGA 1513

Tr-kit_CRL2053_2.7kb ACAGAGGGCAACGGGGCATCGGAAGTCTGGTCACGCTAAGAAGACCGAGGCTGAGAAGGA 2285

Tr-kit_CRL2053_3.1kb ACAGAGGGCAACGGGGCATCGGAAGTCTGGTCACGCTAAGAAGACCGAGGCTGAGAAGGA 1513

Tr-kit_CRL2053_3.9kb ACAGAGGGCAACGGGGCATCGGAAGTCTGGTCACGCTAAGAAGACCGAGGCTGAGAAGGA 2285

************************************************************

NM_001122733.1 ACAAGCCAGGGGAAGCGTGAACAATGATGCTCTGCTCTGGGCTGCCGCTCGGGCTTCTGT 3632

NM_021099.3 ACAAGCCAGGGGAAGCGTGAACAATGATGCTCTGCTCTGGGCTGCCGCTCGGGCTTCTGT 3616

X65997.1 ACAAGCCAGGGGAAGCGTGAACAATGATGCTCTGCTCTGGGCTGCCGCTCGGGCTTCTGT 1644

Full_length ACAAGCCAGGGGAAGCGTGAACAATGATGCTCTGCTCTGGGCTGCCGCTCGGGCTTCTGT 3614

Short_3_end_UTR ACAAGCCAGGGGAAGCGTGAACAATGATGCTCTGCTCTGGGCTGCCGCTCGGGCTTCTGT 3614

SSCs_specific ACAAGCCAGGGGAAGCGTGAACAATGATGCTCTGCTCTGGGCTGCCGCTCGGGCTTCTGT 3167

Tr-kit_c18-4_2.7kb ACAAGCCAGGGGAAGCGTGAACAATGATGCTCTGCTCTGGGCTGCCGCTCGGGCTTCTGT 1171

Tr-kit_c18-4_2.9kb ACAAGCCAGGGGAAGCGTGAACAATGATGCTCTGCTCTGGGCTGCCGCTCGGGCTTCTGT 1387

Tr-kit_c18-4_4.0kb ACAAGCCAGGGGAAGCGTGAACAATGATGCTCTGCTCTGGGCTGCCGCTCGGGCTTCTGT 2484

Tr-kit_CRL2053_1.9kb ACAAGCCAGGGGAAGCGTGAACAATGATGCTCTGCTCTGGGCTGCCGCTCGGGCTTCTGT 1573

Tr-kit_CRL2053_2.7kb ACAAGCCAGGGGAAGCGTGAACAATGATGCTCTGCTCTGGGCTGCCGCTCGGGCTTCTGT 2345

Tr-kit_CRL2053_3.1kb ACAAGCCAGGGGAAGCGTGAACAATGATGCTCTGCTCTGGGCTGCCGCTCGGGCTTCTGT 1573

Tr-kit_CRL2053_3.9kb ACAAGCCAGGGGAAGCGTGAACAATGATGCTCTGCTCTGGGCTGCCGCTCGGGCTTCTGT 2345

************************************************************

NM_001122733.1 ACAACTGACCTGGTTTCTCAGTACTTTGCTGTCTGGGAGTAGCATTGGAATCAAGGCCTC 3692

NM_021099.3 ACAACTGACCTGGTTTCTCAGTACTTTGCTGTCTGGGAGTAGCATTGGAATCAAGGCCTC 3676

X65997.1 ACAACTGACCTGGTTTCTCAGTACTTTGCTGTCTGGGAGTAGCATTGGAATCAAGGCCTC 1704

Full_length ACAACTGACCTGGTTTCTCAGTACTTTGCTGTCTGGGAGTAGCATTGGAATCAAGGCCTC 3674

Short_3_end_UTR ACAACTGACCTGGTTTCTCAGTACTTTGCTGTCTGGGAGTAGCATTGGAATCAAGGCCTC 3674

SSCs_specific ACAACTGACCTGGTTTCTCAGTACTTTGCTGTCTGGGAGTAGCATTGGAATCAAGGCCTC 3227

Tr-kit_c18-4_2.7kb ACAACTGACCTGGTTTCTCAGTACTTTGCTGTCTGGGAGTAGCATTGGAATCAAGGCCTC 1231

Tr-kit_c18-4_2.9kb ACAACTGACCTGGTTTCTCAGTACTTTGCTGTCTGGGAGTAGCATTGGAATCAAGGCCTC 1447

Tr-kit_c18-4_4.0kb ACAACTGACCTGGTTTCTCAGTACTTTGCTGTCTGGGAGTAGCATTGGAATCAAGGCCTC 2544

Tr-kit_CRL2053_1.9kb ACAACTGACCTGGTTTCTCAGTACTTTGCTGTCTGGGAGTAGCATTGGAATCAAGGCCTC 1633

Tr-kit_CRL2053_2.7kb ACAACTGACCTGGTTTCTCAGTACTTTGCTGTCTGGGAGTAGCATTGGAATCAAGGCCTC 2405

Tr-kit_CRL2053_3.1kb ACAACTGACCTGGTTTCTCAGTACTTTGCTGTCTGGGAGTAGCATTGGAATCAAGGCCTC 1633

Tr-kit_CRL2053_3.9kb ACAACTGACCTGGTTTCTCAGTACTTTGCTGTCTGGGAGTAGCATTGGAATCAAGGCCTC 2405

************************************************************

NM_001122733.1 CTCCCTAGTCAGCCTTTGTATATACTCATCTATACGTTGTATGCGTTCATACTTTGGAGG 3752

NM_021099.3 CTCCCTAGTCAGCCTTTGTATATACTCATCTATACGTTGTATGCGTTCATACTTTGGAGG 3736

X65997.1 CTCCCTAGTCAGCCTTTGTATATACTCATCTATACGTTGTATGCGTTCATACTTTGGAGG 1764

Full_length CTCCCTAGTCAGCCTTTGTATATACTCATCTATACGTTGTATGCGTTCATACTTTGGAGG 3734

Short_3_end_UTR CTCCCTAGTCAGCCTTTGTATATACTCATCTATACGTTGTATGCGTTCATACTTTGGAGG 3734

SSCs_specific CTCCCTAGTCAGCCTTTGTATATACTCATCTATACGTTGTATGCGTTCATACTTTGGAGG 3287

Tr-kit_c18-4_2.7kb CTCCCTAGTCAGCCTTTGTATATACTCATCTATACGTTGTATGCGTTCATACTTTGGAGG 1291

Tr-kit_c18-4_2.9kb CTCCCTAGTCAGCCTTTGTATATACTCATCTATACGTTGTATGCGTTCATACTTTGGAGG 1507

Tr-kit_c18-4_4.0kb CTCCCTAGTCAGCCTTTGTATATACTCATCTATACGTTGTATGCGTTCATACTTTGGAGG 2604

Tr-kit_CRL2053_1.9kb CTCCCTAGTCAGCCTTTGTATATACTCATCTATACGTTGTATGCGTTCATACTTTGGAGG 1693

Tr-kit_CRL2053_2.7kb CTCCCTAGTCAGCCTTTGTATATACTCATCTATACGTTGTATGCGTTCATACTTTGGAGG 2465

Tr-kit_CRL2053_3.1kb CTCCCTAGTCAGCCTTTGTATATACTCATCTATACGTTGTATGCGTTCATACTTTGGAGG 1693

Tr-kit_CRL2053_3.9kb CTCCCTAGTCAGCCTTTGTATATACTCATCTATACGTTGTATGCGTTCATACTTTGGAGG 2465

************************************************************

NM_001122733.1 AGGGATTTCCCACAAGCTTTCGTTTCTGTGTACAGCCCTGGCATTAGACCTACTGTGTGT 3812

NM_021099.3 AGGGATTTCCCACAAGCTTTCGTTTCTGTGTACAGCCCTGGCATTAGACCTACTGTGTGT 3796

X65997.1 AGGGATTTCCCACAAGCTTTCGTTTCTGTGTACAGCCCTGG-ATTAGACCTACTGTGTGT 1823

Full_length AGGGATTTCCCACAAGCTTTCGTTTCTGTGTACAGCCCTGGCATTAGACCTACTGTGTGT 3794

Short_3_end_UTR AGGGATTTCCCACAAGCTTTCGTTTCTGTGTACAGCCCTGGCATTAGACCTACTGTGTGT 3794

SSCs_specific AGGGATTTCCCACAAGCTTTCGTTTCTGTGTACAGCCCTGGCATTAGACCTACTGTGTGT 3347

Tr-kit_c18-4_2.7kb AGGGATTTCCCACAAGCTTTCGTTTCTGTGTACAGCCCTGGCATTAGACCTACTGTGTGT 1351

Tr-kit_c18-4_2.9kb AGGGATTTCCCACAAGCTTTCGTTTCTGTGTACAGCCCTGGCATTAGACCTACTGTGTGT 1567

Tr-kit_c18-4_4.0kb AGGGATTTCCCACAAGCTTTCGTTTCTGTGTACAGCCCTGGCATTAGACCTACTGTGTGT 2664

Tr-kit_CRL2053_1.9kb AGGGATTTCCCACAAGCTTTCGTTTCTGTGTACAGCCCTGGCATTAGACCTACTGTGTGT 1753

Tr-kit_CRL2053_2.7kb AGGGATTTCCCACAAGCTTTCGTTTCTGTGTACAGCCCTGGCATTAGACCTACTGTGTGT 2525

Tr-kit_CRL2053_3.1kb AGGGATTTCCCACAAGCTTTCGTTTCTGTGTACAGCCCTGGCATTAGACCTACTGTGTGT 1753

Tr-kit_CRL2053_3.9kb AGGGATTTCCCACAAGCTTTCGTTTCTGTGTACAGCCCTGGCATTAGACCTACTGTGTGT 2525

***************************************** ******************

NM_001122733.1 AAGAATAGATTAAGAGCCATACATATTTGAAGGAAACAGTTAAATGTTTTTTGGTTGTGG 3872

NM_021099.3 AAGAATAGATTAAGAGCCATACATATTTGAAGGAAACAGTTAAATGTTTTTTGGTTGTGG 3856

X65997.1 AAGAATAGATTAAGAGCCATACATATTTGAAGGAAACAGTTAAATGTTTTTTGGTTGTGG 1883

Full_length AAGAATAGATTAAGAGCCATACATATTTGAAGGAAACAGTTAAATGTTTTTTGGTTGTGG 3854

Short_3_end_UTR AAGAATAGATTAAGAGCCATACATATTTGAAGGAAACAGTTAAATGTTTTTTGGTTGTGG 3854

SSCs_specific AAGAATAGATTAAGAGCCATACATATTTGAAGGAAACAGTTAAATGTTTTTTGGTTGTGG 3407

Tr-kit_c18-4_2.7kb AAGAATAGATTAAGAGCCATACATATTTGAAGGAAACAGTTAAATGTTTTTTGGTTGTGG 1411

Tr-kit_c18-4_2.9kb AAGAATAGATTAAGAGCCATACATATTTGAAGGAAACAGTTAAATGTTTTTTGGTTGTGG 1627

Tr-kit_c18-4_4.0kb AAGAATAGATTAAGAGCCATACATATTTGAAGGAAACAGTTAAATGTTTTTTGGTTGTGG 2724

Tr-kit_CRL2053_1.9kb AAGAATAGATTAAGAGCCATACATATTTGAAGGAAACAGTTAAATGTTTTTTGGTTGTGG 1813

Tr-kit_CRL2053_2.7kb AAGAATAGATTAAGAGCCATACATATTTGAAGGAAACAGTTAAATGTTTTTTGGTTGTGG 2585

Tr-kit_CRL2053_3.1kb AAGAATAGATTAAGAGCCATACATATTTGAAGGAAACAGTTAAATGTTTTTTGGTTGTGG 1813

Tr-kit_CRL2053_3.9kb AAGAATAGATTAAGAGCCATACATATTTGAAGGAAACAGTTAAATGTTTTTTGGTTGTGG 2585

************************************************************

NM_001122733.1 TTGTTGTTGTTGTTGTTTTAAAGAAAAAAATGTATATGCTAAGCACAATCTTTATAAGAC 3932

NM_021099.3 TTGTTGTTGTTGTTGTTTTAAAGAAAAAAATGTATATGCTAAGCACAATCTTTATAAGAC 3916

X65997.1 TTGTTGTTGTTGTTGTTTTAAAGAAAAAAATGTATATGCTAAGCACAATCTTTATAAGAC 1943

Full_length TTGTTGTTGTTGTTGTTTTAAAGAAAAAAATGTATATGCTAAGCACAATCTTTATAAGAC 3914

Short_3_end_UTR TTGTTGTTGTTGTTGTTTTAAAGAAAAAAATGTATATGCTAAGCACAATCTTTATAAGAC 3914

SSCs_specific TTGTTGTTGTTGTTGTTTTAAAGAAAAAAATGTATATGCTAAGCACAATCTTTATAAGAC 3467

Tr-kit_c18-4_2.7kb TTGTTGTTGTTGTTGTTTTAAAGAAAAAAATGTATATGCTAAGCACAATCTTTATAAGAC 1471

Tr-kit_c18-4_2.9kb TTGTTGTTGTTGTTGTTTTAAAGAAAAAAATGTATATGCTAAGCACAATCTTTATAAGAC 1687

Tr-kit_c18-4_4.0kb TTGTTGTTGTTGTTGTTTTAAAGAAAAAAATGTATATGCTAAGCACAATCTTTATAAGAC 2784

Tr-kit_CRL2053_1.9kb TTGTTGTTGTTGTTGTTTTAAAGAAAAAAATGTATATGCTAAGCACAATCTTTATAAGAC 1873

Tr-kit_CRL2053_2.7kb TTGTTGTTGTTGTTGTTTTAAAGAAAAAAATGTATATGCTAAGCACAATCTTTATAAGAC 2645

Tr-kit_CRL2053_3.1kb TTGTTGTTGTTGTTGTTTTAAAGAAAAAAATGTATATGCTAAGCACAATCTTTATAAGAC 1873

Tr-kit_CRL2053_3.9kb TTGTTGTTGTTGTTGTTTTAAAGAAAAAAATGTATATGCTAAGCACAATCTTTATAAGAC 2645

************************************************************

NM_001122733.1 CTCTTAGCCAACATACTTGCTCTGTCTACACTTCGGAACAAGCCTTCCATGTCAGAGTGG 3992

NM_021099.3 CTCTTAGCCAACATACTTGCTCTGTCTACACTTCGGAACAAGCCTTCCATGTCAGAGTGG 3976

X65997.1 CTCTTAGCCAACATACTTGCTCTGTCTACACTTCGGAACAAGCCTTCCATGTCAGAGTGG 2003

Full_length CTCTTAGCCAACATACTTGCTCTGTCTACACTTCGGAACAAGCCTTCCATGTCAGAGTGG 3974

Short_3_end_UTR CTCTTAGCCAACA----------------------------------------------- 3927

SSCs_specific CTCTTAGCCAACATACTTGCTCTGTCTACACTTCGGAACAAGCCTTCCATGTCAGAGTGG 3527

Tr-kit_c18-4_2.7kb CTCTTAGCCAACATACTTGCTCTGTCTACACTTCGGAACAAGCCTTCCATGTCAGAGTGG 1531

Tr-kit_c18-4_2.9kb CTCTTAGCCAACATACTTGCTCTGTCTACACTTCGGAACAAGCCTTCCATGTCAGAGTGG 1747

Tr-kit_c18-4_4.0kb CTCTTAGCCAACATACTTGCTCTGTCTACACTTCGGAACAAGCCTTCCATGTCAGAGTGG 2844

Tr-kit_CRL2053_1.9kb CTCTTAGCCAACA----------------------------------------------- 1886

Tr-kit_CRL2053_2.7kb CTCTTAGCCAACA----------------------------------------------- 2658

Tr-kit_CRL2053_3.1kb CTCTTAGCCAACATACTTGCTCTGTCTACACTTCGGAACAAGCCTTCCATGTCAGAGTGG 1933

Tr-kit_CRL2053_3.9kb CTCTTAGCCAACATACTTGCTCTGTCTACACTTCGGAACAAGCCTTCCATGTCAGAGTGG 2705

*************

NM_001122733.1 CTTTGCAGGCAGGAGAACTGAGGCTGTTTGAAAAGGTTACCACAGGATGGAGAAAACAGT 4052

NM_021099.3 CTTTGCAGGCAGGAGAACTGAGGCTGTTTGAAAAGGTTACCACAGGATGGAGAAAACAGT 4036

X65997.1 CTTTGCAGGCAGGAGAACTGAGGCTGTTTGAAAAGGTTACCACAGGATGGAGAAAACAGT 2063

Full_length CTTTGCAGGCAGGAGAACTGAGGCTGTTTGAAAAGGTTACCACAGGATGGAGAAAACAGT 4034

Short_3_end_UTR ----------AAAAAAA-------------AAAA-------AAAAAAAAAAAAAAA---- 3953

SSCs_specific CTTTGCAGGCAGGAGAACTGAGGCTGTTTGAAAAGGTTACCACAGGATGGAGAAAACAGT 3587

Tr-kit_c18-4_2.7kb CTTTGCAGGCAGGAGAACTGAGGCTGTTTGAAAAGGTTACCACAGGATGGAGAAAACAGT 1591

Tr-kit_c18-4_2.9kb CTTTGCAGGCAGGAGAACTGAGGCTGTTTGAAAAGGTTACCACAGGATGGAGAAAACAGT 1807

Tr-kit_c18-4_4.0kb CTTTGCAGGCAGGAGAACTGAGGCTGTTTGAAAAGGTTACCACAGGATGGAGAAAACAGT 2904

Tr-kit_CRL2053_1.9kb ----------AAAAAAA-------------AAAA-------AAAAAAAAAAAAAAA---- 1912

Tr-kit_CRL2053_2.7kb ----------AAAAAAA-------------AAAA-------AAAAAAAAAAAAAAA---- 2684

Tr-kit_CRL2053_3.1kb CTTTGCAGGCAGGAGAACTGAGGCTGTTTGAAAAGGTTACCACAGGATGGAGAAAACAGT 1993

Tr-kit_CRL2053_3.9kb CTTTGCAGGCAGGAGAACTGAGGCTGTTTGAAAAGGTTACCACAGGATGGAGAAAACAGT 2765

* * ** **** * * * * ****

NM_001122733.1 GCAGTCCTGGTTTGGATTCTCACATAGCAGGGAGCACAAGTTAAACTCAGCCTTTTATAG 4112

NM_021099.3 GCAGTCCTGGTTTGGATTCTCACATAGCAGGGAGCACAAGTTAAACTCAGCCTTTTATAG 4096

X65997.1 GCAGTCCTGGTTTGGATTCTCACATAGCAGGGAGCACAAGTTAAACTCGACCTTTTATAG 2123

Full_length GCAGTCCTGGTTTGGATTCTCACATAGCAGGGAGCACAAGTTAAACTCAGCCTTTTATAG 4094

Short_3_end_UTR ------------------------------------------------------------

SSCs_specific GCAGTCCTGGTTTGGATTCTCACATAGCAGGGAGCACAAGTTAAACTCAGCCTTTTATAG 3647

Tr-kit_c18-4_2.7kb GCAGTCCTGGTTTGGATTCTCACATAGCAGGGAGCACAAGTTAAACTCAGCCTTTTATAG 1651

Tr-kit_c18-4_2.9kb GCAGTCCTGGTTTGGATTCTCACATAGCAGGGAGCACAAGTTAAACTCAGCCTTTTATAG 1867

Tr-kit_c18-4_4.0kb GCAGTCCTGGTTTGGATTCTCACATAGCAGGGAGCACAAGTTAAACTCAGCCTTTTATAG 2964

Tr-kit_CRL2053_1.9kb ------------------------------------------------------------

Tr-kit_CRL2053_2.7kb ------------------------------------------------------------

Tr-kit_CRL2053_3.1kb GCAGTCCTGGTTTGGATTCTCACATAGCAGGGAGCACAAGTTAAACTCAGCCTTTTATAG 2053

Tr-kit_CRL2053_3.9kb GCAGTCCTGGTTTGGATTCTCACATAGCAGGGAGCACAAGTTAAACTCAGCCTTTTATAG 2825

NM_001122733.1 GCACGTCCCGGACATCGGGCCAGTATCTATTCAAGTGTGTATGTGTGTGCATGCGTGTGT 4172

NM_021099.3 GCACGTCCCGGACATCGGGCCAGTATCTATTCAAGTGTGTATGTGTGTGCATGCGTGTGT 4156

X65997.1 GCACGTCCCGGACATCGGGCCTGTATCTATTCAAGTGTGTATGTGTGTGCATGCGTGTGT 2183

Full_length GCACGTCCCGGACATCGGGCCAGTATCTATTCAAGTGTGTATGTGTGTGCATGCGTGTGT 4154

Short_3_end_UTR ------------------------------------------------------------

SSCs_specific GCACGTCCCGGACATCGGGCCAGTATCTATTCAAGTGTGTATGTGTGTGCATGCGTGTGT 3707

Tr-kit_c18-4_2.7kb GCACGTCCCGGACATCGGGCCAGTATCTATTCAAGTGTGTATGTGTGTGCATGCGTGTGT 1711

Tr-kit_c18-4_2.9kb GCACGTCCCGGACATCGGGCCAGTATCTATTCAAGTGTGTATGTGTGTGCATGCGTGTGT 1927

Tr-kit_c18-4_4.0kb GCACGTCCCGGACATCGGGCCAGTATCTATTCAAGTGTGTATGTGTGTGCATGCGTGTGT 3024

Tr-kit_CRL2053_1.9kb ------------------------------------------------------------

Tr-kit_CRL2053_2.7kb ------------------------------------------------------------

Tr-kit_CRL2053_3.1kb GCACGTCCCGGACATCGGGCCAGTATCTATTCAAGTGTGTATGTGTGTGCATGCGTGTGT 2113

Tr-kit_CRL2053_3.9kb GCACGTCCCGGACATCGGGCCAGTATCTATTCAAGTGTGTATGTGTGTGCATGCGTGTGT 2885

NM_001122733.1 CTATGCGTGTGGGTGAGTTGTGTTGGGAAACTTGCCCTGCATCCCTGAGGGTCCTCCTTC 4232

NM_021099.3 CTATGCGTGTGGGTGAGTTGTGTTGGGAAACTTGCCCTGCATCCCTGAGGGTCCTCCTTC 4216

X65997.1 CTATGCGTGTGGGTGAGTTGTGTTGGGAAACTTGCCCTGCATCCCTGAGGGTCCTCCTTC 2243

Full_length CTATGCGTGTGGGTGAGTTGTGTTGGGAAACTTGCCCTGCATCCCTGAGGGTCCTCCTTC 4214

Short_3_end_UTR ------------------------------------------------------------

SSCs_specific CTATGCGTGTGGGTGAGTTGTGTTGGGAAACTTGCCCTGCATCCCTGAGGGTCCTCCTTC 3767

Tr-kit_c18-4_2.7kb CTATGCGTGTGGGTGAGTTGTGTTGGGAAACTTGCCCTGCATCCCTGAGGGTCCTCCTTC 1771

Tr-kit_c18-4_2.9kb CTATGCGTGTGGGTGAGTTGTGTTGGGAAACTTGCCCTGCATCCCTGAGGGTCCTCCTTC 1987

Tr-kit_c18-4_4.0kb CTATGCGTGTGGGTGAGTTGTGTTGGGAAACTTGCCCTGCATCCCTGAGGGTCCTCCTTC 3084

Tr-kit_CRL2053_1.9kb ------------------------------------------------------------

Tr-kit_CRL2053_2.7kb ------------------------------------------------------------

Tr-kit_CRL2053_3.1kb CTATGCGTGTGGGTGAGTTGTGTTGGGAAACTTGCCCTGCATCCCTGAGGGTCCTCCTTC 2173

Tr-kit_CRL2053_3.9kb CTATGCGTGTGGGTGAGTTGTGTTGGGAAACTTGCCCTGCATCCCTGAGGGTCCTCCTTC 2945

NM_001122733.1 AGGACCCAAGACGTAACAGCTTCTGTCACCGCTCCTGTCTCTCCAGTTTCCCTGCATGTC 4292

NM_021099.3 AGGACCCAAGACGTAACAGCTTCTGTCACCGCTCCTGTCTCTCCAGTTTCCCTGCATGTC 4276

X65997.1 AGGACCCAAGACGTAACAGCTTCTGTCACCGCTCCTGTCTCTCCAGTTTCCCTGCATGTC 2303

Full_length AGGACCCAAGACGTAACAGCTTCTGTCACCGCTCCTGTCTCTCCAGTTTCCCTGCATGTC 4274

Short_3_end_UTR ------------------------------------------------------------

SSCs_specific AGGACCCAAGACGTAACAGCTTCTGTCACCGCTCCTGTCTCTCCAGTTTCCCTGCATGTC 3827

Tr-kit_c18-4_2.7kb AGGACCCAAGACGTAACAGCTTCTGTCACCGCTCCTGTCTCTCCAGTTTCCCTGCATGTC 1831

Tr-kit_c18-4_2.9kb AGGACCCAAGACGTAACAGCTTCTGTCACCGCTCCTGTCTCTCCAGTTTCCCTGCATGTC 2047

Tr-kit_c18-4_4.0kb AGGACCCAAGACGTAACAGCTTCTGTCACCGCTCCTGTCTCTCCAGTTTCCCTGCATGTC 3144

Tr-kit_CRL2053_1.9kb ------------------------------------------------------------

Tr-kit_CRL2053_2.7kb ------------------------------------------------------------

Tr-kit_CRL2053_3.1kb AGGACCCAAGACGTAACAGCTTCTGTCACCGCTCCTGTCTCTCCAGTTTCCCTGCATGTC 2233

Tr-kit_CRL2053_3.9kb AGGACCCAAGACGTAACAGCTTCTGTCACCGCTCCTGTCTCTCCAGTTTCCCTGCATGTC 3005

NM_001122733.1 GCTCACTGTCTAGAATTTACTCAAAGCCGCCACAGAGGCTTAGCGGAGTGAAGTGCCGAA 4352

NM_021099.3 GCTCACTGTCTAGAATTTACTCAAAGCCGCCACAGAGGCTTAGCGGAGTGAAGTGCCGAA 4336

X65997.1 GCTCACTGTCTAGAATTTACTCAAAGCCGCCACAGAGGCTTAGCGGAGTGAAGTGCCGAA 2363

Full_length GCTCACTGTCTAGAATTTACTCAAAGCCGCCACAGAGGCTTAGCGGAGTGAAGTGCCGAA 4334

Short_3_end_UTR ------------------------------------------------------------

SSCs_specific GCTCACTGTCTAGAATTTACTCAAAGCCGCCACAGAGGCTTAGCGGAGTGAAGTGCCGAA 3887

Tr-kit_c18-4_2.7kb GCTCACTGTCTAGAATTTACTCAAAGCCGCCACAGAGGCTTAGCGGAGTGAAGTGCCGAA 1891

Tr-kit_c18-4_2.9kb GCTCACTGTCTAGAATTTACTCAAAGCCGCCACAGAGGCTTAGCGGAGTGAAGTGCCGAA 2107

Tr-kit_c18-4_4.0kb GCTCACTGTCTAGAATTTACTCAAAGCCGCCACAGAGGCTTAGCGGAGTGAAGTGCCGAA 3204

Tr-kit_CRL2053_1.9kb ------------------------------------------------------------

Tr-kit_CRL2053_2.7kb ------------------------------------------------------------

Tr-kit_CRL2053_3.1kb GCTCACTGTCTAGAATTTACTCAAAGCCGCCACAGAGGCTTAGCGGAGTGAAGTGCCGAA 2293

Tr-kit_CRL2053_3.9kb GCTCACTGTCTAGAATTTACTCAAAGCCGCCACAGAGGCTTAGCGGAGTGAAGTGCCGAA 3065

NM_001122733.1 GGACCTCTTTATTTGGAGTCCTCCTGTATTTAACAACACTCTTATCGTAGACCCATTCAT 4412

NM_021099.3 GGACCTCTTTATTTGGAGTCCTCCTGTATTTAACAACACTCTTATCGTAGACCCATTCAT 4396

X65997.1 GGACCTCTTTATTTGGAGTCCTCCTGTATTTAACAACACTCTTATCGTAGACCCATTCAT 2423

Full_length GGACCTCTTTATTTGGAGTCCTCCTGTATTTAACAACACTCTTATCGTAGACCCATTCAT 4394

Short_3_end_UTR ------------------------------------------------------------

SSCs_specific GGACCTCTTTATTTGGAGTCCTCCTGTATTTAACAACACTCTTATCGTAGACCCATTCAT 3947

Tr-kit_c18-4_2.7kb GGACCTCTTTATTTGGAGTCCTCCTGTATTTAACAACACTCTTATCGTAGACCCATTCAT 1951

Tr-kit_c18-4_2.9kb GGACCTCTTTATTTGGAGTCCTCCTGTATTTAACAACACTCTTATCGTAGACCCATTCAT 2167

Tr-kit_c18-4_4.0kb GGACCTCTTTATTTGGAGTCCTCCTGTATTTAACAACACTCTTATCGTAGACCCATTCAT 3264

Tr-kit_CRL2053_1.9kb ------------------------------------------------------------

Tr-kit_CRL2053_2.7kb ------------------------------------------------------------

Tr-kit_CRL2053_3.1kb GGACCTCTTTATTTGGAGTCCTCCTGTATTTAACAACACTCTTATCGTAGACCCATTCAT 2353

Tr-kit_CRL2053_3.9kb GGACCTCTTTATTTGGAGTCCTCCTGTATTTAACAACACTCTTATCGTAGACCCATTCAT 3125

NM_001122733.1 TAGACCTTATGTAATGCTGCCAATCCAGGGAAACAGATTTAAAGTGTACCCCGTAGACAG 4472

NM_021099.3 TAGACCTTATGTAATGCTGCCAATCCAGGGAAACAGATTTAAAGTGTACCCCGTAGACAG 4456

X65997.1 TAGACCTTATGTAATGCTGCCAATCCAGGGAAACAGATTTAAAGTGTACCCCGTAGACAG 2483

Full_length TAGACCTTATGTAATGCTGCCAATCCAGGGAAACAGATTTAAAGTGTACCCCGTAGACAG 4454

Short_3_end_UTR ------------------------------------------------------------

SSCs_specific TAGACCTTATGTAATGCTGCCAATCCAGGGAAACAGATTTAAAGTGTACCCCGTAGACAG 4007

Tr-kit_c18-4_2.7kb TAGACCTTATGTAATGCTGCCAATCCAGGGAAACAGATTTAAAGTGTACCCCGTAGACAG 2011

Tr-kit_c18-4_2.9kb TAGACCTTATGTAATGCTGCCAATCCAGGGAAACAGATTTAAAGTGTACCCCGTAGACAG 2227

Tr-kit_c18-4_4.0kb TAGACCTTATGTAATGCTGCCAATCCAGGGAAACAGATTTAAAGTGTACCCCGTAGACAG 3324

Tr-kit_CRL2053_1.9kb ------------------------------------------------------------

Tr-kit_CRL2053_2.7kb ------------------------------------------------------------

Tr-kit_CRL2053_3.1kb TAGACCTTATGTAATGCTGCCAATCCAGGGAAACAGATTTAAAGTGTACCCCGTAGACAG 2413

Tr-kit_CRL2053_3.9kb TAGACCTTATGTAATGCTGCCAATCCAGGGAAACAGATTTAAAGTGTACCCCGTAGACAG 3185

NM_001122733.1 GGCCCAGA-GGTTCCCTTGTCCTTGCCCTCCCCCACACCACCCATGATCACTGTCCAACA 4531

NM_021099.3 GGCCCAGA-GGTTCCCTTGTCCTTGCCCTCCCCCACACCACCCATGATCACTGTCCAACA 4515

X65997.1 GGCCCAGA-GGTTCCCTTGTCCTTGCCCTCCCCCACACCACCCATGATCACTGTCCAACA 2542

Full_length GGCCCAGA-GGTTCCCTTGTCCTTGCCCTCCCCCACACCACCCATGATCACTGTCCAACA 4513

Short_3_end_UTR ------------------------------------------------------------

SSCs_specific GGCCCAGAAGGTTCCCTTGTCCTTGCCCTCCCCCACACCACCCATGATCACTGTCCAACA 4067

Tr-kit_c18-4_2.7kb GGCCCAGA-GGTTCCCTTGTCCTTGCCCTCCCCCACACCACCCATGATCACTGTCCAACA 2070

Tr-kit_c18-4_2.9kb GGCCCAGA-GGTTCCCTTGTCCTTGCCCTCCCCCACACCACCCATGATCACTGTCCAACA 2286

Tr-kit_c18-4_4.0kb GGCCCAGA-GGTTCCCTTGTCCTTGCCCTCCCCCACACCACCCATGATCACTGTCCAACA 3383

Tr-kit_CRL2053_1.9kb ------------------------------------------------------------

Tr-kit_CRL2053_2.7kb ------------------------------------------------------------

Tr-kit_CRL2053_3.1kb GGCCCAGA-GGTTCCCTTGTCCTTGCCCTCCCCCACACCACCCATGATCACTGTCCAACA 2472

Tr-kit_CRL2053_3.9kb GGCCCAGA-GGTTCCCTTGTCCTTGCCCTCCCCCACACCACCCATGATCACTGTCCAACA 3244

NM_001122733.1 TAAAGGGTTCAGTGTGT-ACGTGGTCATGTGTTGTCCTTACAGGATTCAGGTATGTTGCC 4590

NM_021099.3 TAAAGGGTTCAGTGTGT-ACGTGGTCATGTGTTGTCCTTACAGGATTCAGGTATGTTGCC 4574

X65997.1 TAAAGGGTTCAGTGTGTTACGTGGTCATGTGTTGTCCTTACAGGATTCAGGTATGTTGCC 2602

Full_length TAAAGGGTTCAGTGTGT-ACGTGGTCATGTGTTGTCCTTACAGGATTCAGGTATGTTGCC 4572

Short_3_end_UTR ------------------------------------------------------------

SSCs_specific TAAAGGGTTCAGTGTGT-ACGTGGTCATGTGTTGTCCTTACAGGATTCAGGTATGTTGCC 4126

Tr-kit_c18-4_2.7kb TAAAGGGTTCAGTGTGT-ACGTGGTCATGTGTTGTCCTTACAGGATTCAGGTATGTTGCC 2129

Tr-kit_c18-4_2.9kb TAAAGGGTTCAGTGTGT-ACGTGGTCATGTGTTGTCCTTACAGGATTCAGGTATGTTGCC 2345

Tr-kit_c18-4_4.0kb TAAAGGGTTCAGTGTGT-ACGTGGTCATGTGTTGTCCTTACAGGATTCAGGTATGTTGCC 3442

Tr-kit_CRL2053_1.9kb ------------------------------------------------------------

Tr-kit_CRL2053_2.7kb ------------------------------------------------------------

Tr-kit_CRL2053_3.1kb TAAAGGGTTCAGTGTGT-ACGTGGTCATGTGTTGTCCTTACAGGATTCAGGTATGTTGCC 2531

Tr-kit_CRL2053_3.9kb TAAAGGGTTCAGTGTGT-ACGTGGTCATGTGTTGTCCTTACAGGATTCAGGTATGTTGCC 3303

NM_001122733.1 TTCACGGTTTTCCCCACCCCCTCCTGCCCTTTATCCTTTAGGCCGTGTGGCCATGAACCT 4650

NM_021099.3 TTCACGGTTTTCCCCACCCCCTCCTGCCCTTTATCCTTTAGGCCGTGTGGCCATGAACCT 4634

X65997.1 TTCACGGTTTTCCCCACCCCCTCCTGCCCTTTATCCTTTAGGCCGTGTGGCCATGAACCT 2662

Full_length TTCACGGTTTTCCCCACCCCCTCCTGCCCTTTATCCTTTAGGCCGTGTGGCCATGAACCT 4632

Short_3_end_UTR ------------------------------------------------------------

SSCs_specific TTCACGGTTTTCCCCACCCCCTCCTGCCCTTTATCCTTTAGGCCGTGTGGCCATGAACCT 4186

Tr-kit_c18-4_2.7kb TTCACGGTTTTCCCCACCCCCTCCTGCCCTTTATCCTTTAGGCCGTGTGGCCATGAACCT 2189

Tr-kit_c18-4_2.9kb TTCACGGTTTTCCCCACCCCCTCCTGCCCTTTATCCTTTAGGCCGTGTGGCCATGAACCT 2405

Tr-kit_c18-4_4.0kb TTCACGGTTTTCCCCACCCCCTCCTGCCCTTTATCCTTTAGGCCGTGTGGCCATGAACCT 3502

Tr-kit_CRL2053_1.9kb ------------------------------------------------------------

Tr-kit_CRL2053_2.7kb ------------------------------------------------------------

Tr-kit_CRL2053_3.1kb TTCACGGTTTTCCCCACCCCCTCCTGCCCTTTATCCTTTAGGCCGTGTGGCCATGAACCT 2591

Tr-kit_CRL2053_3.9kb TTCACGGTTTTCCCCACCCCCTCCTGCCCTTTATCCTTTAGGCCGTGTGGCCATGAACCT 3363

NM_001122733.1 GGAAGAAGTGATCGTTTGCACTTGAGTGCTACACTCTTGCACCTTTCCAAAGTAAGCTGG 4710

NM_021099.3 GGAAGAAGTGATCGTTTGCACTTGAGTGCTACACTCTTGCACCTTTCCAAAGTAAGCTGG 4694

X65997.1 GGAAGAAGTGATCGTTTCGACTTGAGTGCTACACTCTTGCACCTTTCCAAAGTAAGCTGG 2722

Full_length GGAAGAAGTGATCGTTTGCACTTGAGTGCTACACTCTTGCACCTTTCCAAAGTAAGCTGG 4692

Short_3_end_UTR ------------------------------------------------------------

SSCs_specific GGAAGAAGTGATCGTTTGCACTTGAGTGCTACACTCTTGCACCTTTCCAAAGTAAGCTGG 4246

Tr-kit_c18-4_2.7kb GGAAGAAGTGATCGTTTGCACTTGAGTGCTACACTCTTGCACCTTTCCAAAGTAAGCTGG 2249

Tr-kit_c18-4_2.9kb GGAAGAAGTGATCGTTTGCACTTGAGTGCTACACTCTTGCACCTTTCCAAAGTAAGCTGG 2465

Tr-kit_c18-4_4.0kb GGAAGAAGTGATCGTTTGCACTTGAGTGCTACACTCTTGCACCTTTCCAAAGTAAGCTGG 3562

Tr-kit_CRL2053_1.9kb ------------------------------------------------------------

Tr-kit_CRL2053_2.7kb ------------------------------------------------------------

Tr-kit_CRL2053_3.1kb GGAAGAAGTGATCGTTTGCACTTGAGTGCTACACTCTTGCACCTTTCCAAAGTAAGCTGG 2651

Tr-kit_CRL2053_3.9kb GGAAGAAGTGATCGTTTGCACTTGAGTGCTACACTCTTGCACCTTTCCAAAGTAAGCTGG 3423

NM_001122733.1 TTTGGAGGTCCTGTTGTCATGTACGAGACTGTCACCAGTTACCGCGCTCTGTTTGAAACA 4770

NM_021099.3 TTTGGAGGTCCTGTTGTCATGTACGAGACTGTCACCAGTTACCGCGCTCTGTTTGAAACA 4754

X65997.1 TTTGGAGGTCCTGTGGTCATGTACGAGACTGTCACCAGTTACCGCGCTCTGTTTGAAACA 2782

Full_length TTTGGAGGTCCTGTTGTCATGTACGAGACTGTCACCAGTTACCGCGCTCTGTTTGAAACA 4752

Short_3_end_UTR ------------------------------------------------------------

SSCs_specific TTTGGAGGTCCTGTTGTCATGTACGAGACTGTCACCAGTTACCGCGCTCTGTTTGAAACA 4306

Tr-kit_c18-4_2.7kb TTTGGAGGTCCTGTTGTCATGTACGAGACTGTCACCAGTTACCGCGCTCTGTTTGAAACA 2309

Tr-kit_c18-4_2.9kb TTTGGAGGTCCTGTTGTCATGTACGAGACTGTCACCAGTTACCGCGCTCTGTTTGAAACA 2525

Tr-kit_c18-4_4.0kb TTTGGAGGTCCTGTTGTCATGTACGAGACTGTCACCAGTTACCGCGCTCTGTTTGAAACA 3622

Tr-kit_CRL2053_1.9kb ------------------------------------------------------------

Tr-kit_CRL2053_2.7kb ------------------------------------------------------------

Tr-kit_CRL2053_3.1kb TTTGGAGGTCCTGTTGTCATGTACGAGACTGTCACCAGTTACCGCGCTCTGTTTGAAACA 2711

Tr-kit_CRL2053_3.9kb TTTGGAGGTCCTGTTGTCATGTACGAGACTGTCACCAGTTACCGCGCTCTGTTTGAAACA 3483

NM_001122733.1 TGTCTTTGTATTCCTAATGACTTCAGTTAGAGTAAGGAGAATAGCTGTTAATATGGATGT 4830

NM_021099.3 TGTCTTTGTATTCCTAATGACTTCAGTTAGAGTAAGGAGAATAGCTGTTAATATGGATGT 4814

X65997.1 TGTCTTTGTATTCCTAATGACTTCAGTTAGAGTAAGGAGAATAGCTGTTAATATGGATGT 2842

Full_length TGTCTTTGTATTCCTAATGACTTCAGTTAGAGTAAGGAGAATAGCTGTTAATATGGATGT 4812

Short_3_end_UTR ------------------------------------------------------------

SSCs_specific TGTCTTTGTATTCCTAATGACTTCAGTTAGAGTAAGGAGAATAGCTGTTAATATGGATGT 4366

Tr-kit_c18-4_2.7kb TGTCTTTGTATTCCTAATGACTTCAGTTAGAGTAAGGAGAATAGCTGTTAATATGGATGT 2369

Tr-kit_c18-4_2.9kb TGTCTTTGTATTCCTAATGACTTCAGTTAGAGTAAGGAGAATAGCTGTTAATATGGATGT 2585

Tr-kit_c18-4_4.0kb TGTCTTTGTATTCCTAATGACTTCAGTTAGAGTAAGGAGAATAGCTGTTAATATGGATGT 3682

Tr-kit_CRL2053_1.9kb ------------------------------------------------------------

Tr-kit_CRL2053_2.7kb ------------------------------------------------------------

Tr-kit_CRL2053_3.1kb TGTCTTTGTATTCCTAATGACTTCAGTTAGAGTAAGGAGAATAGCTGTTAATATGGATGT 2771

Tr-kit_CRL2053_3.9kb TGTCTTTGTATTCCTAATGACTTCAGTTAGAGTAAGGAGAATAGCTGTTAATATGGATGT 3543

NM_001122733.1 CAGGTACTTAAGGGGCCACACCATTGAGAATTTTGTCTTGGATATTCTTGAAAGTTTATA 4890

NM_021099.3 CAGGTACTTAAGGGGCCACACCATTGAGAATTTTGTCTTGGATATTCTTGAAAGTTTATA 4874

X65997.1 CAGGTACTTAAGGGGCCACACCATTGAGAATTTTGTCTTGGATATTCTTGAAAGTTTATA 2902

Full_length CAGGTACTTAAGGGGCCACACCATTGAGAATTTTGTCTTGGATATTCTTGAAAGTTTATA 4872

Short_3_end_UTR ------------------------------------------------------------

SSCs_specific CAGGTACTTAAGGGGCCACACCATTGAGAATTTTGTCTTGGATATTCTTGAAAGTTTATA 4426

Tr-kit_c18-4_2.7kb CAGGTACTTAAGGGGCCACACCATTGAGAATTTTGTCTTGGATATTCTTGAAAGTTTATA 2429

Tr-kit_c18-4_2.9kb CAGGTACTTAAGGGGCCACACCATTGAGAATTTTGTCTTGGATATTCTTGAAAGTTTATA 2645

Tr-kit_c18-4_4.0kb CAGGTACTTAAGGGGCCACACCATTGAGAATTTTGTCTTGGATATTCTTGAAAGTTTATA 3742

Tr-kit_CRL2053_1.9kb ------------------------------------------------------------

Tr-kit_CRL2053_2.7kb ------------------------------------------------------------

Tr-kit_CRL2053_3.1kb CAGGTACTTAAGGGGCCACACCATTGAGAATTTTGTCTTGGATATTCTTGAAAGTTTATA 2831

Tr-kit_CRL2053_3.9kb CAGGTACTTAAGGGGCCACACCATTGAGAATTTTGTCTTGGATATTCTTGAAAGTTTATA 3603

NM_001122733.1 TTTTTATAATTTTTTTTACATCAGATGTCAGATGTTTCTTTCAGTTGCTTGATGTTTGGA 4950

NM_021099.3 TTTTTATAATTTTTTTTACATCAGATGTCAGATGTTTCTTTCAGTTGCTTGATGTTTGGA 4934

X65997.1 TTTTTATAATTTTTTTTACATCAGATGTCAGATGTTTCTTTCAGTTGCTTGATGTTTGGA 2962

Full_length TTTTTATAATTTTTTTTACATCAGATGTCAGATGTTTCTTTCAGTTGCTTGATGTTTGGA 4932

Short_3_end_UTR ------------------------------------------------------------

SSCs_specific TTTTTATAATTTTTTTTACATCAGATGTCAGATGTTTCTTTCAGTTGCTTGATGTTTGGA 4486

Tr-kit_c18-4_2.7kb TTTTTATAATTTTTTTTACATCAGATGTCAGATGTTTCTTTCAGTTGCTTGATGTTTGGA 2489

Tr-kit_c18-4_2.9kb TTTTTATAATTTTTTTTACATCAGATGTCAGATGTTTCTTTCAGTTGCTTGATGTTTGGA 2705

Tr-kit_c18-4_4.0kb TTTTTATAATTTTTTTTACATCAGATGTCAGATGTTTCTTTCAGTTGCTTGATGTTTGGA 3802

Tr-kit_CRL2053_1.9kb ------------------------------------------------------------

Tr-kit_CRL2053_2.7kb ------------------------------------------------------------

Tr-kit_CRL2053_3.1kb TTTTTATAATTTTTTTTACATCAGATGTCAGATGTTTCTTTCAGTTGCTTGATGTTTGGA 2891

Tr-kit_CRL2053_3.9kb TTTTTATAATTTTTTTTACATCAGATGTCAGATGTTTCTTTCAGTTGCTTGATGTTTGGA 3663

NM_001122733.1 ATTATTATGTGGCTTTTTTTGTAAATATTGAAATGTAGCAATAATGTCTTTTGAATATTC 5010

NM_021099.3 ATTATTATGTGGCTTTTTTTGTAAATATTGAAATGTAGCAATAATGTCTTTTGAATATTC 4994

X65997.1 ATTATTATGTGGCTTTTTTTGTAAATATTGAAATGTAGCAATAATGTCTTTTGAATATTC 3022

Full_length ATTATTATGTGGCTTTTTTTGTAAATATTGAAATGTAGCAATAATGTCTTTTGAATATTC 4992

Short_3_end_UTR ------------------------------------------------------------

SSCs_specific ATTATTATGTGGCTTTTTTTGTAAATATTGAAATGTAGCAATAATGTCTTTTGAATATTC 4546

Tr-kit_c18-4_2.7kb ATTATTATGTGGCTTTTTTTGTAAATATTGAAATGTAGCAATAATGTCTTTTGAATATTC 2549

Tr-kit_c18-4_2.9kb ATTATTATGTGGCTTTTTTTGTAAATATTGAAATGTAGCAATAATGTCTTTTGAATATTC 2765

Tr-kit_c18-4_4.0kb ATTATTATGTGGCTTTTTTTGTAAATATTGAAATGTAGCAATAATGTCTTTTGAATATTC 3862

Tr-kit_CRL2053_1.9kb ------------------------------------------------------------

Tr-kit_CRL2053_2.7kb ------------------------------------------------------------

Tr-kit_CRL2053_3.1kb ATTATTATGTGGCTTTTTTTGTAAATATTGAAATGTAGCAATAATGTCTTTTGAATATTC 2951

Tr-kit_CRL2053_3.9kb ATTATTATGTGGCTTTTTTTGTAAATATTGAAATGTAGCAATAATGTCTTTTGAATATTC 3723

NM_001122733.1 CTGAGCCCATGAGTCCCTGAAAATATTTTTTATATATACAGTAACTTTATGTGTAAATAA 5070

NM_021099.3 CTGAGCCCATGAGTCCCTGAAAATATTTTTTATATATACAGTAACTTTATGTGTAAATAA 5054

X65997.1 CTGAGCCCATGAGTCCCTGAAAATATTTTTTATATATACAGTAACTTTATGTGTAAATAA 3082

Full_length CTGAGCCCATGAGTCCCTGAAAATATTTTTTATATATACAGTAACTTTATGTGTAAATAA 5052

Short_3_end_UTR ------------------------------------------------------------

SSCs_specific CTGAGCCCATGAGTCCCTGAAAATATTTTTTATATATACAGTAACTTTATGTGTAAATAA 4606

Tr-kit_c18-4_2.7kb CTGAGCCCATGAGTCCCTGAAAATATTTTTTATATATACAGTAACTTTATGTGTAAATAA 2609

Tr-kit_c18-4_2.9kb CTGAGCCCATGAGTCCCTGAAAATATTTTTTATATATACAGTAACTTTATGTGTAAATAA 2825

Tr-kit_c18-4_4.0kb CTGAGCCCATGAGTCCCTGAAAATATTTTTTATATATACAGTAACTTTATGTGTAAATAA 3922

Tr-kit_CRL2053_1.9kb ------------------------------------------------------------

Tr-kit_CRL2053_2.7kb ------------------------------------------------------------

Tr-kit_CRL2053_3.1kb CTGAGCCCATGAGTCCCTGAAAATATTTTTTATATATACAGTAACTTTATGTGTAAATAA 3011

Tr-kit_CRL2053_3.9kb CTGAGCCCATGAGTCCCTGAAAATATTTTTTATATATACAGTAACTTTATGTGTAAATAA 3783

NM_001122733.1 TACGCTGTGCAAGTTTAAACATGTCACGTTACATGTGGGTTTTTTCTGATATGTTGTCCA 5130

NM_021099.3 TACGCTGTGCAAGTTTAAACATGTCACGTTACATGTGGGTTTTTTCTGATATGTTGTCCA 5114

X65997.1 TACGCTGTGCAAGTTTAAACATGTCACGTTACATGTGGGTTTTTTCTGATATGTTGTCCA 3142

Full_length TACGCTGTGCAAGTTTAAACATGTCACGTTAC---------------------------- 5084

Short_3_end_UTR ------------------------------------------------------------

SSCs_specific TACGCTGTGCAAGTTTAAACATGTCACGTTAC---------------------------- 4638

Tr-kit_c18-4_2.7kb TACGCTGTGCAAGTTTAAACATGTCACGTTAC---------------------------- 2641

Tr-kit_c18-4_2.9kb TACGCTGTGCAAGTTTAAACATGTCACGTTAC---------------------------- 2857

Tr-kit_c18-4_4.0kb TACGCTGTGCAAGTTTAAACATGTCACGTTAC---------------------------- 3954

Tr-kit_CRL2053_1.9kb ------------------------------------------------------------

Tr-kit_CRL2053_2.7kb ------------------------------------------------------------

Tr-kit_CRL2053_3.1kb TACGCTGTGCAAGTTTAAACATGTCACGTTAC---------------------------- 3043

Tr-kit_CRL2053_3.9kb TACGCTGTGCAAGTTTAAACATGTCACGTTAC---------------------------- 3815

NM_001122733.1 ACTGTTGACAGTTCTGAAGAATTCTAATAAAAATGTAAATATATAAATCAAAAAAAAAAA 5190

NM_021099.3 ACTGTTGACAGTTCTGAAGAATTCTAATAAAAATGTAAATATATAAATCAAAAAAAAAAA 5174

X65997.1 ACTGTTGACAGTTCTGAAGAATTC------------------------------------ 3166

Full_length ------------------------------------------------------------

Short_3_end_UTR ------------------------------------------------------------

SSCs_specific ------------------------------------------------------------

Tr-kit_c18-4_2.7kb ------------------------------------------------------------

Tr-kit_c18-4_2.9kb ------------------------------------------------------------

Tr-kit_c18-4_4.0kb ------------------------------------------------------------

Tr-kit_CRL2053_1.9kb ------------------------------------------------------------

Tr-kit_CRL2053_2.7kb ------------------------------------------------------------

Tr-kit_CRL2053_3.1kb ------------------------------------------------------------

Tr-kit_CRL2053_3.9kb ------------------------------------------------------------

NM_001122733.1 AAAAAAAAAAAAAAA 5205

NM_021099.3 AAAAAAAAAAAAAAA 5189

X65997.1 ---------------

Full_length ---------------

Short_3_end_UTR ---------------

SSCs_specific ---------------

Tr-kit_c18-4_2.7kb ---------------

Tr-kit_c18-4_2.9kb ---------------

Tr-kit_c18-4_4.0kb ---------------

Tr-kit_CRL2053_1.9kb ---------------

Tr-kit_CRL2053_2.7kb ---------------

Tr-kit_CRL2053_3.1kb ---------------

Tr-kit_CRL2053_3.9kb ---------------

**Legend**

**NM_001122733.1**: Mus musculus kit oncogene, transcript variant 1 reference sequence;

**NM_021099.3**: Mus musculus kit oncogene, transcript variant 2 reference sequence;

**X65997.1**: M.musculus *c-kit* mRNA for truncated tyrosine-kinase, Tr-kit;

**Full_length**: *c-kit* full length transcript we got from RACE and sequencing;

**Short_3_end_UTR**: *c-kit* full length transcript with a short 3′ UTR;

**SSCs_specific**: transcript only expressed in undifferentiated spermatogonia;

**Tr-kit_c18-4_2.7kb**: truncated *c-kit* transcript found in c18-4, composed by exons 17-21;

**Tr-kit_c18-4_2.9kb**: truncated *c-kit* transcript found in c18-4, composed by exons 15-21;

**Tr-kit_c18-4_4.0kb**: truncated *c-kit* transcript found in c18-4, composed by intron 17-exon 21;

**Tr-kit_CRL2053_1.9kb**: truncated *c-kit* transcript found in CRL-2053, composed by exons 13-21, with a short 3′ UTR;

**Tr-kit_CRL2053_2.7kb**: truncated *c-kit* transcript found in CRL-2053, composed by exons 1-5 and exons 14-21, with a short 3′ UTR;

**Tr-kit_CRL2053_3.1kb**: truncated *c-kit* transcript found in CRL-2053, composed by exons 13-21;

**Tr-kit_CRL2053_3.9kb**: truncated *c-kit* transcript found in CRL-2053, composed by exons 1-5 and exons 14-21.
