# Supplementary material for: c-kit expression profile and regulatory factors during spermatogonial stem cell differentiation
Source: BMC Dev Biol. 2013 Oct 27;13:38. doi: 10.1186/1471-213X-13-38 (PMC3871025; doi:10.1186/1471-213X-13-38)
Supplement: Additional file 2 — Multiple sequence alignment of ORF finder predicted c-kit proteins. [file 1471-213X-13-38-S2.doc]

**Additional file 2 Multiple sequence alignment of ORF finder predicted *c-kit* proteins**

NP_001116205.1 MRGARGAWDLLCVLLVLLRGQTATSQPSASPGEPSPPSIHPAQSELIVEAGDTLSLTCID 60

NP_066922.2 MRGARGAWDLLCVLLVLLRGQTATSQPSASPGEPSPPSIHPAQSELIVEAGDTLSLTCID 60

CAA46798.1 ------------------------------------------------------------

Full_length MRGARGAWDLLCVLLVLLRGQTATSQPSASPGEPSPPSIHPAQSELIVEAGDTLSLTCID 60

Short_3_end_UTR MRGARGAWDLLCVLLVLLRGQTATSQPSASPGEPSPPSIHPAQSELIVEAGDTLSLTCID 60

SSCs_specific ------------------------------------------------------------

Tr-kit_c18-4_2.7kb ------------------------------------------------------------

Tr-kit_c18-4_2.9kb ------------------------------------------------------------

Tr-kit_c18-4_4.0kb ------------------------------------------------------------

Tr-kit_CRL2053_1.9kb ------------------------------------------------------------

Tr-kit_CRL2053_2.7kb ------------------------------------------------------------

Tr-kit_CRL2053_3.1kb ------------------------------------------------------------

Tr-kit_CRL2053_3.9kb ------------------------------------------------------------

NP_001116205.1 PDFVRWTFKTYFNEMVENKKNEWIQEKAEATRTGTYTCSNSNGLTSSIYVFVRDPAKLFL 120

NP_066922.2 PDFVRWTFKTYFNEMVENKKNEWIQEKAEATRTGTYTCSNSNGLTSSIYVFVRDPAKLFL 120

CAA46798.1 ------------------------------------------------------------

Full_length PDFVRWTFKTYFNEMVENKKNEWIQEKAEATRTGTYTCSNSNGLTSSIYVFVRDPAKLFL 120

Short_3_end_UTR PDFVRWTFKTYFNEMVENKKNEWIQEKAEATRTGTYTCSNSNGLTSSIYVFVRDPAKLFL 120

SSCs_specific ------------------------------------------------------------

Tr-kit_c18-4_2.7kb ------------------------------------------------------------

Tr-kit_c18-4_2.9kb ------------------------------------------------------------

Tr-kit_c18-4_4.0kb ------------------------------------------------------------

Tr-kit_CRL2053_1.9kb ------------------------------------------------------------

Tr-kit_CRL2053_2.7kb ------------------------------------------------------------

Tr-kit_CRL2053_3.1kb ------------------------------------------------------------

Tr-kit_CRL2053_3.9kb ------------------------------------------------------------

NP_001116205.1 VGLPLFGKEDSDALVRCPLTDPQVSNYSLIECDGKSLPTDLTFVPNPKAGITIKNVKRAY 180

NP_066922.2 VGLPLFGKEDSDALVRCPLTDPQVSNYSLIECDGKSLPTDLTFVPNPKAGITIKNVKRAY 180

CAA46798.1 ------------------------------------------------------------

Full_length VGLPLFGKEDSDALVRCPLTDPQVSNYSLIECDGKSLPTDLTFVPNPKAGITIKNVKRAY 180

Short_3_end_UTR VGLPLFGKEDSDALVRCPLTDPQVSNYSLIECDGKSLPTDLTFVPNPKAGITIKNVKRAY 180

SSCs_specific ------------------------------------------------------------

Tr-kit_c18-4_2.7kb ------------------------------------------------------------

Tr-kit_c18-4_2.9kb ------------------------------------------------------------

Tr-kit_c18-4_4.0kb ------------------------------------------------------------

Tr-kit_CRL2053_1.9kb ------------------------------------------------------------

Tr-kit_CRL2053_2.7kb ------------------------------------------------------------

Tr-kit_CRL2053_3.1kb ------------------------------------------------------------

Tr-kit_CRL2053_3.9kb ------------------------------------------------------------

NP_001116205.1 HRLCVRCAAQRDGTWLHSDKFTLKVRAAIKAIPVVSVPETSHLLKKGDTFTVVCTIKDVS 240

NP_066922.2 HRLCVRCAAQRDGTWLHSDKFTLKVRAAIKAIPVVSVPETSHLLKKGDTFTVVCTIKDVS 240

CAA46798.1 ------------------------------------------------------------

Full_length HPLCVRCAAQRDGTWLHSDKFTLKVRAAIKAIPVVSVPETSHLLKKGDTFTVVCTIKDVS 240

Short_3_end_UTR HRLCVRCAAQRDGTWLHSDKFTLKVRAAIKAIPVVSVPETSHLLKKGDTFTVVCTIKDVS 240

SSCs_specific ------------------------------------------------------------

Tr-kit_c18-4_2.7kb ------------------------------------------------------------

Tr-kit_c18-4_2.9kb ------------------------------------------------------------

Tr-kit_c18-4_4.0kb ------------------------------------------------------------

Tr-kit_CRL2053_1.9kb ------------------------------------------------------------

Tr-kit_CRL2053_2.7kb ------------------------------------------------------------

Tr-kit_CRL2053_3.1kb ------------------------------------------------------------

Tr-kit_CRL2053_3.9kb ------------------------------------------------------------

NP_001116205.1 TSVNSMWLKMNPQPQHIAQVKHNSWHRGDFNYERQETLTISSARVDDSGVFMCYANNTFG 300

NP_066922.2 TSVNSMWLKMNPQPQHIAQVKHNSWHRGDFNYERQETLTISSARVDDSGVFMCYANNTFG 300

CAA46798.1 ------------------------------------------------------------

Full_length TSVNSMWLKMNPQPQHIAQVKHNSWHRGDFNYERQETLTISSARVDDSGVFMCYANNTFG 300

Short_3_end_UTR TSVNSMWLKMNPQPQHIAQVKHNSWHRGDFNYERQETLTISSARVDDSGVFMCYANNTFG 300

SSCs_specific ------------------------------------------------------------

Tr-kit_c18-4_2.7kb ------------------------------------------------------------

Tr-kit_c18-4_2.9kb ------------------------------------------------------------

Tr-kit_c18-4_4.0kb ------------------------------------------------------------

Tr-kit_CRL2053_1.9kb ------------------------------------------------------------

Tr-kit_CRL2053_2.7kb ------------------------------------------------------------

Tr-kit_CRL2053_3.1kb ------------------------------------------------------------

Tr-kit_CRL2053_3.9kb ------------------------------------------------------------

NP_001116205.1 SANVTTTLKVVEKGFINISPVKNTTVFVTDGENVDLVVEYEAYPKPEHQQWIYMNRTSAN 360

NP_066922.2 SANVTTTLKVVEKGFINISPVKNTTVFVTDGENVDLVVEYEAYPKPEHQQWIYMNRTSAN 360

CAA46798.1 ------------------------------------------------------------

Full_length SANVTTTLKVVEKGFINISPVKNTTVFVTDGENVDLVVEYEAYPKPEHQQWIYMNRTSAN 360

Short_3_end_UTR SANVTTTLKVVEKGFINISPVKNTTVFVTDGENVDLVVEYEAYPKPEHQQWIYMNRTSAN 360

SSCs_specific ------------------------------------------------------------

Tr-kit_c18-4_2.7kb ------------------------------------------------------------

Tr-kit_c18-4_2.9kb ------------------------------------------------------------

Tr-kit_c18-4_4.0kb ------------------------------------------------------------

Tr-kit_CRL2053_1.9kb ------------------------------------------------------------

Tr-kit_CRL2053_2.7kb ------------------------------------------------------------

Tr-kit_CRL2053_3.1kb ------------------------------------------------------------

Tr-kit_CRL2053_3.9kb ------------------------------------------------------------

NP_001116205.1 KGKDYVKSDNKSNIRYVNQLRLTRLKGTEGGTYTFLVSNSDASASVTFNVYVNTKPEILT 420

NP_066922.2 KGKDYVKSDNKSNIRYVNQLRLTRLKGTEGGTYTFLVSNSDASASVTFNVYVNTKPEILT 420

CAA46798.1 ------------------------------------------------------------

Full_length KGKDYVKSDNKSNIRYVNQLRLTRLKGTEGGTYTFLVSNSDASASVTFNVYVNTKPEILT 420

Short_3_end_UTR KGKDYVKSDNKSNIRYVNQLRLTRLKGTEGGTYTFLVSNSDASASVTFNVYVNTKPEILT 420

SSCs_specific ------------------------------------------------------------

Tr-kit_c18-4_2.7kb ------------------------------------------------------------

Tr-kit_c18-4_2.9kb ------------------------------------------------------------

Tr-kit_c18-4_4.0kb ------------------------------------------------------------

Tr-kit_CRL2053_1.9kb ------------------------------------------------------------

Tr-kit_CRL2053_2.7kb ------------------------------------------------------------

Tr-kit_CRL2053_3.1kb ------------------------------------------------------------

Tr-kit_CRL2053_3.9kb ------------------------------------------------------------

NP_001116205.1 YDRLINGMLQCVAEGFPEPTIDWYFCTGAEQRCTTPVSPVDVQVQNVSVSPFGKLVVQSS 480

NP_066922.2 YDRLINGMLQCVAEGFPEPTIDWYFCTGAEQRCTTPVSPVDVQVQNVSVSPFGKLVVQSS 480

CAA46798.1 ------------------------------------------------------------

Full_length YDRLINGMLQCVAEGFPEPTIDWYFCTGAEQRCTTPVSPVDVQVQNVSVSPFGKLVVQSS 480

Short_3_end_UTR YDRLINGMLQCVAEGFPEPTIDWYFCTGAEQRCTTPVSPVDVQVQNVSVSPFGKLVVQSS 480

SSCs_specific ------------------------------------------------MVPFLAEQIQAH 12

Tr-kit_c18-4_2.7kb ------------------------------------------------------------

Tr-kit_c18-4_2.9kb ------------------------------------------------------------

Tr-kit_c18-4_4.0kb ------------------------------------------------------------

Tr-kit_CRL2053_1.9kb ------------------------------------------------------------

Tr-kit_CRL2053_2.7kb ------------------------------------------------------------

Tr-kit_CRL2053_3.1kb ------------------------------------------------------------

Tr-kit_CRL2053_3.9kb ------------------------------------------------------------

NP_001116205.1 IDSSVFRHNGTVECKASNDVGKSSAFFNFAFKGNNKEQIQAHTLFTPLLIGFVVAAGAMG 540

NP_066922.2 IDSSVFRHNGTVECKASNDVGKSSAFFNFAFK----EQIQAHTLFTPLLIGFVVAAGAMG 536

CAA46798.1 ------------------------------------------------------------

Full_length IDSSVFRHNGTVECKASNDVGKSSAFFNFAFK----EQIQAHTLFTPLLIGFVVAAGAMG 536

Short_3_end_UTR IDSSVFRHNGTVECKASNDVGKSSAFFNFAFK----EQIQAHTLFTPLLIGFVVAAGAMG 536

SSCs_specific TLFTPLLIGFVVAAGAMGIIVMVLTYKYLQVS----IELFSSWGRQGGRAGTDCSAGDT- 67

Tr-kit_c18-4_2.7kb ------------------------------------------------------------

Tr-kit_c18-4_2.9kb ------------------------------------------------------------

Tr-kit_c18-4_4.0kb ------------------------------------------------------------

Tr-kit_CRL2053_1.9kb ------------------------------------------------------------

Tr-kit_CRL2053_2.7kb ------------------------------------------------------------

Tr-kit_CRL2053_3.1kb ------------------------------------------------------------

Tr-kit_CRL2053_3.9kb ------------------------------------------------------------

NP_001116205.1 IIVMVLTYKYLQKPMYEVQWKVVEEINGNNYVYIDPTQLPYDHKWEFPRNRLSFGKTLGA 600

NP_066922.2 IIVMVLTYKYLQKPMYEVQWKVVEEINGNNYVYIDPTQLPYDHKWEFPRNRLSFGKTLGA 596

CAA46798.1 ------------------------------------------------------------

Full_length IIVMVLTYKYLQKPMYEVQWKVVEEINGNNYVYIDPTQLPYDHKWEFPRNRLSFGKTLGA 596

Short_3_end_UTR IIVMVLTYKYLQKPMYEVQWKVVEEINGNNYVYIDPTQLPYDHKWEFPRNRLSFGKTLGA 596

SSCs_specific ----SFFSFLLQKPMYEVQWKVVEEINGNNYVYIDPTQLPYDHKWEFPRNRLSFGKTLGA 123

Tr-kit_c18-4_2.7kb ------------------------------------------------------------

Tr-kit_c18-4_2.9kb ------------------------------------------------------------

Tr-kit_c18-4_4.0kb ------------------------------------------------------------

Tr-kit_CRL2053_1.9kb ------------------------------------------------------------

Tr-kit_CRL2053_2.7kb ------------------------------------------------------------

Tr-kit_CRL2053_3.1kb ------------------------------------------------------------

Tr-kit_CRL2053_3.9kb ------------------------------------------------------------

NP_001116205.1 GAFGKVVEATAYGLIKSDAAMTVAVKMLKPSAHLTEREALMSELKVLSYLGNHMNIVNLL 660

NP_066922.2 GAFGKVVEATAYGLIKSDAAMTVAVKMLKPSAHLTEREALMSELKVLSYLGNHMNIVNLL 656

CAA46798.1 ------------------------------------------------------------

Full_length GAFGKVVEATAYGLIKSDAAMTVAVKMLKPSAHLTEREALMSELKVLSYLGNHMNIVNLL 656

Short_3_end_UTR GAFGKVVEATAYGLIKSDAAMTVAVKMLKPSAHLTEREALMSELKVLSYLGNHMNIVNLL 656

SSCs_specific GAFGKVVEATAYGLIKSDAAMTVAVKMLKPSAHLTEREALMSELKVLSYLGNHMNIVNLL 183

Tr-kit_c18-4_2.7kb ------------------------------------------------------------

Tr-kit_c18-4_2.9kb ------------------------------------------------------------

Tr-kit_c18-4_4.0kb ------------------------------------------------------------

Tr-kit_CRL2053_1.9kb ------------------------------------------------------------

Tr-kit_CRL2053_2.7kb ------------------------------------------------------------

Tr-kit_CRL2053_3.1kb ------------------------------------------------------------

Tr-kit_CRL2053_3.9kb ------------------------------------------------------------

NP_001116205.1 GACTVGGPTLVITEYCCYGDLLNFLRRKRDSFIFSKQEEQAEAALYKNLLHSTEPSCDSS 720

NP_066922.2 GACTVGGPTLVITEYCCYGDLLNFLRRKRDSFIFSKQEEQAEAALYKNLLHSTEPSCDSS 716

CAA46798.1 ------------------------------------------------------------

Full_length GACTVGGPTLVITEYCCYGDLLNFLRRKRDSFIFSKQEEQAEAALYKNLLHSTEPSCDSS 716

Short_3_end_UTR GACTVGGPTLVITEYCCYGDLLNFLRRKRDSFIFSKQEEQAEAALYKNLLHSTEPSCDSS 716

SSCs_specific GACTVGGPTLVITEYCCYGDLLNFLRRKRDSFIFSKQEEQAEAALYKNLLHSTEPSCDSS 243

Tr-kit_c18-4_2.7kb ------------------------------------------------------------

Tr-kit_c18-4_2.9kb ------------------------------------------------------------

Tr-kit_c18-4_4.0kb ------------------------------------------------------------

Tr-kit_CRL2053_1.9kb ------------------------------------------------------------

Tr-kit_CRL2053_2.7kb ----------------------------------------------------------MR 2

Tr-kit_CRL2053_3.1kb ------------------------------------------------------------

Tr-kit_CRL2053_3.9kb ----------------------------------------------------------MR 2

NP_001116205.1 NEYMDMKPGVSYVVPTKTDKRRSARIDSYIERDVTPAIMEDDELALDLDDLLSFSYQVAK 780

NP_066922.2 NEYMDMKPGVSYVVPTKTDKRRSARIDSYIERDVTPAIMEDDELALDLDDLLSFSYQVAK 776

CAA46798.1 --------------------------------------------------------MAVA 4

Full_length NEYMDMKPGVSYVVPTKTDKRRSARIDSYIERDVTPAIMEDDELALDLDDLLSFSYQVAK 776

Short_3_end_UTR NEYMDMKPGVSYVVPTKTDKRRSARIDSYIERDVTPAIMEDDELALDLDDLLSFSYQVAK 776

SSCs_specific NEYMDMKPGVSYVVPTKTDKRRSARIDSYIERDVTPAIMEDDELALDLDDLLSFSYQVAK 303

Tr-kit_c18-4_2.7kb ---------------------------------------------MALRAKWDYIYSSSE 15

Tr-kit_c18-4_2.9kb --------------------------------------MEDDELALDLDDLLSFSYQVAK 22

Tr-kit_c18-4_4.0kb ---------------------------------------------MALRAKWDYIYSSSE 15

Tr-kit_CRL2053_1.9kb ---MDMKPGVSYVVPTKTDKRRSARIDSYIERDVTPAIMEDDELALDLDDLLSFSYQVAK 57

Tr-kit_CRL2053_2.7kb GARGAWDLLCVLLVLLRGQTATSQPSASPGEPSPPSIHPAQSELIVEAGDTLSLTCIDPD 62

Tr-kit_CRL2053_3.1kb ---MDMKPGVSYVVPTKTDKRRSARIDSYIERDVTPAIMEDDELALDLDDLLSFSYQVAK 57

Tr-kit_CRL2053_3.9kb GARGAWDLLCVLLVLLRGQTATSQPSASPGEPSPPSIHPAQSELIVEAGDTLSLTCIDPD 62

NP_001116205.1 GMAFLASKNCIHRDLAARN-ILLTHGRITKICDFGLAR-DIRNDSNYVVKGN------AR 832

NP_066922.2 GMAFLASKNCIHRDLAARN-ILLTHGRITKICDFGLAR-DIRNDSNYVVKGN------AR 828

CAA46798.1 VFPFLP-QQCIHRDLAARN-ILLTHGRITKICDFGLAR-DIRNDSNYVVKGN------AR 55

Full_length GMAFLASKNCIHRDLAARN-ILLTHGRITKICDFGLAR-DIRNDSNYVVKGN------AR 828

Short_3_end_UTR GMAFLASKNCIHRDLAARN-ILLTHGRITKICDFGLAR-DIRNDSNYVVKGN------AR 828

SSCs_specific GMAFLASKNCIHRDLAARN-ILLTHGRITKICDFGLAR-DIRNDSNYVVKGN------AR 355

Tr-kit_c18-4_2.7kb LLAILFKYTQGSTG-----------GRTLGIQFHPALPFSSKAGGVLLFTVG-------- 56

Tr-kit_c18-4_2.9kb GMAFLASKNCIHRDLAARN-ILLTHGRITKICDFGLAR-DIRNDSNYVVKGN------AR 74

Tr-kit_c18-4_4.0kb LLAILFKYTQGSTG-----------GRTLGIQFHPALPFSSKAGGVLLFTVG-------- 56

Tr-kit_CRL2053_1.9kb GMAFLASKNCIHRDLAARN-ILLTHGRITKICDFGLAR-DIRNDSNYVVKGN------AR 109

Tr-kit_CRL2053_2.7kb FVRWTFKTYFNEMVENKKNEWIQEKAEATRTGTYTCSNSNGLTSSIYVFVRDPAKLFLVG 122

Tr-kit_CRL2053_3.1kb GMAFLASKNCIHRDLAARN-ILLTHGRITKICDFGLAR-DIRNDSNYVVKGN------AR 109

Tr-kit_CRL2053_3.9kb FVRWTFKTYFNEMVENKKNEWIQEKAEATRTGTYTCSNSNGLTSSIYVFVRDPAKLFLVG 122

. .. . . .. :. .

NP_001116205.1 LPVKWMAPESIFSCVYTFESDVWSYGIFLWELFSLGS------SPYPGMPVDSKFYKMIK 886

NP_066922.2 LPVKWMAPESIFSCVYTFESDVWSYGIFLWELFSLGS------SPYPGMPVDSKFYKMIK 882

CAA46798.1 LPVKWMAPESIFSCVYTFESDVWSYGIFLWELFSLGS------SPYPGMPVDSKFYKMIK 109

Full_length LPVKWMAPESIFSCVYTFESDVWSYGIFLWELFSLGS------SPYPGMPVDSKFYKMIK 882

Short_3_end_UTR LPVKWMAPESIFSCVYTFESDVWSYGIFLWELFSLGS------SPYPGMPVDSKFYKMIK 882

SSCs_specific LPVKWMAPESIFSCVYTFESDVWSYGIFLWELFSLGS------SPYPGMPVDSKFYKMIK 409

Tr-kit_c18-4_2.7kb --------ATLLLGKYIHTVRTFAAGRWLMAEKKRPS--------------------ATK 88

Tr-kit_c18-4_2.9kb LPVKWMAPESIFSCVYTFESDVWSYGIFLWELFSLGS------SPYPGMPVDSKFYKMIK 128

Tr-kit_c18-4_4.0kb --------ATLLLGKYIHTVRTFAAGRWLMAEKKRPS--------------------ATK 88

Tr-kit_CRL2053_1.9kb LPVKWMAPESIFSCVYTFESDVWSYGIFLWELFSLGS------SPYPGMPVDSKFYKMIK 163

Tr-kit_CRL2053_2.7kb LPLFGKEDSDALVRCPLTDPQVSNYSLIECDGKSLPTDLTFVPNPKAGITIKNVKRAYHR 182

Tr-kit_CRL2053_3.1kb LPVKWMAPESIFSCVYTFESDVWSYGIFLWELFSLGS------SPYPGMPVDSKFYKMIK 163

Tr-kit_CRL2053_3.9kb LPLFGKEDSDALVRCPLTDPQVSNYSLIECDGKSLPTDLTFVPNPKAGITIKNVKRAYHR 182

: . . . : :

NP_001116205.1 EGFRMVSPEHAPAEMYD--VMKTCWDADPLKRPTFKQVVQLIEK--------QISDSTKH 936

NP_066922.2 EGFRMVSPEHAPAEMYD--VMKTCWDADPLKRPTFKQVVQLIEK--------QISDSTKH 932

CAA46798.1 EGFRMVSPEHAPAEMYD--VMKTCWDADPLKRPTFKQVVQLIEK--------QISDSTKH 159

Full_length EGFRMVSPEHAPAEMYD--VMKTCWDADPLKRPTFKQVVQLIEK--------QISDSTKH 932

Short_3_end_UTR EGFRMVSPEHAPAEMYD--VMKTCWDADPLKRPTFKQVVQLIEK--------QISDSTKH 932

SSCs_specific EGFRMVSPEHAPAEMYD--VMKTCWDADPLKRPTFKQVVQLIEK--------QISDSTKH 459

Tr-kit_c18-4_2.7kb VCARKGGITVG---------LEVGWGTR------YPGIGCKSKG------------NQVT 121

Tr-kit_c18-4_2.9kb EGFRMVSPEHAPAEMYD--VMKTCWDADPLKRPTFKQVVQLIEK--------QISDSTKH 178

Tr-kit_c18-4_4.0kb VCARKGGITVG---------LEVGWGTR------YPGIGCKSKG------------NQVT 121

Tr-kit_CRL2053_1.9kb EGFRMVSPEHAPAEMYD--VMKTCWDADPLKRPTFKQVVQLIEK--------QISDSTKH 213

Tr-kit_CRL2053_2.7kb LCVRCAAQRDGTWLHSDKFTLKVRAAIKAIPVVSVPETSHLLKKGDTFTVVCTIKDVSTS 242

Tr-kit_CRL2053_3.1kb EGFRMVSPEHAPAEMYD--VMKTCWDADPLKRPTFKQVVQLIEK--------QISDSTKH 213

Tr-kit_CRL2053_3.9kb LCVRCAAQRDGTWLHSDKFTLKVRAAIKAIPVVSVPETSHLLKKGDTFTVVCTIKDVSTS 242

* . . ::. :

NP_001116205.1 IYSNLANCNPNPENPVVVDHSVRVNSVGSSASSTQPLLVHEDA-- 979

NP_066922.2 IYSNLANCNPNPENPVVVDHSVRVNSVGSSASSTQPLLVHEDA-- 975

CAA46798.1 IYSNLANCNPNPENPVVVDHSVRVNSVGSSASSTQPLLVHEDA-- 202

Full_length IYSNLANCNPNPENPVVVDHSVRVNSVGSSASSTQPLLVHEDA-- 975

Short_3_end_UTR IYSNLANCNPNPENPVVVDHSVRVNSVGSSASSTQPLLVHEDA-- 975

SSCs_specific IYSNLANCNPNPENPVVVDHSVRVNSVGSSASSTQPLLVHEDA-- 502

Tr-kit_c18-4_2.7kb ITEARRTGSETAAKPVGLGFLLRHLRARAGAA------------- 153

Tr-kit_c18-4_2.9kb IYSNLANCNPNPENPVVVDHSVRVNSVGSSASSTQPLLVHEDA-- 221

Tr-kit_c18-4_4.0kb ITEARRTGSETAAKPVGLGFLLRHLRARAGAA------------- 153

Tr-kit_CRL2053_1.9kb IYSNLANCNPNPENPVVVDHSVRVNSVGSSASSTQPLLVHEDA-- 256

Tr-kit_CRL2053_2.7kb VNSMWLKMNPQPQHIAQKSRQKRHFIRTFCTQRSLPVTVQMNIWT 287

Tr-kit_CRL2053_3.1kb IYSNLANCNPNPENPVVVDHSVRVNSVGSSASSTQPLLVHEDA-- 256

Tr-kit_CRL2053_3.9kb VNSMWLKMNPQPQHIAQKSRQKRHFIRTFCTQRSLPVTVQMNIWT 287

: . . . . : . . * :

**Legend**

**NP_001116205.1**: mast/stem cell growth factor receptor isoform 1 [Mus musculus];

**NP_066922.2**: mast/stem cell growth factor receptor isoform 2 [Mus musculus];

**CAA46798.1**: truncated tyrosine kinase receptor [Mus musculus];

**Full_length**: protein predicted by ORF finder with the *c-kit* full length transcript we got from RACE and sequencing;

**Short_3_end_UTR**: protein predicted by ORF finder with the *c-kit* full length transcript with a short 3′ UTR;

**SSCs_specific**: protein predicted by ORF finder with the SSCs specific transcript.;

**Tr-kit_c18-4_2.7kb**: protein predicted by ORF finder with the 2.7 kb truncated *c-kit* transcript found in c18-4;

**Tr-kit_c18-4_2.9kb**: protein predicted by ORF finder with the 2.9 kb truncated *c-kit* transcript found in c18-4;

**Tr-kit_c18-4_4.0kb**: protein predicted by ORF finder with the 4.0 kb truncated *c-kit* transcript found in c18-4;

**Tr-kit_Tr-kit_CRL2053_1.9kb**: protein predicted by ORF finder with the 1.9 kb truncated *c-kit* transcript with a short 3′ UTR found in CRL-2053;

**Tr-kit_Tr-kit_CRL2053_2.7kb**: protein predicted by ORF finder with the 2.7 kb truncated *c-kit* transcript with a short 3′ UTR found in CRL-2053;

**Tr-kit_CRL2053_3.1kb**: protein predicted by ORF finder with the 3.1 kb truncated *c-kit* transcript found in CRL-2053;

**Tr-kit_CRL2053_3.9kb**: protein predicted by ORF finder with the 3.9 kb truncated *c-kit* transcript found in CRL-2053.

Transmembrane domain protein sequence was labeled with red color. Before the transmembrane domain is the extracellular domain; after the transmembrane domain is the intracellular domain.
